# Supplementary material for: A practical solution to pseudoreplication bias in single-cell studies
Source: Nat Commun. 2021 Feb 2;12:738. doi: 10.1038/s41467-021-21038-1 (PMC7854630; doi:10.1038/s41467-021-21038-1)
Supplement: Supplementary file 1 — Supplementary Information [file 41467_2021_21038_MOESM1_ESM.pdf]

Supplementary Materials for

A practical solution to pseudoreplication bias in single-cell studies

Kip D. Zimmerman, Mark A. Espeland, Carl D. Langefeld

Correspondence to: kdzimmer@wakehealth.edu or clangefe@wakehealth.edu

This PDF file includes:

Supplementary Figures 1 to 11
Supplementary Tables 1 to 6

Real data - Pancreatic alpha cells
(10 samples, ~100 cells per sample)

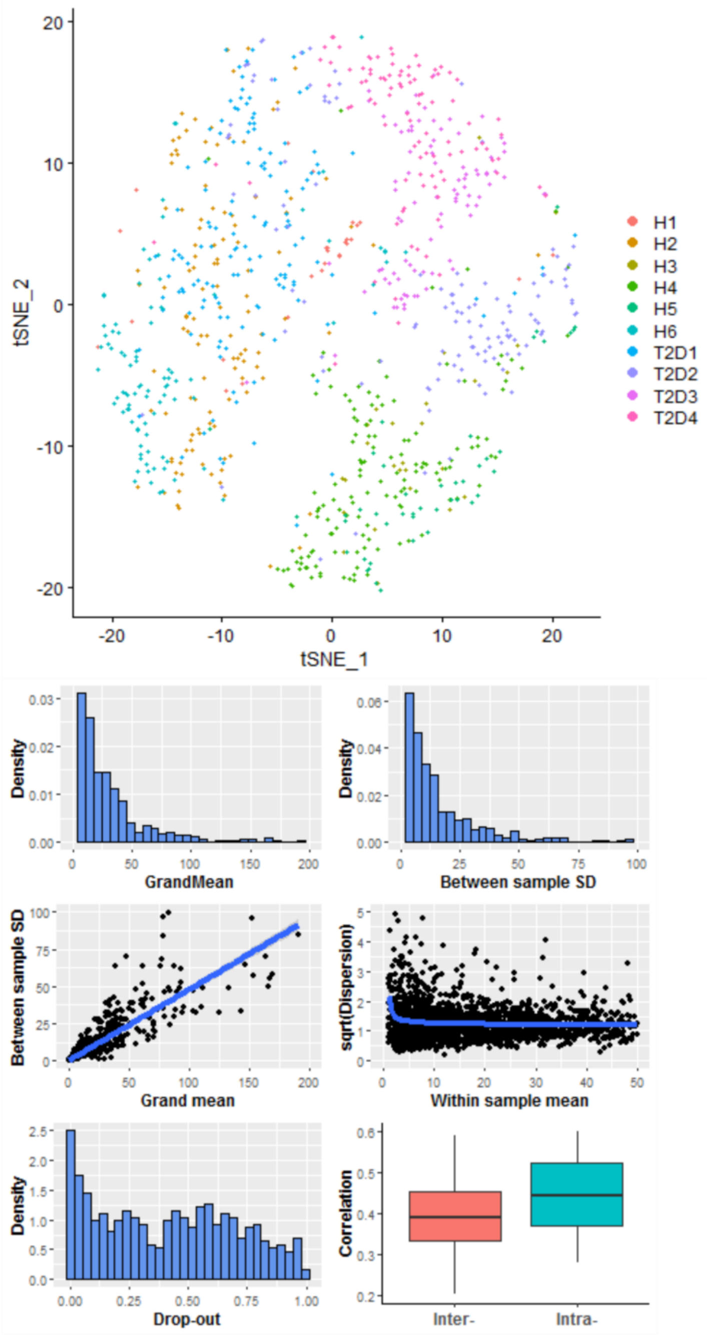

Simulated data
(10 samples, ~100 cells per sample)

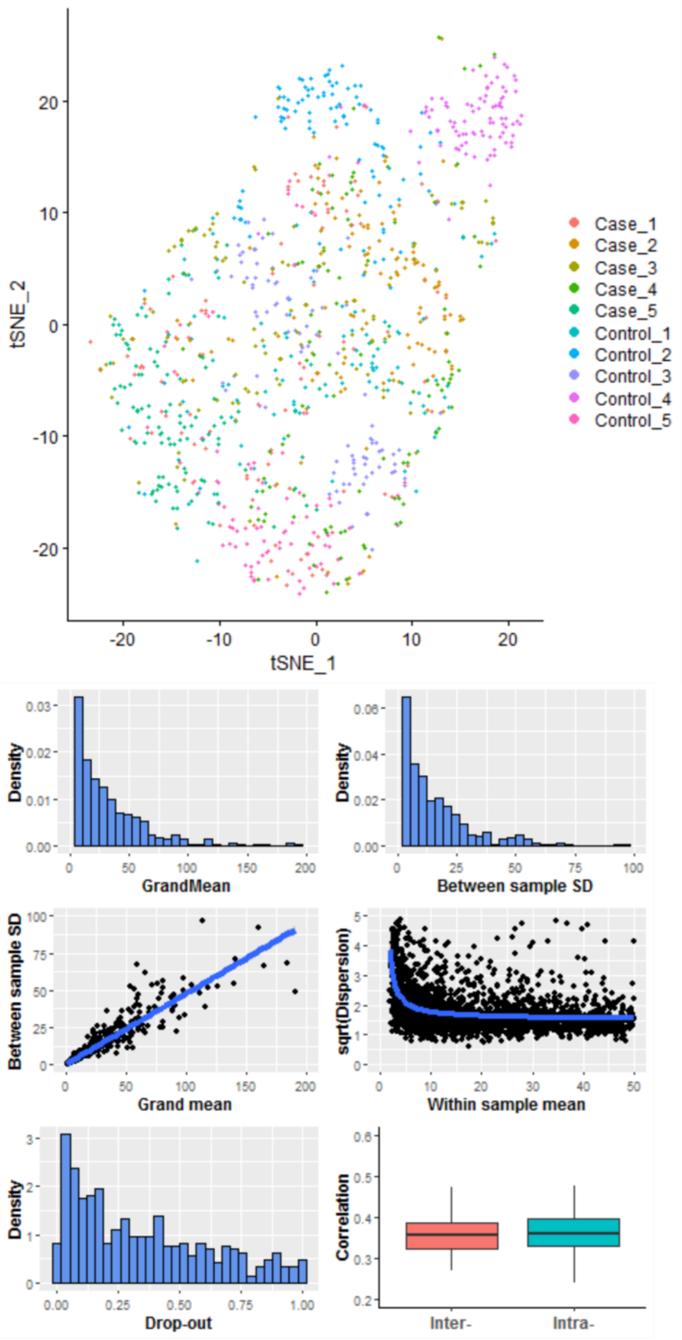

Supplementary Figure 1: Similarities between simulated data and real data. tSNE plots and plots of various parameters demonstrate similarities between simulated and real data. All axes, except tSNE plot axes, are held constant across the two sets of panels. Parameters were estimated on pancreatic alpha cells with 500 genes correlated with a Spearman's coefficient < 0.25. The simulated data consist of 500 independently simulated genes. Top panels: tSNE plots of the data. The next panel below: histograms of the grand mean and between-sample standard deviation. Middle set of panels: relationships between the grand mean and the between-sample standard deviation and relationships between intra-individual means and dispersion. Bottom set of panels: dropout results and the boxplot demonstrate distributions of intra-individual (blue) and inter-individual (red) correlations. Differences between inter- and intra-individual correlations are less exaggerated in our simulated data. The center line represents the median. The lower and upper box limits represent the 25% and 75% quantiles, respectively. The whiskers extend to the largest observation within the box limit plus or minus one and a half multiplied by the interquartile range.

Real data - Pancreatic ductal cells
(10 samples, ~50 cells per sample)

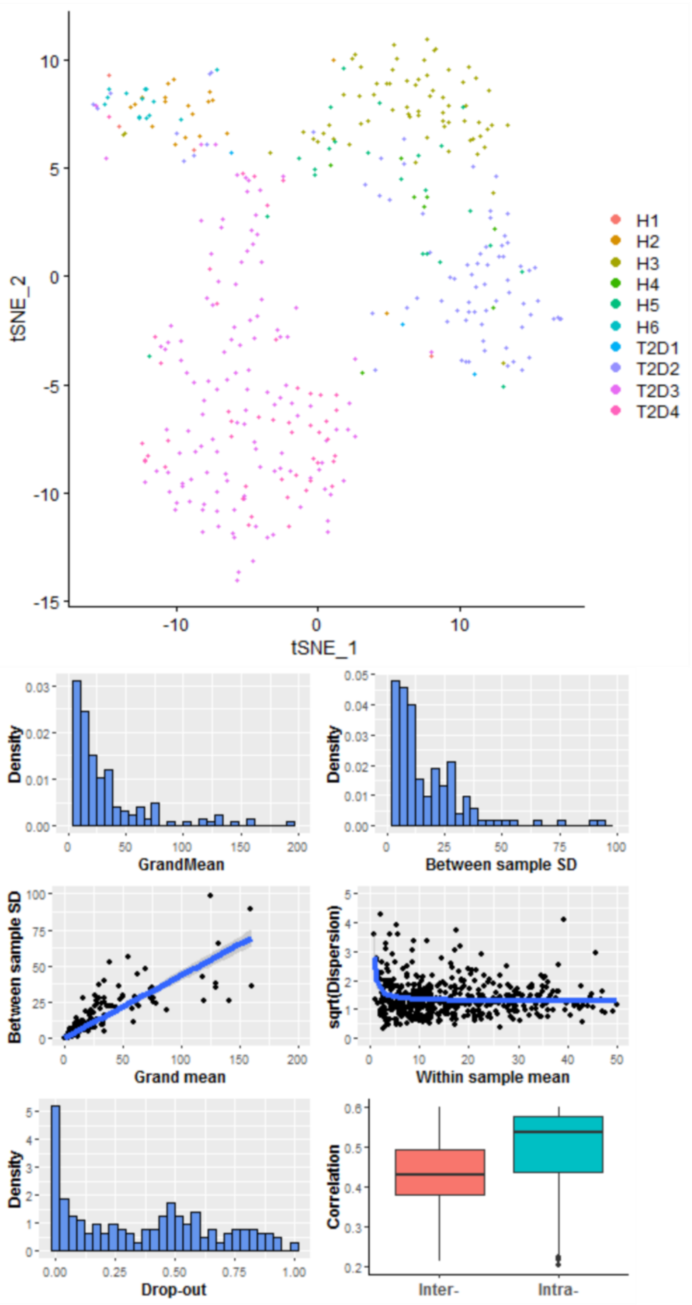

Simulated data
(10 samples, ~50 cells per sample)

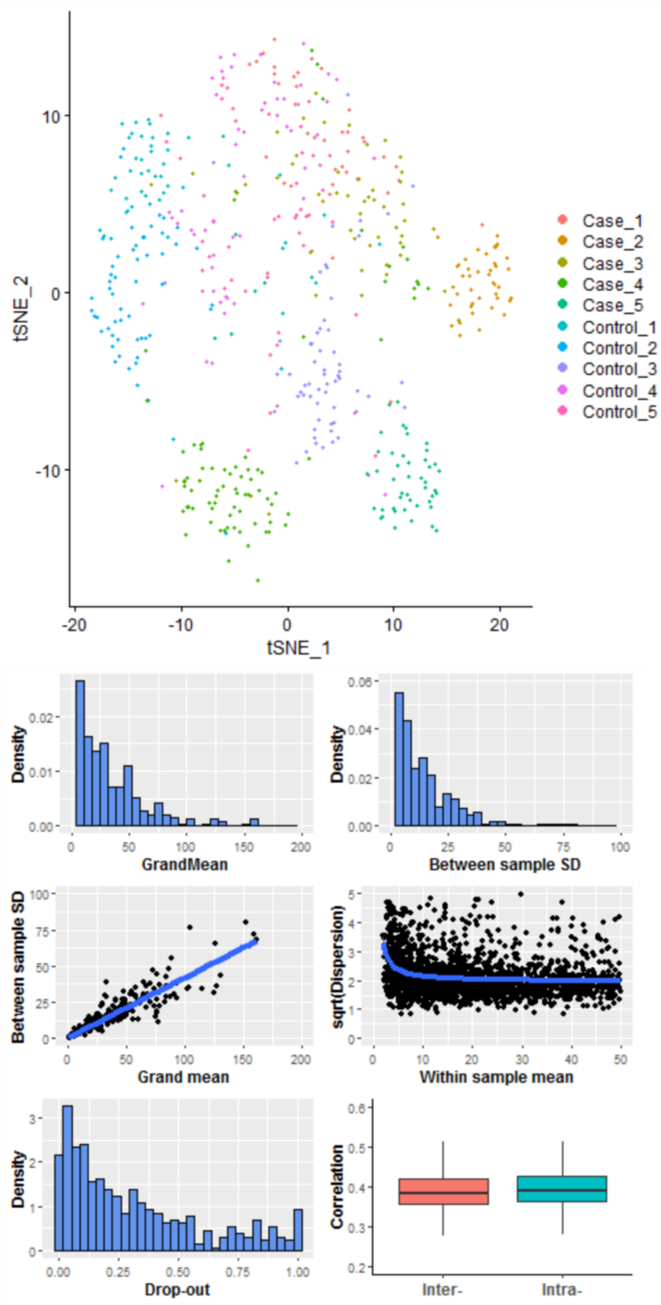

Supplementary Figure 2: Similarities between simulated data and real data. tSNE plots and plots of various parameters demonstrate similarities between simulated and real data. All axes, except tSNE plot axes, are held constant across the two sets of panels. Parameters were estimated on pancreatic ductal cells with 500 genes correlated with a Spearman's coefficient < 0.25. The simulated data consist of 500 independently simulated genes. Top panels: tSNE plots of the data. The next panel below: histograms of the grand mean and between-sample standard deviation. Middle set of panels: relationships between the grand mean and the between-sample standard deviation and relationships between intra-individual means and dispersion. Bottom set of panels: dropout results and the boxplot demonstrate distributions of intra-individual (blue) and inter-individual (red) correlations. Differences between inter- and intra-individual correlations are less exaggerated in our simulated data. The center line represents the median. The lower and upper box limits represent the 25% and 75% quantiles, respectively. The whiskers extend to the largest observation within the box limit plus or minus one and a half multiplied by the interquartile range.

Real data - Pancreatic beta cells
(10 samples, ~30 cells per sample)

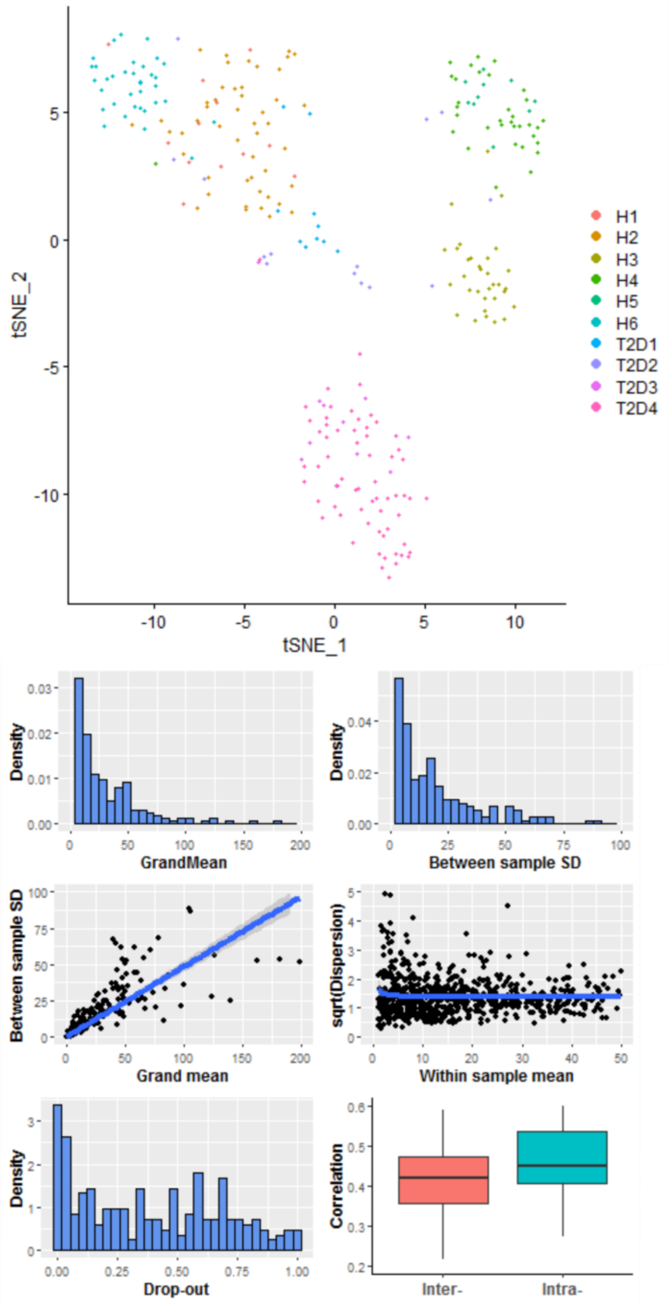

Simulated data
(10 samples, ~30 cells per sample)

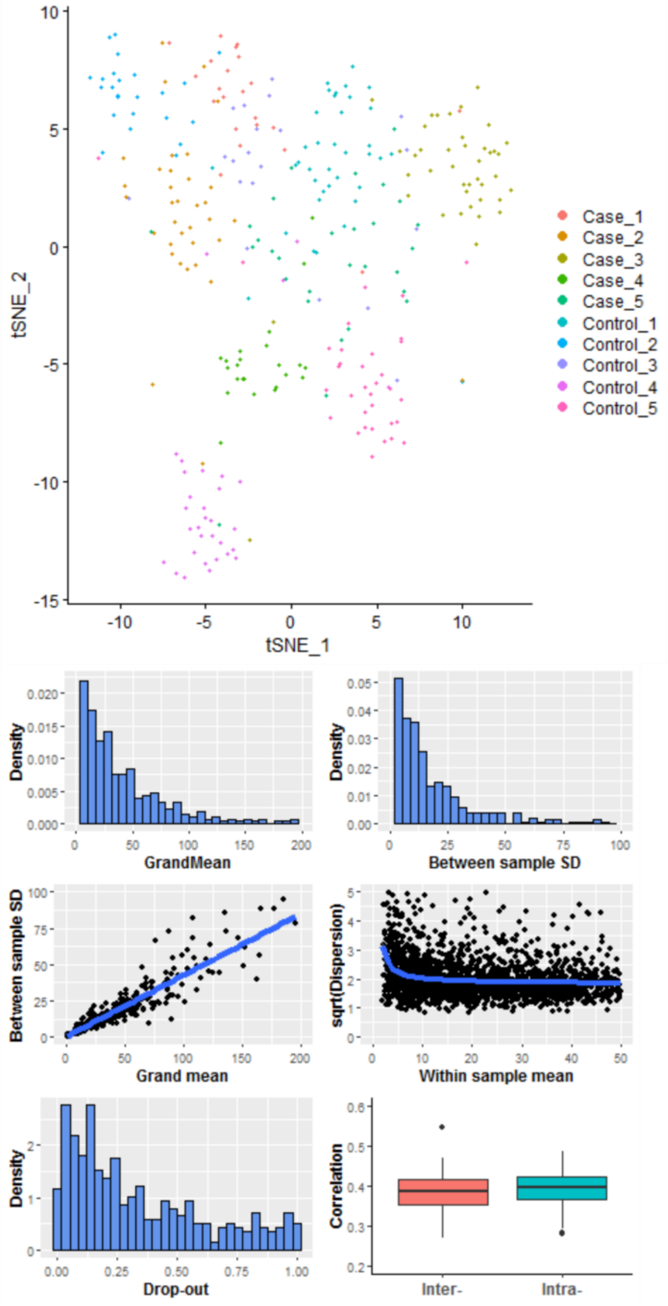

Supplementary Fig. 3: Similarities between simulated data and real data. tSNE plots and plots of various parameters demonstrate similarities between simulated and real data. All axes, except tSNE plot axes, are held constant across the two sets of panels. Parameters were estimated on pancreatic beta cells with 500 genes correlated with a Spearman's coefficient < 0.25. The simulated data consist of 500 independently simulated genes. Top panels: tSNE plots of the data. The next panel below: histograms of the grand mean and between-sample standard deviation. Middle set of panels: relationships between the grand mean and the between-sample standard deviation and relationships between intra-individual means and dispersion. Bottom set of panels: dropout results and the boxplot demonstrate distributions of intra-individual (blue) and inter-individual (red) correlations. Differences between inter- and intra-individual correlations are less exaggerated in our simulated data. The center line represents the median. The lower and upper box limits represent the 25% and 75% quantiles, respectively. The whiskers extend to the largest observation within the box limit plus or minus one and a half multiplied by the interquartile range.

Real data - Mucosal cells
(6 samples, ~30 cells per sample)

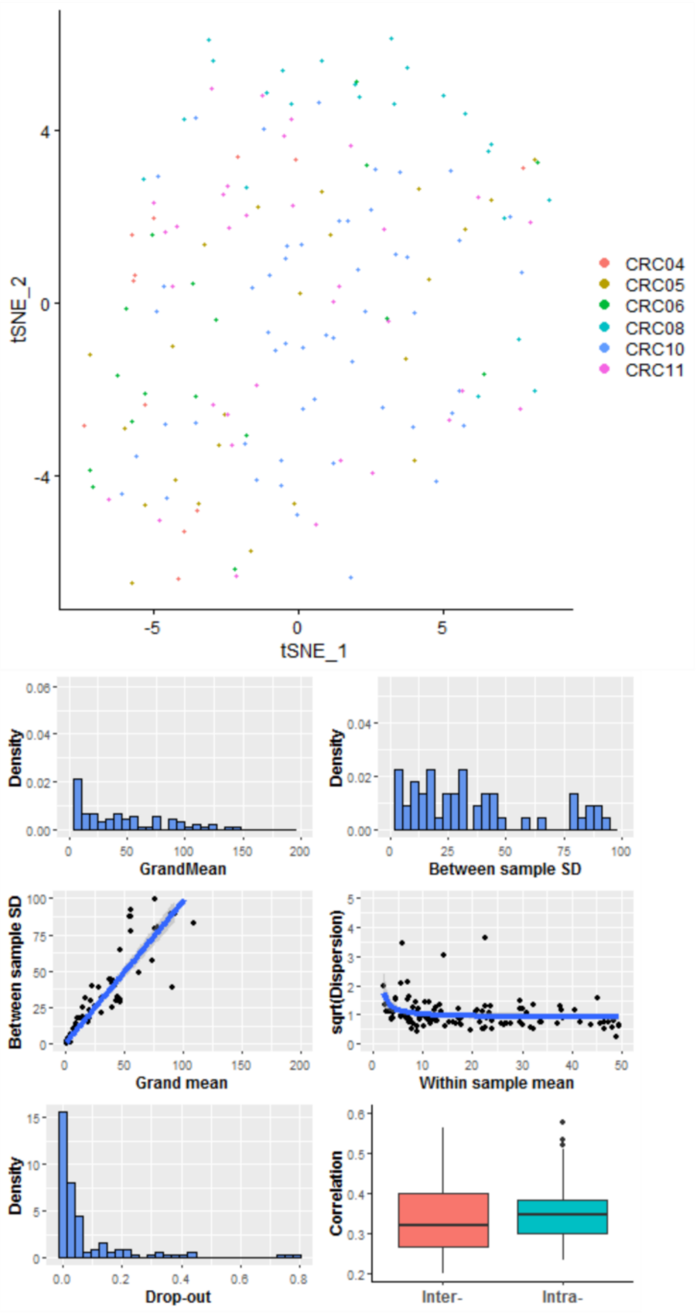

Simulated data
(6 samples, ~30 cells per sample)

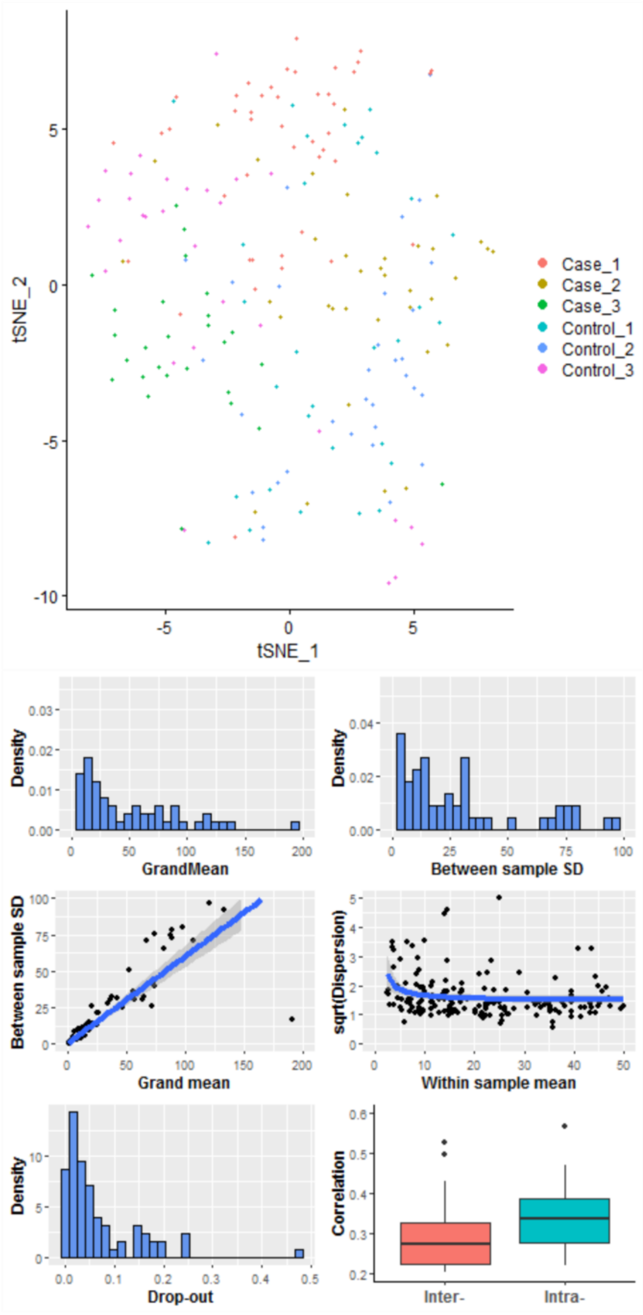

Supplementary Fig. 4: **Similarities between simulated data and real data**. tSNE plots and plots of various parameters demonstrate similarities between simulated and real data. All axes, except tSNE plot axes, are held constant across the two sets of panels. Parameters were estimated on normal mucosal cells with 215 genes correlated with a Spearman's coefficient < 0.25. The simulated data consist of 215 independently simulated genes. Top panels: tSNE plots of the data. The next panel below: histograms of the grand mean and between-sample standard deviation. Middle set of panels: relationships between the grand mean and the between-sample standard deviation and relationships between intra-individual means and dispersion. Bottom set of panels: dropout results and the boxplot demonstrate distributions of intra-individual (blue) and inter-individual (red) correlations. Differences between inter- and intra-individual correlations are less exaggerated in our simulated data. The center line represents the median. The lower and upper box limits represent the 25% and 75% quantiles, respectively. The whiskers extend to the largest observation within the box limit plus or minus one and a half multiplied by the interquartile range.

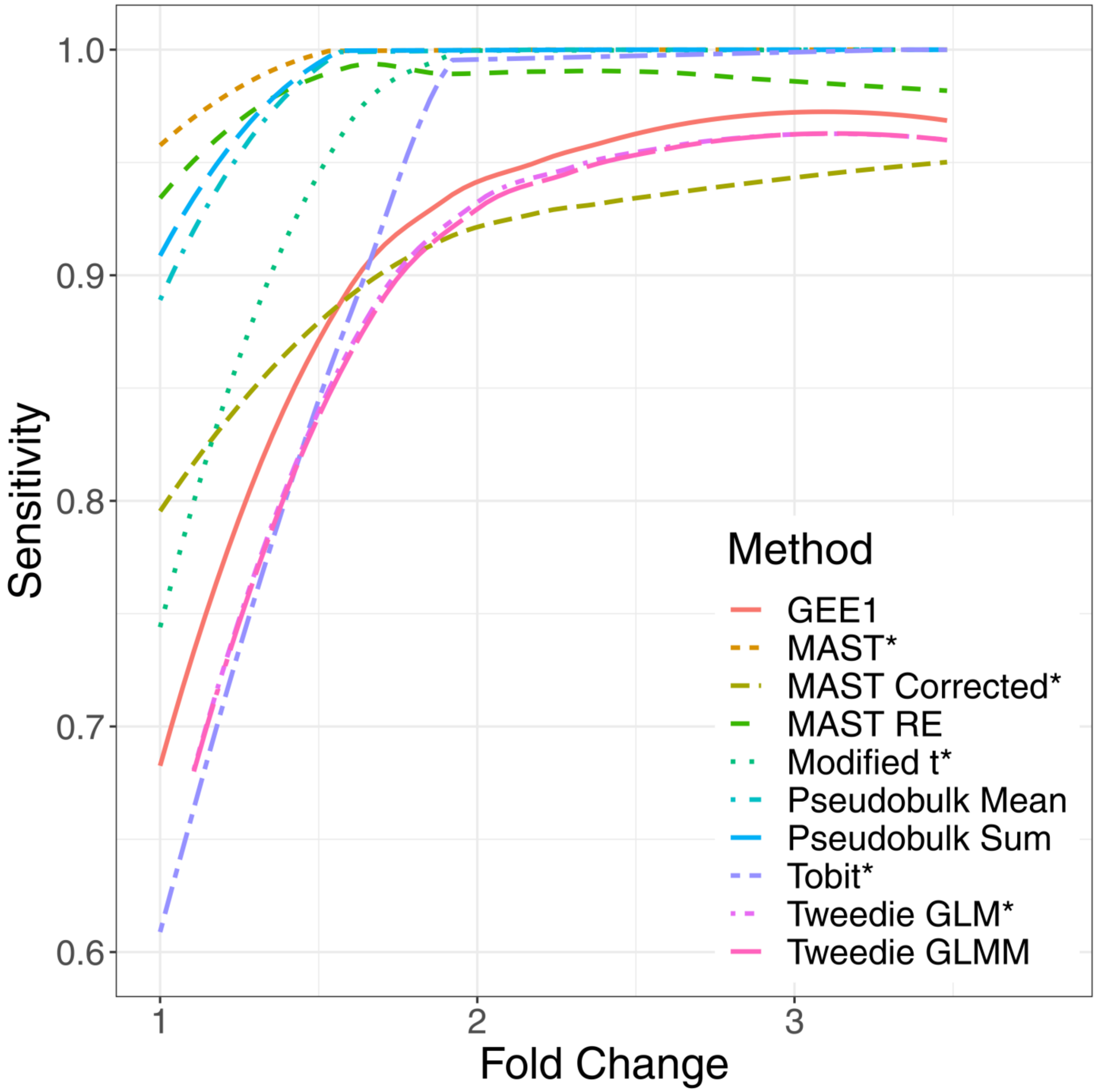

Supplementary Fig. 5: Sensitivity at varying fold changes for each method. Estimates of sensitivity for each method are provided for increasing values of fold change. MAST was implemented without random effects, MAST RE denotes MAST was implemented with random effects, MAST Corrected denotes data were batch-corrected for individual prior to analysis without using individual as a random effect, GLM denotes generalized linear model, and GLMM denotes generalized linear mixed-effects model. Tweedie model as implemented in ‘glmmTMB’, GEE1 as implemented in ‘geepack’, Pseudobulk approaches averaged or summed across cells within an individual and were implemented in DESeq2, Modified t as implemented in ROTS, and Tobit as implemented in Monocle. *These methods exhibit inflated type 1 error rates and therefore inflated sensitivity.

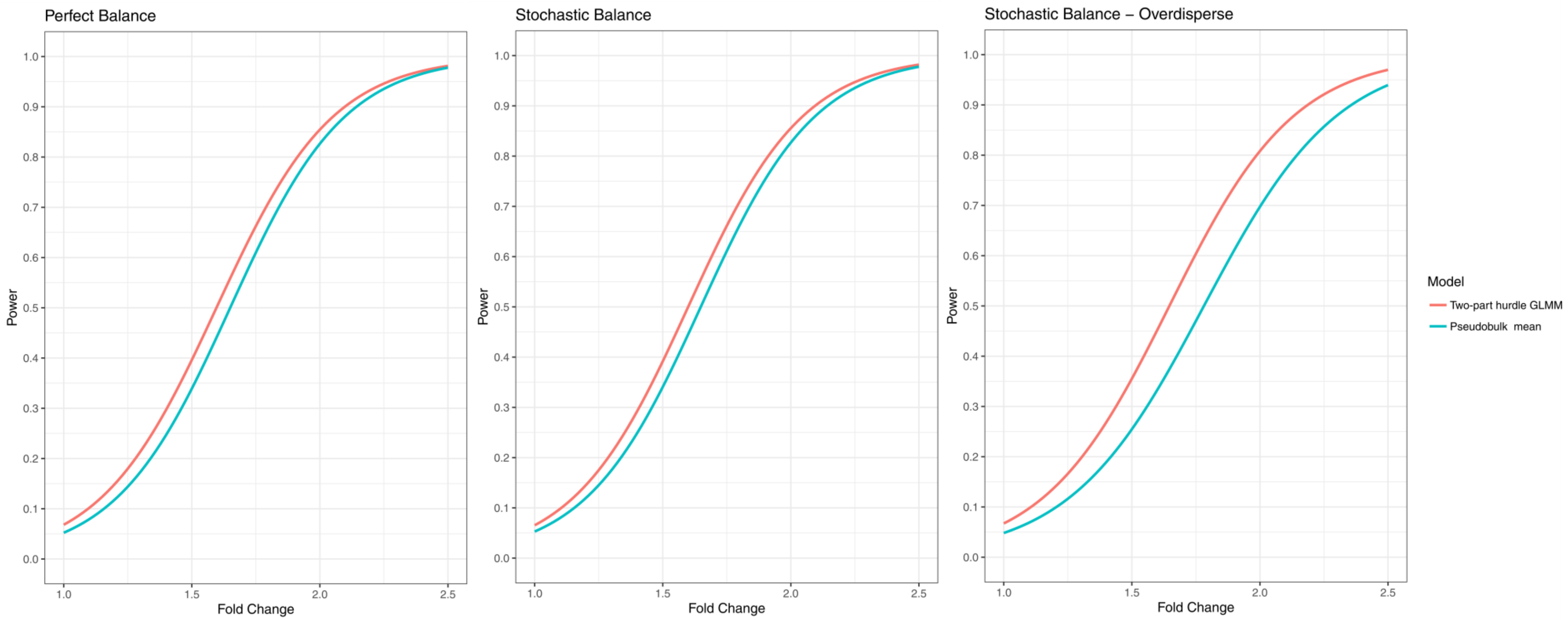

Supplementary Fig. 6: Power comparisons between the two-hurdle model with a random effect for individual and the pseudo-bulk mean approach. Power curves for a two-hurdle model with a random effect for individual (red lines) and a pseudo-bulk mean approach (blue lines) when there are 20 individuals per treatment group. Left: differences in power between the two approaches when each individual has exactly 100 cells. Middle: differences in power between the two approaches when the number of cells for each individual is drawn from a Poisson distribution with an expected value of 100 cells. Right: Differences in power between the two approaches when the number of cells for each individual is drawn from a negative binomial distribution with an expected value of 100 cells and a dispersion parameter equal to one.

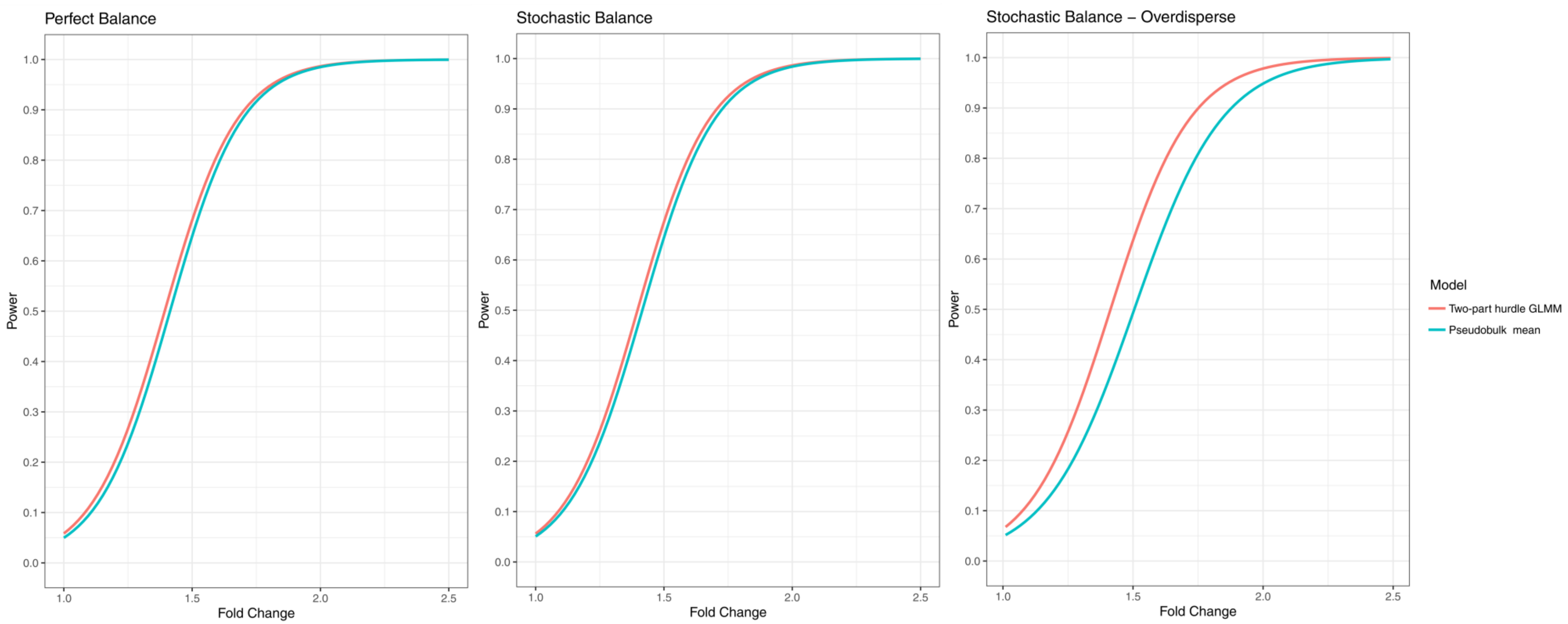

Supplementary Fig. 7: Power comparisons between the two-hurdle model with a random effect for individual and the pseudo-bulk mean approach. Power curves for a two-hurdle model with a random effect for individual (red lines) and a pseudo-bulk mean approach (blue lines) when there are 40 individuals per treatment group. Left panel: Differences in power between the two approaches when each individual has exactly 100 cells. Middle: Differences in power between the two approaches when the number of cells for each individual is drawn from a Poisson distribution with an expected value of 100 cells. Right: Difference in power between the two approaches when the number of cells for each individual is drawn from a negative binomial distribution with an expected value of 100 cells and a dispersion parameter equal to one.

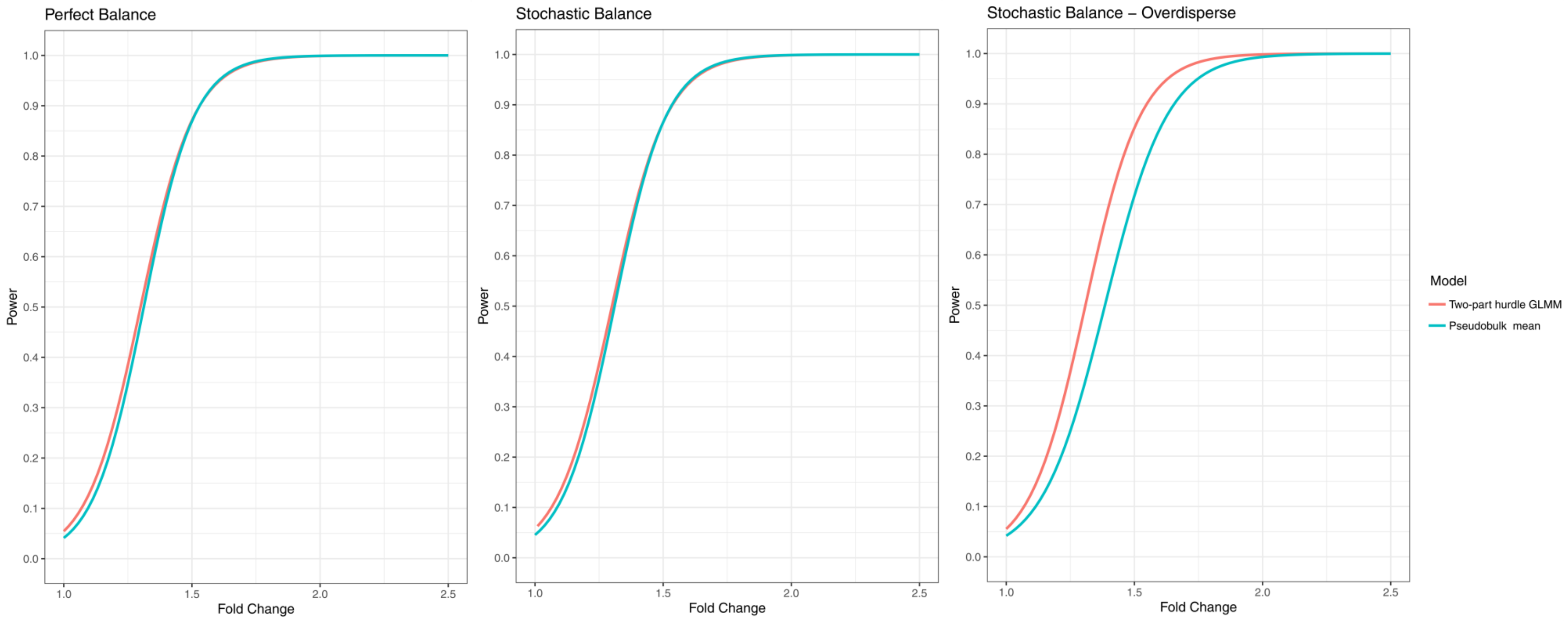

Supplementary Fig. 8: Power comparisons between the two-hurdle model with a random effect for individual and the pseudo-bulk mean approach. Power curves for a two-hurdle model with a random effect for individual (red lines) and a pseudo-bulk mean approach (blue lines) when there are 60 individuals per treatment group. Left panel: Differences in power between the two approaches when each individual has exactly 100 cells. Middle: Differences in power between the two approaches when the number of cells for each individual is drawn from a Poisson distribution with an expected value of 100 cells. Right: Difference in power between the two approaches when the number of cells for each individual is drawn from a negative binomial distribution with an expected value of 100 cells and a dispersion parameter equal to one.

Supplementary Fig. 9: Power calculations using MAST with a random effect for the individual. Power curves for MAST using a random effect to account for intra-individual correlation. Curves are computed for 100 (red line), 250, Blue line) 500 (purple line), and 1,000 (red line) cells per individual with $\alpha = 0.05$. The numbers of individuals per group range from 3 to 100 and are listed above each plot.

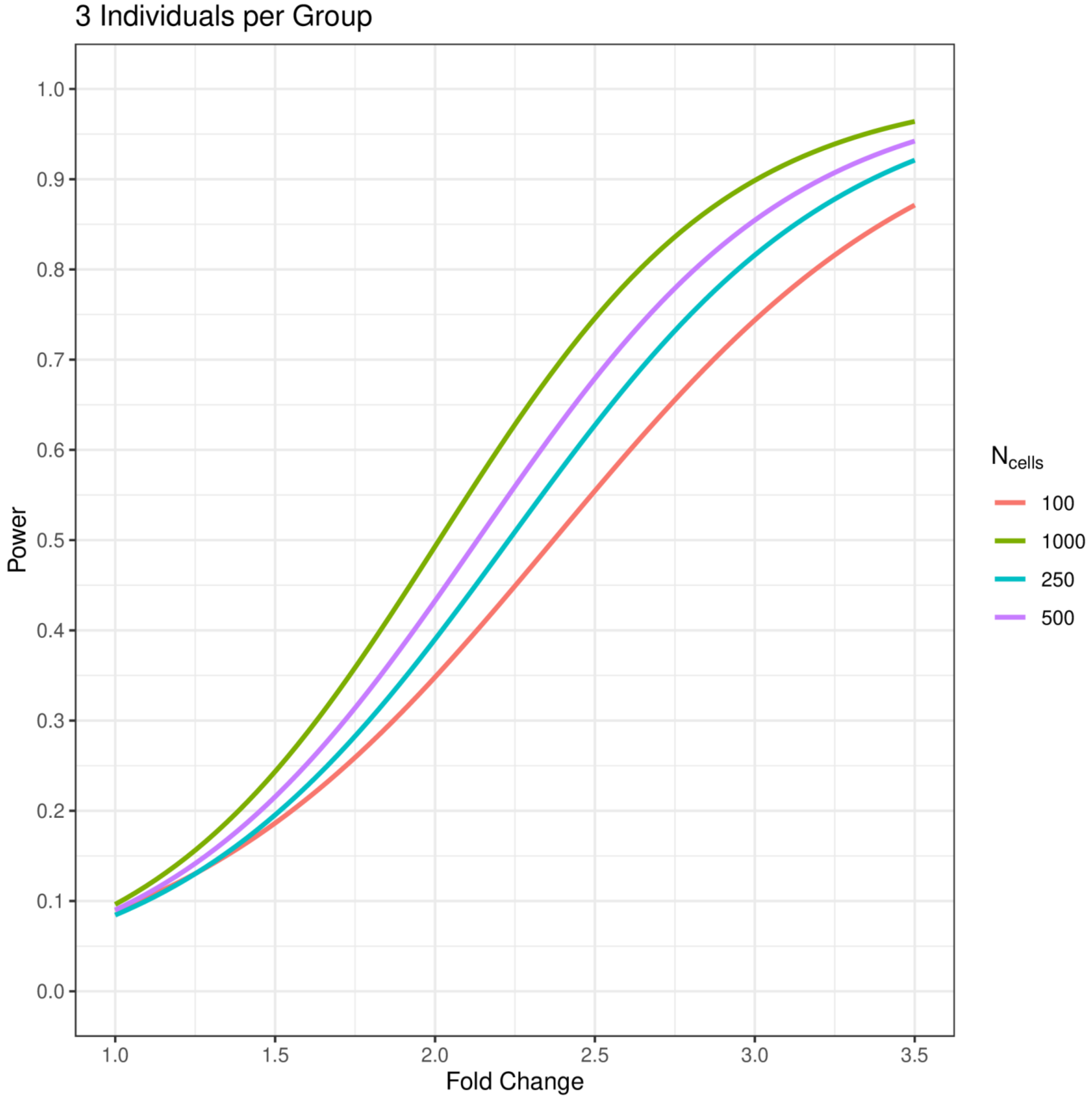

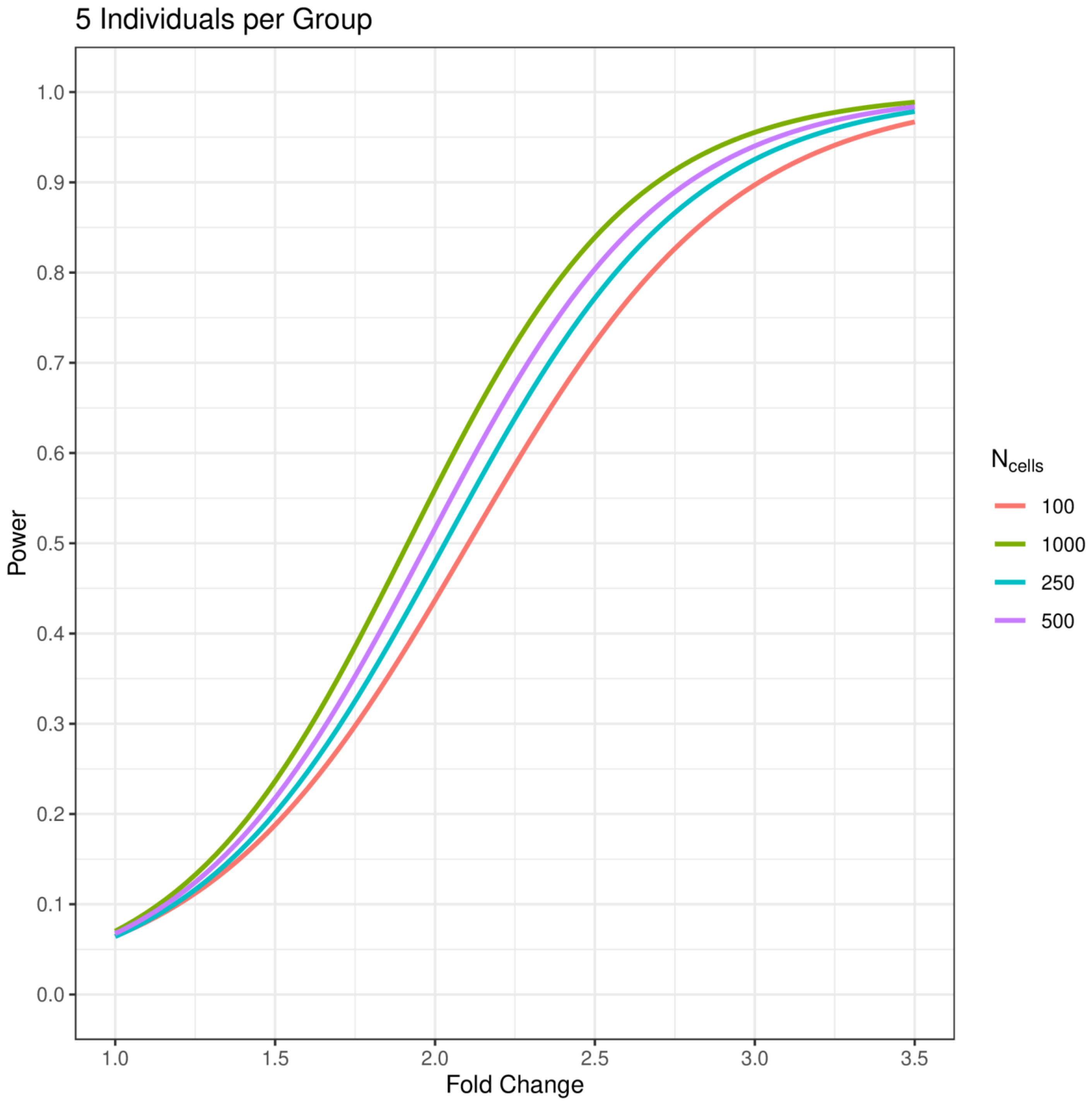

5 Individuals per Group
Power
1.0
0.9
0.8
0.7
0.6
0.5
0.4
0.3
0.2
0.1
0.0
1.0
1.5
2.0
2.5
3.0
3.5
Fold Change
N_{cells}
100
1000
250
500

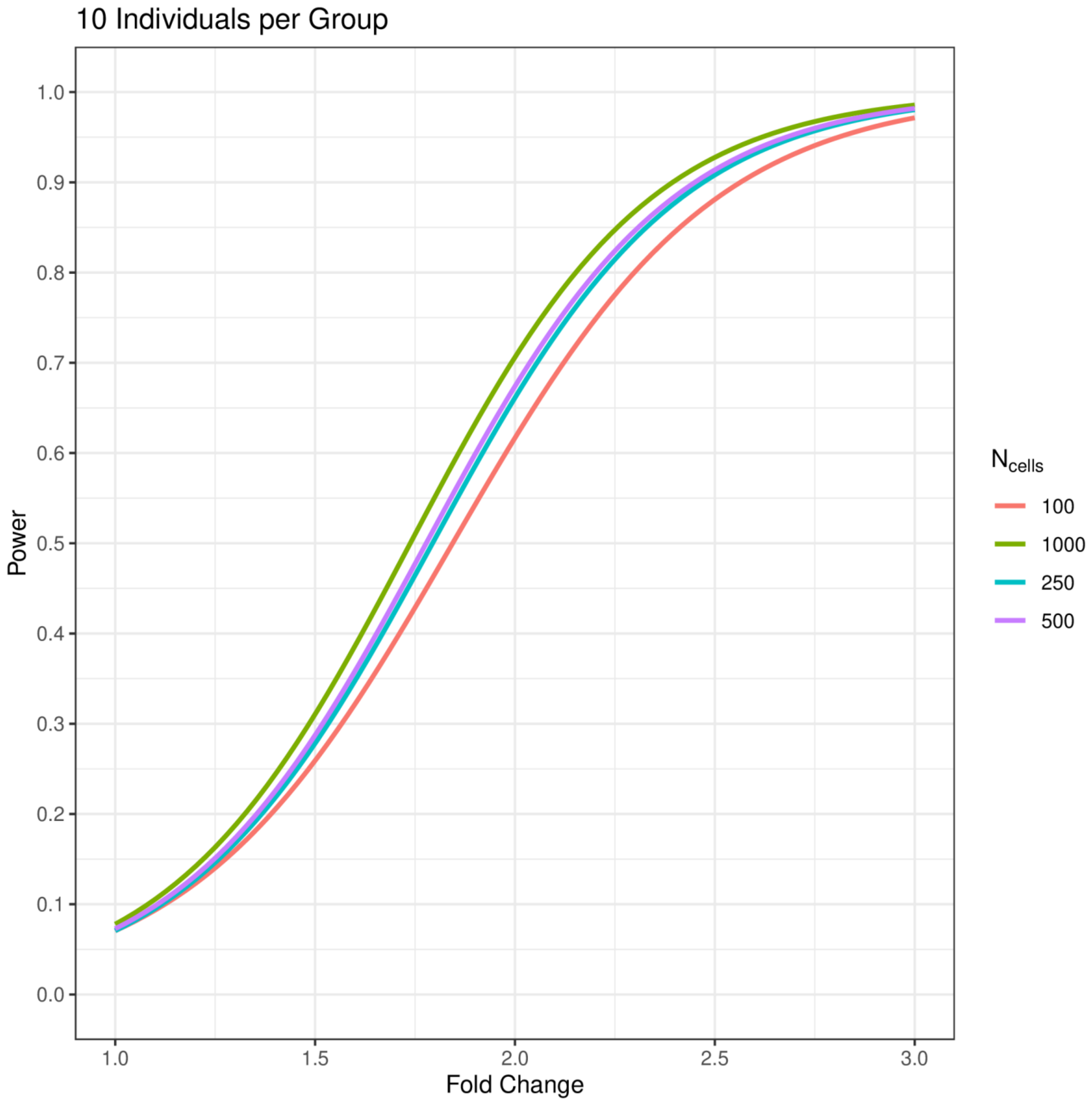

10 Individuals per Group
Power
1.0
0.9
0.8
0.7
0.6
0.5
0.4
0.3
0.2
0.1
0.0
1.0
1.5
2.0
2.5
3.0
Fold Change
N_{cells}
100
1000
250
500

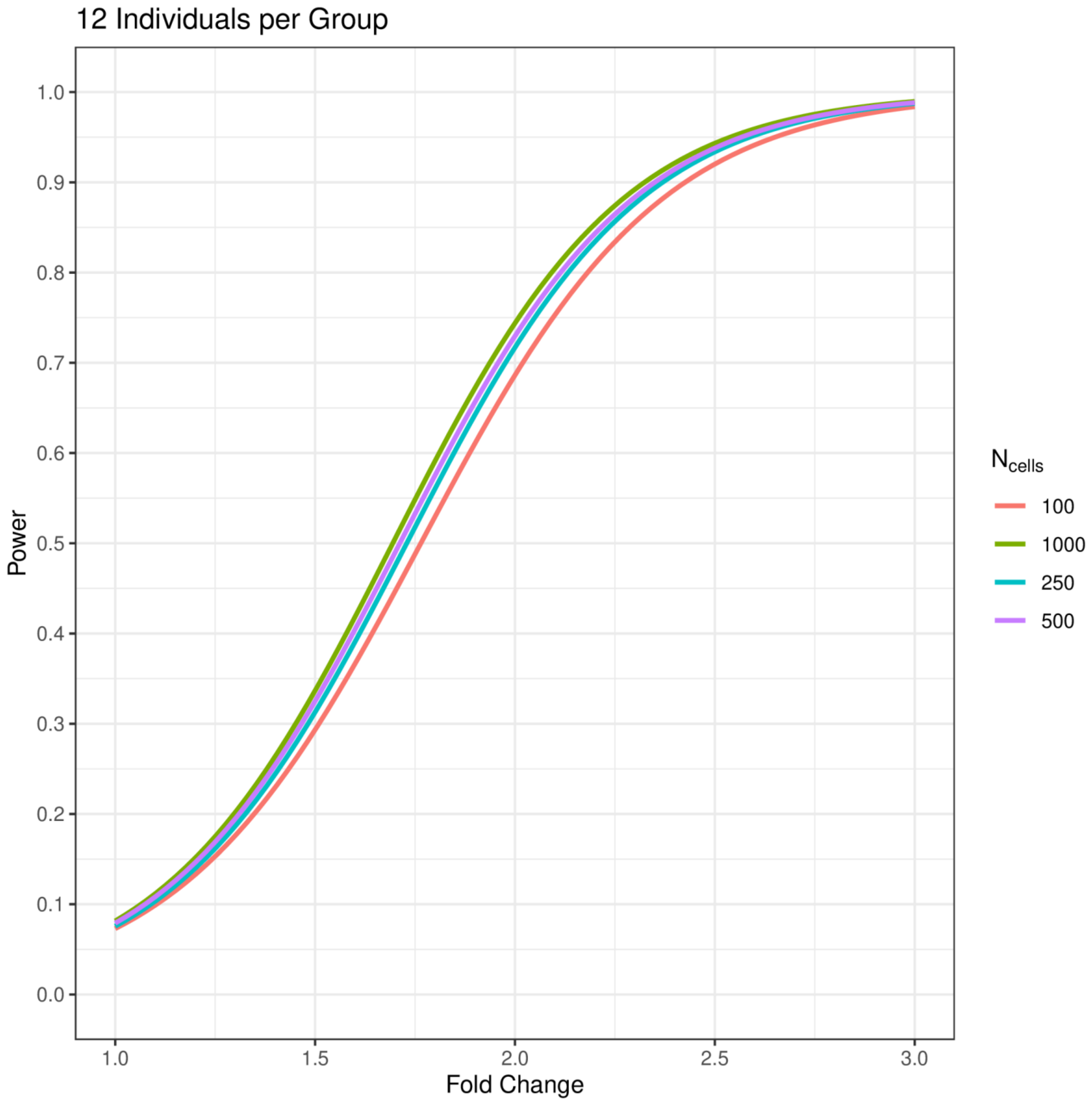

12 Individuals per Group
Power
1.0
0.9
0.8
0.7
0.6
0.5
0.4
0.3
0.2
0.1
0.0
1.0
1.5
2.0
2.5
3.0
Fold Change
N_{cells}
100
1000
250
500

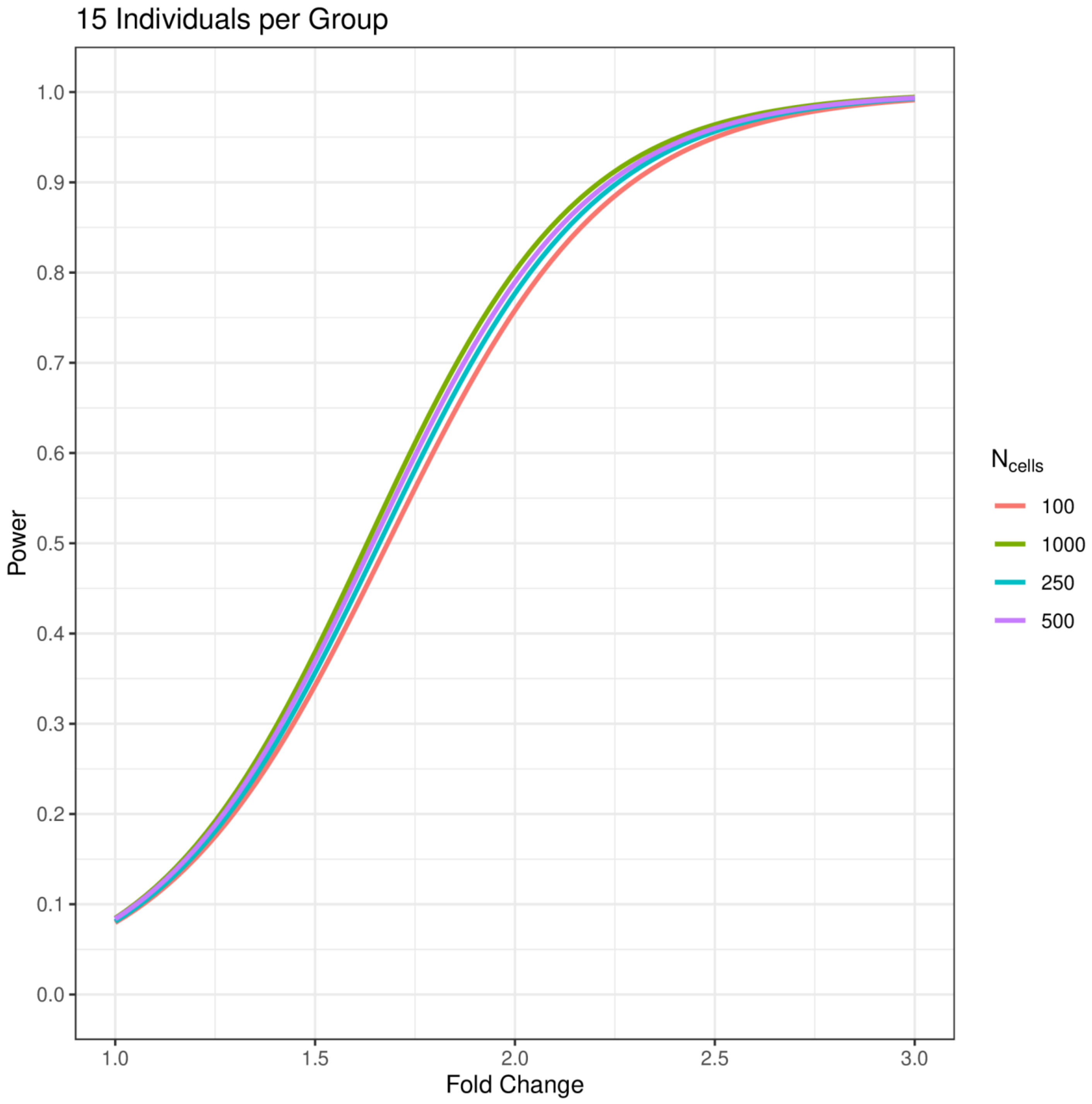
15 Individuals per Group
Power
1.0
0.9
0.8
0.7
0.6
0.5
0.4
0.3
0.2
0.1
0.0
1.0
1.5
2.0
2.5
3.0
Fold Change
Ncells
100
1000
250
500

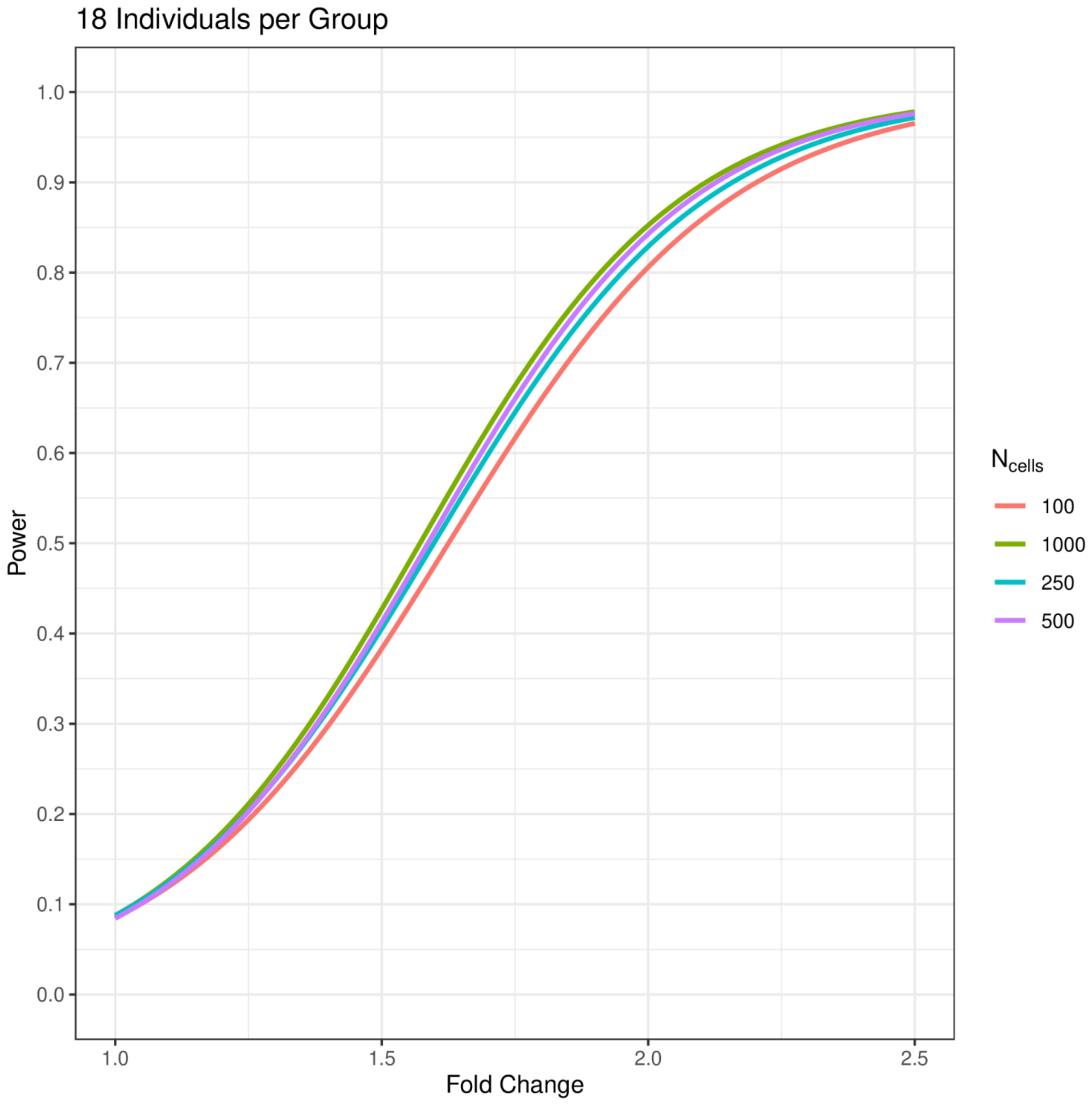
18 Individuals per Group
Power
1.0
0.9
0.8
0.7
0.6
0.5
0.4
0.3
0.2
0.1
0.0
1.0
1.5
2.0
2.5
Fold Change
N_{cells}
100
1000
250
500

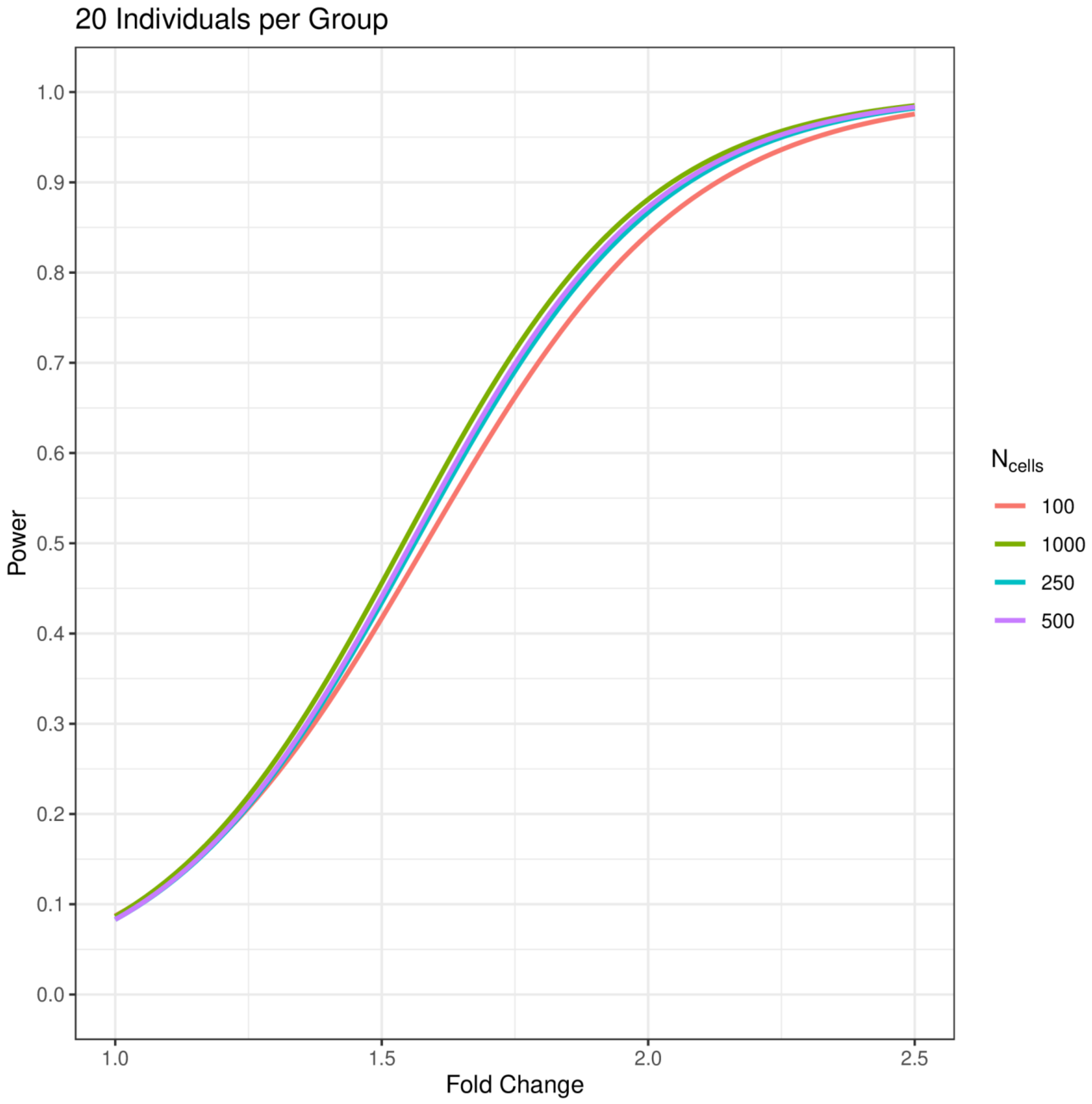
20 Individuals per Group
Power
Fold Change
1.0
0.9
0.8
0.7
0.6
0.5
0.4
0.3
0.2
0.1
0.0
1.0
1.5
2.0
2.5
N_{cells}
100
1000
250
500

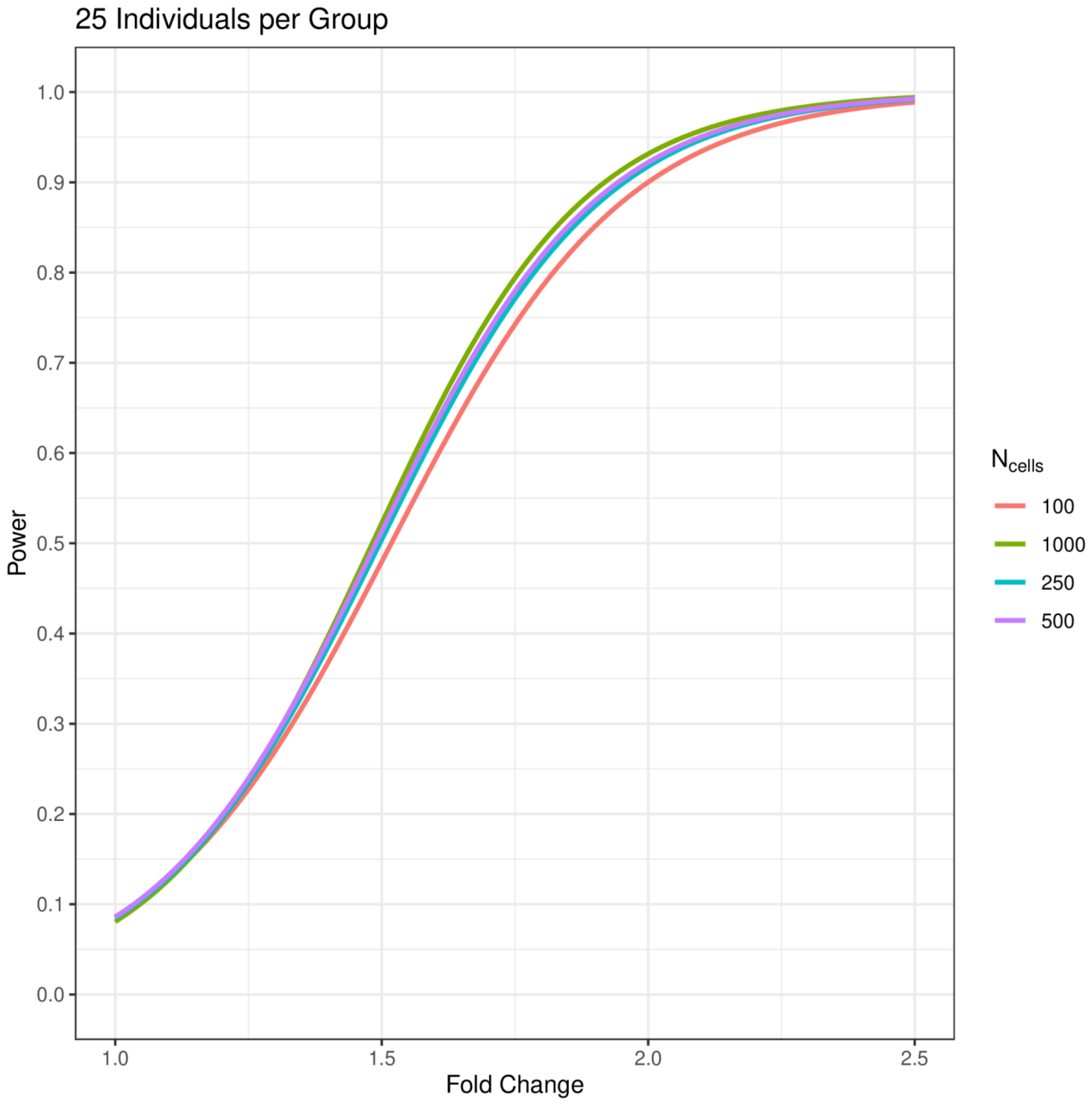
25 Individuals per Group
1.0
0.9
0.8
0.7
0.6
0.5
0.4
0.3
0.2
0.1
0.0
Power
1.0
1.5
2.0
2.5
Fold Change
N cells
100
1000
250
500

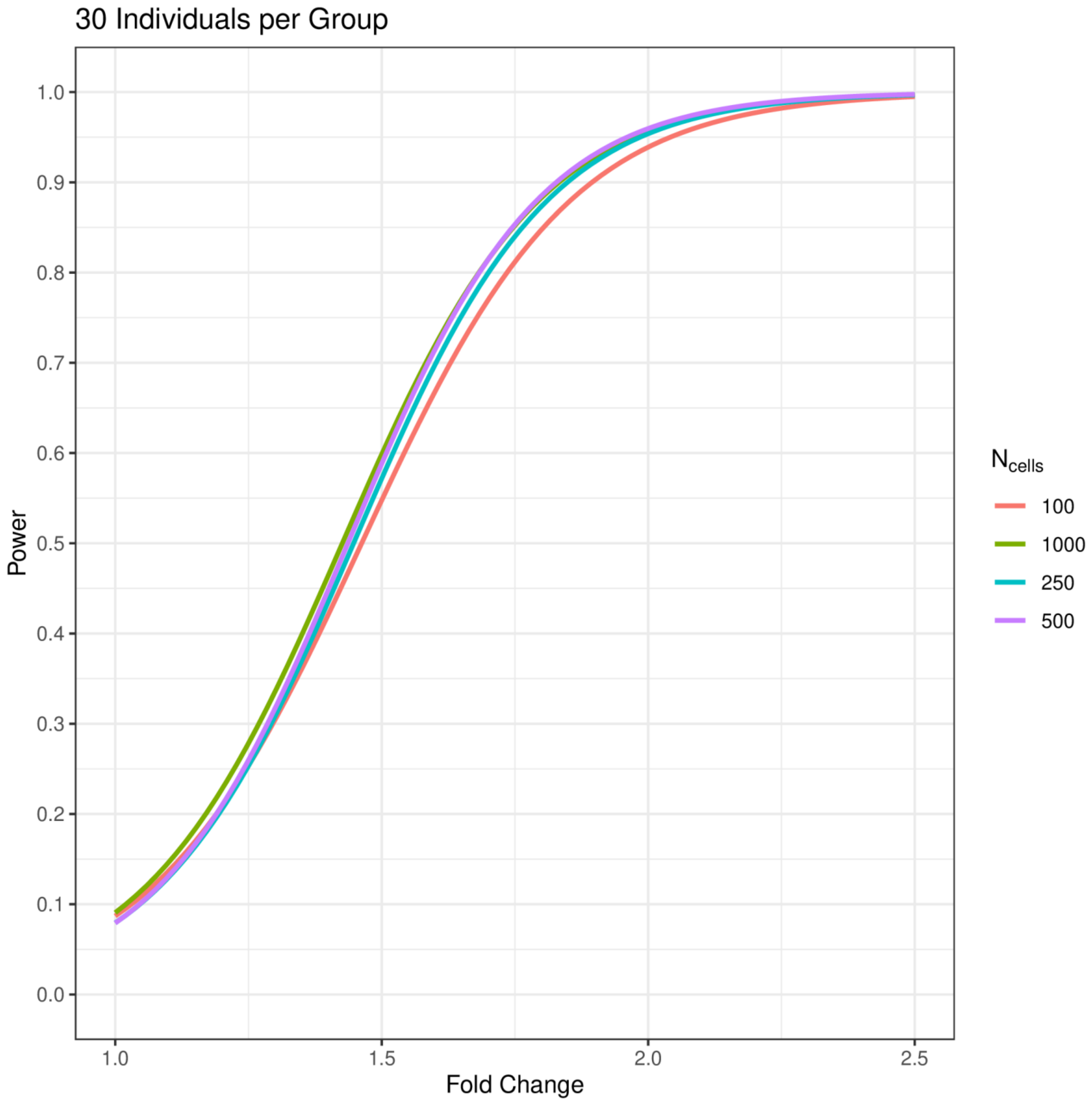
30 Individuals per Group
1.0
0.9
0.8
0.7
0.6
0.5
0.4
0.3
0.2
0.1
0.0
Power
1.0
1.5
2.0
2.5
Fold Change
N cells
100
1000
250
500

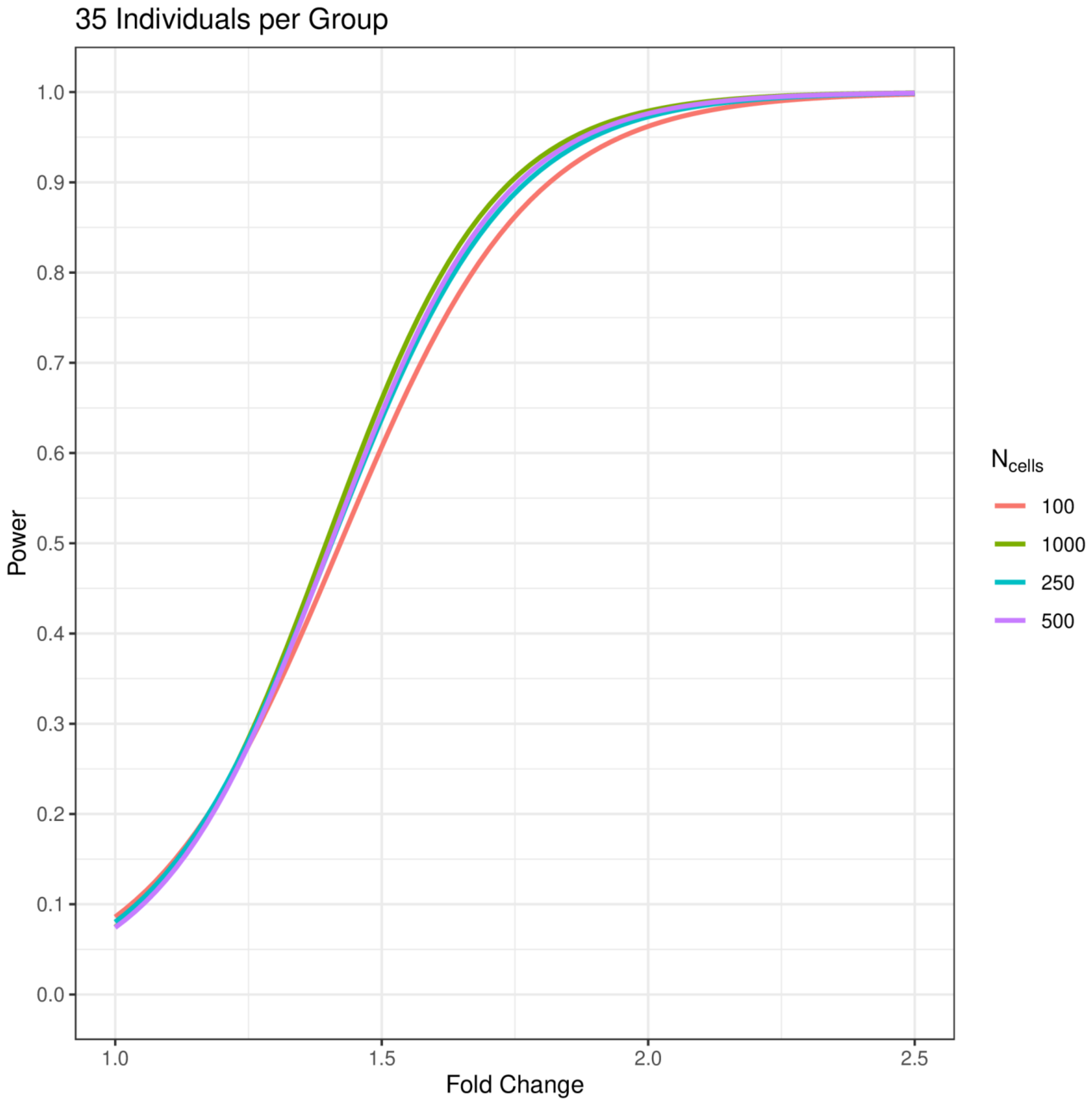
35 Individuals per Group
Power
1.0
0.9
0.8
0.7
0.6
0.5
0.4
0.3
0.2
0.1
0.0
1.0
1.5
2.0
2.5
Fold Change
N_{cells}
100
1000
250
500

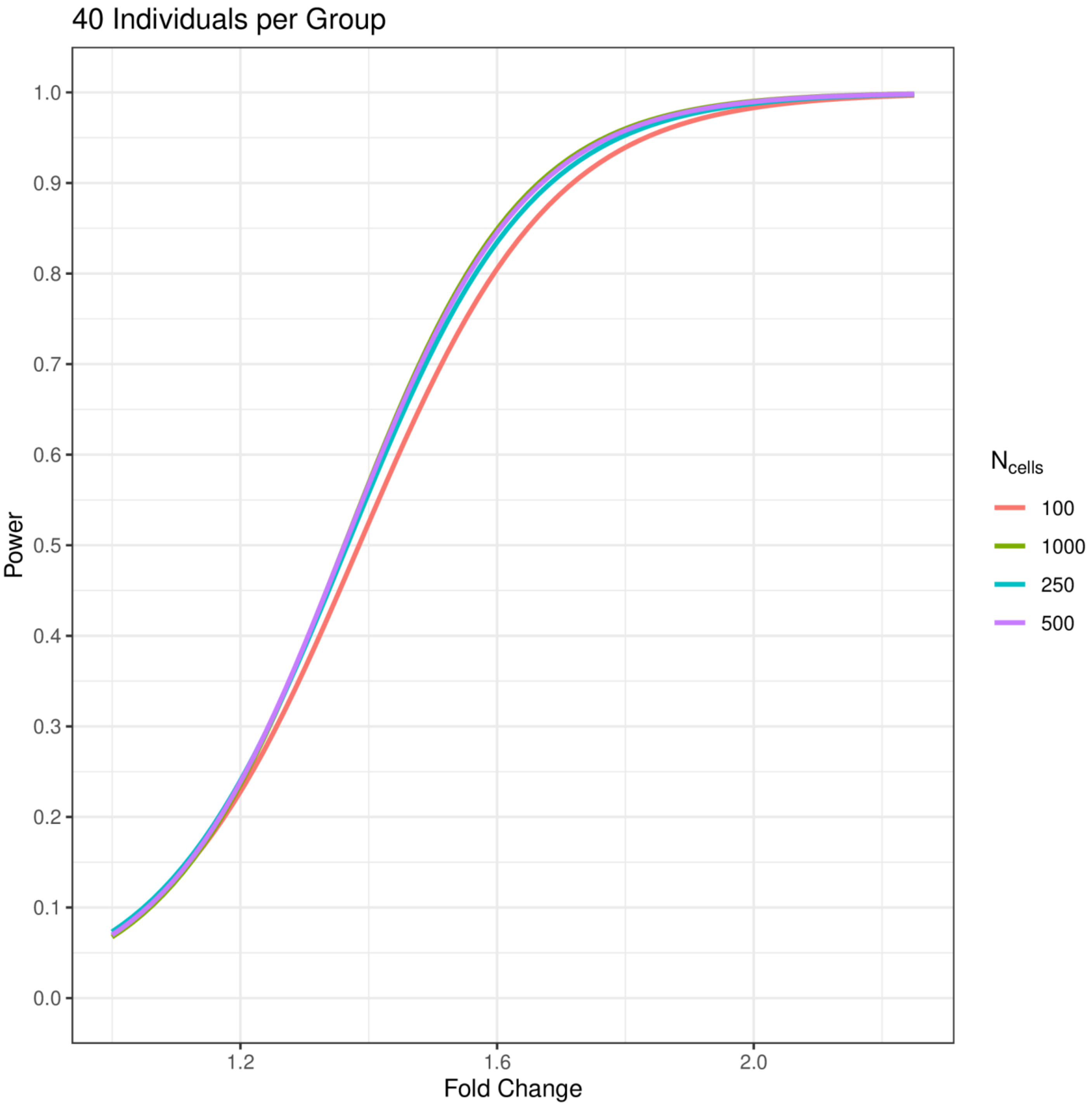
40 Individuals per Group
1.0
0.9
0.8
0.7
0.6
0.5
0.4
0.3
0.2
0.1
0.0
Power
1.2
1.6
2.0
Fold Change
N_{cells}
100
1000
250
500

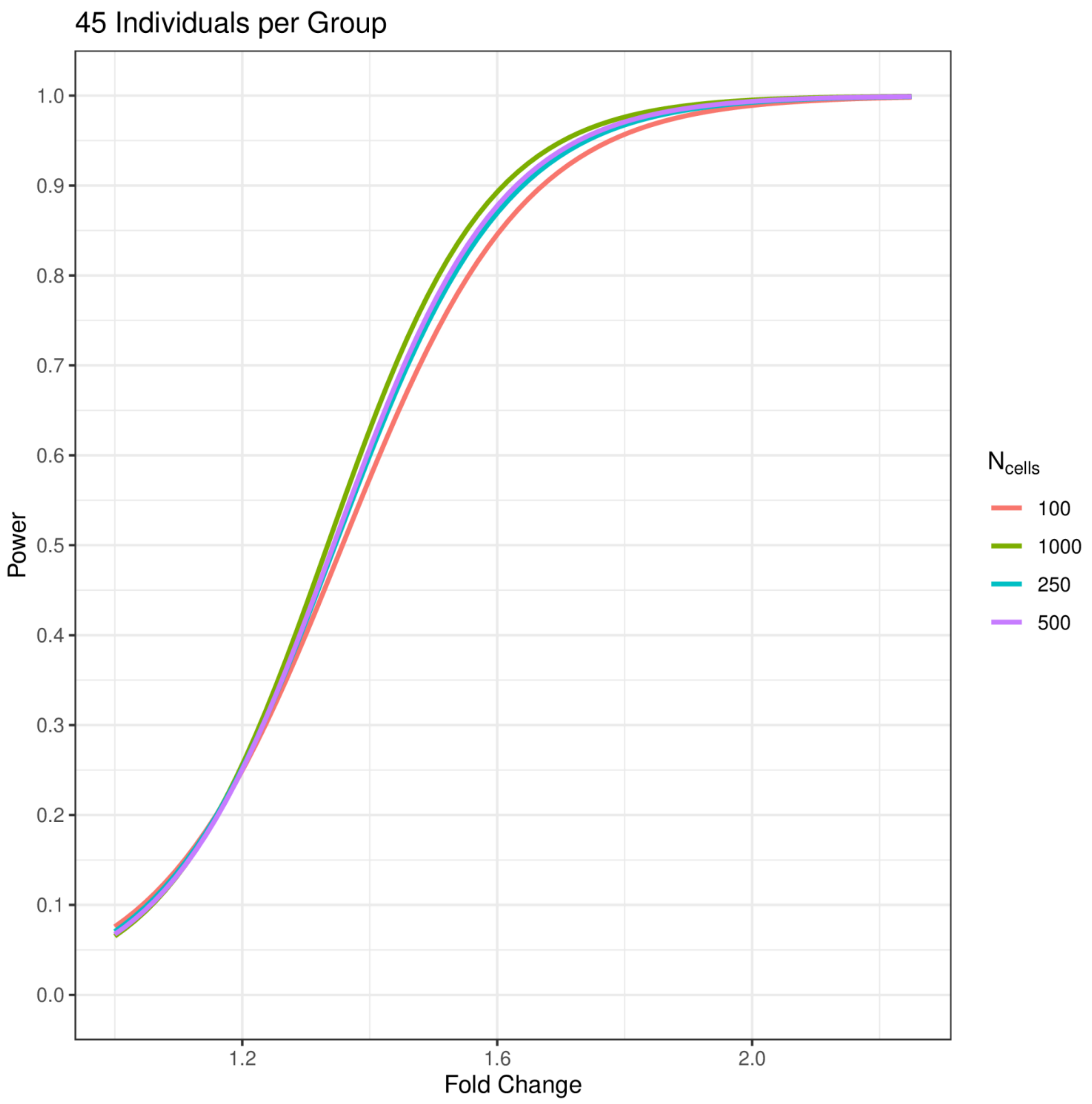
45 Individuals per Group
Power
1.0
0.9
0.8
0.7
0.6
0.5
0.4
0.3
0.2
0.1
0.0
1.2
1.6
2.0
Fold Change
N_{cells}
100
1000
250
500

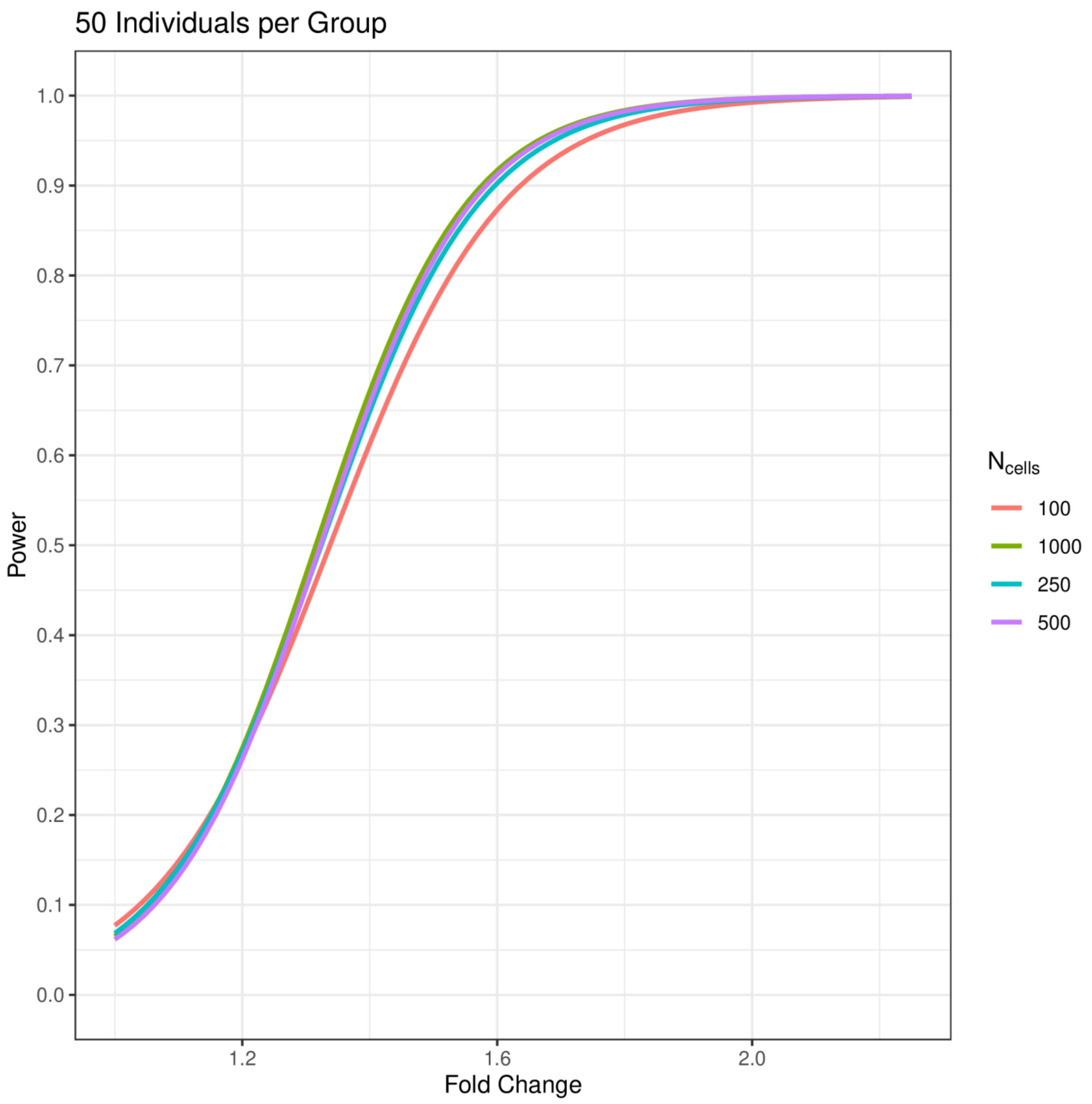
50 Individuals per Group
Power
1.0
0.9
0.8
0.7
0.6
0.5
0.4
0.3
0.2
0.1
0.0
1.2
1.6
2.0
Fold Change
N_{cells}
100
1000
250
500

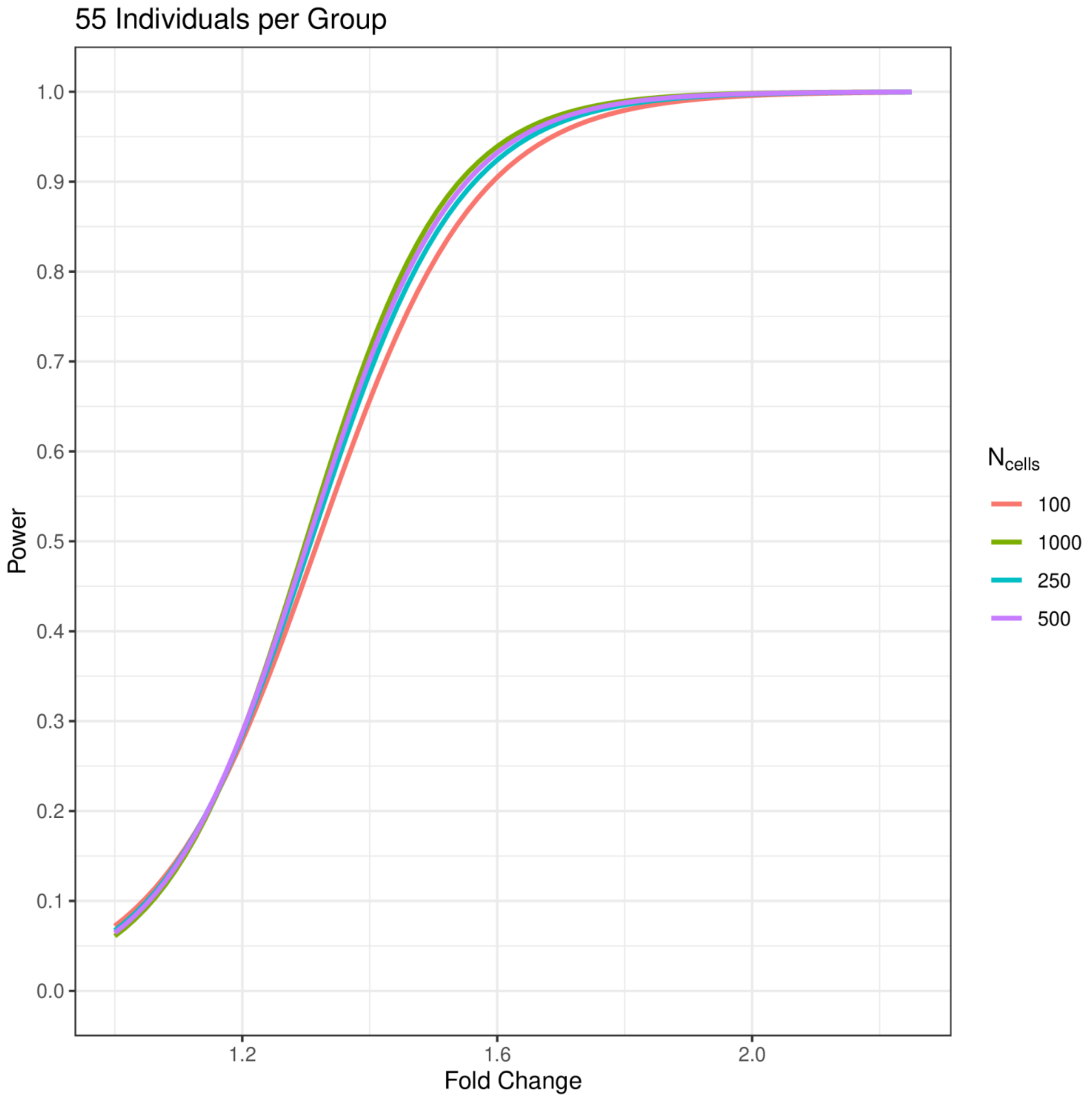
55 Individuals per Group
Power
1.0
0.9
0.8
0.7
0.6
0.5
0.4
0.3
0.2
0.1
0.0
1.2
1.6
2.0
Fold Change
N_{cells}
100
1000
250
500

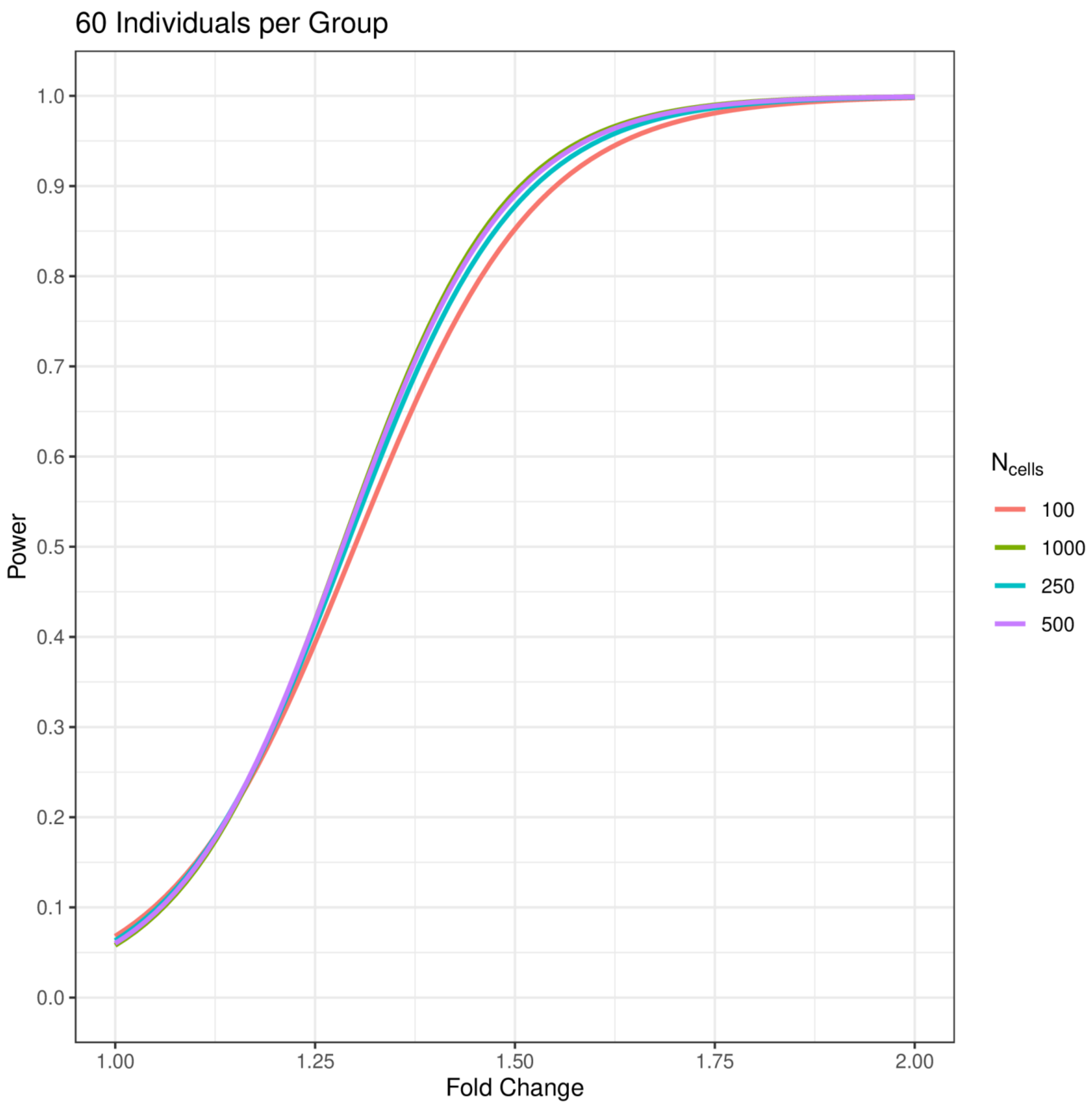
60 Individuals per Group
Power
1.0
0.9
0.8
0.7
0.6
0.5
0.4
0.3
0.2
0.1
0.0
1.00
1.25
1.50
1.75
2.00
Fold Change
N_{cells}
100
1000
250
500

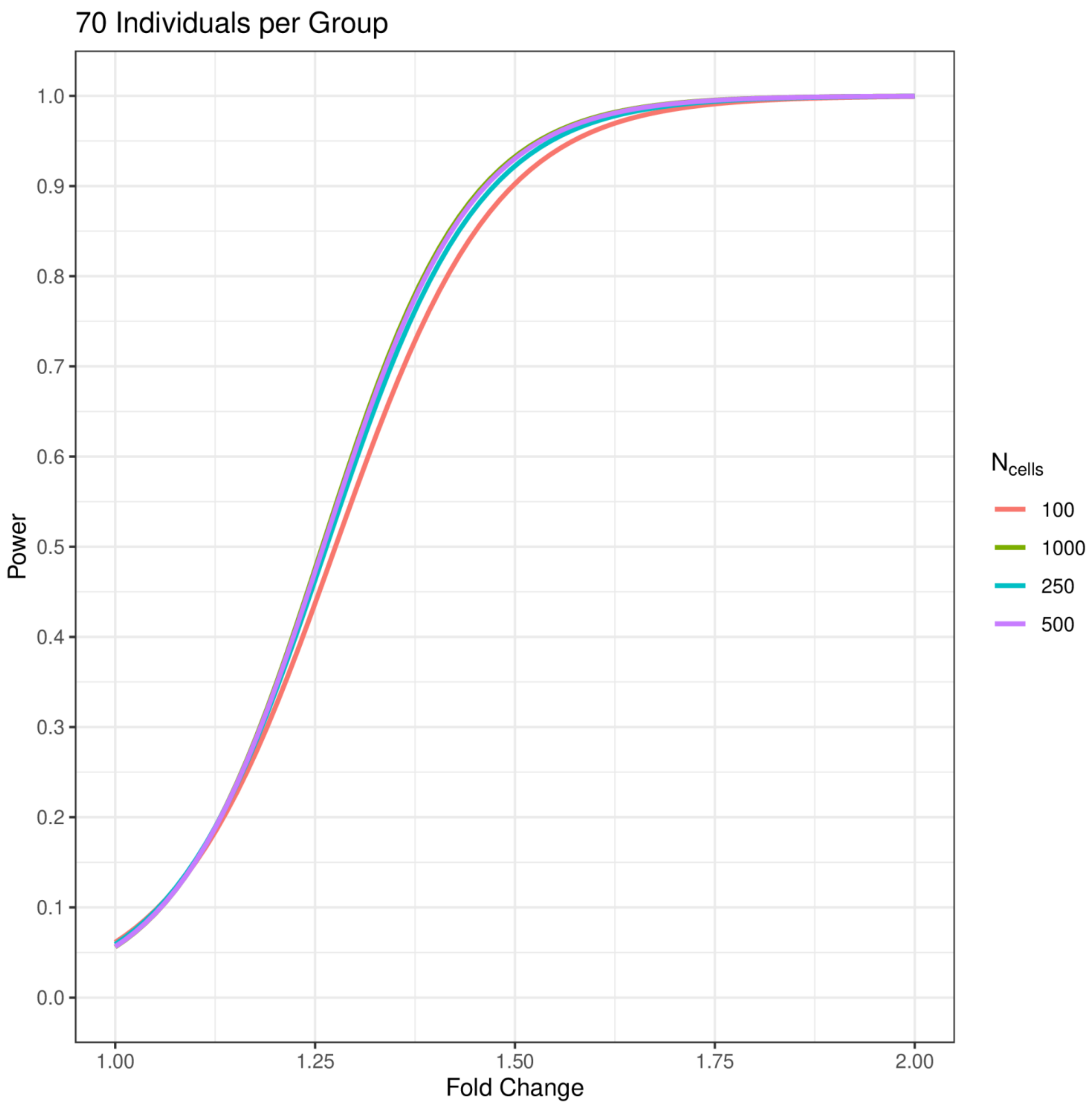
70 Individuals per Group
Power
1.0
0.9
0.8
0.7
0.6
0.5
0.4
0.3
0.2
0.1
0.0
1.00
1.25
1.50
1.75
2.00
Fold Change
N_{cells}
100
1000
250
500

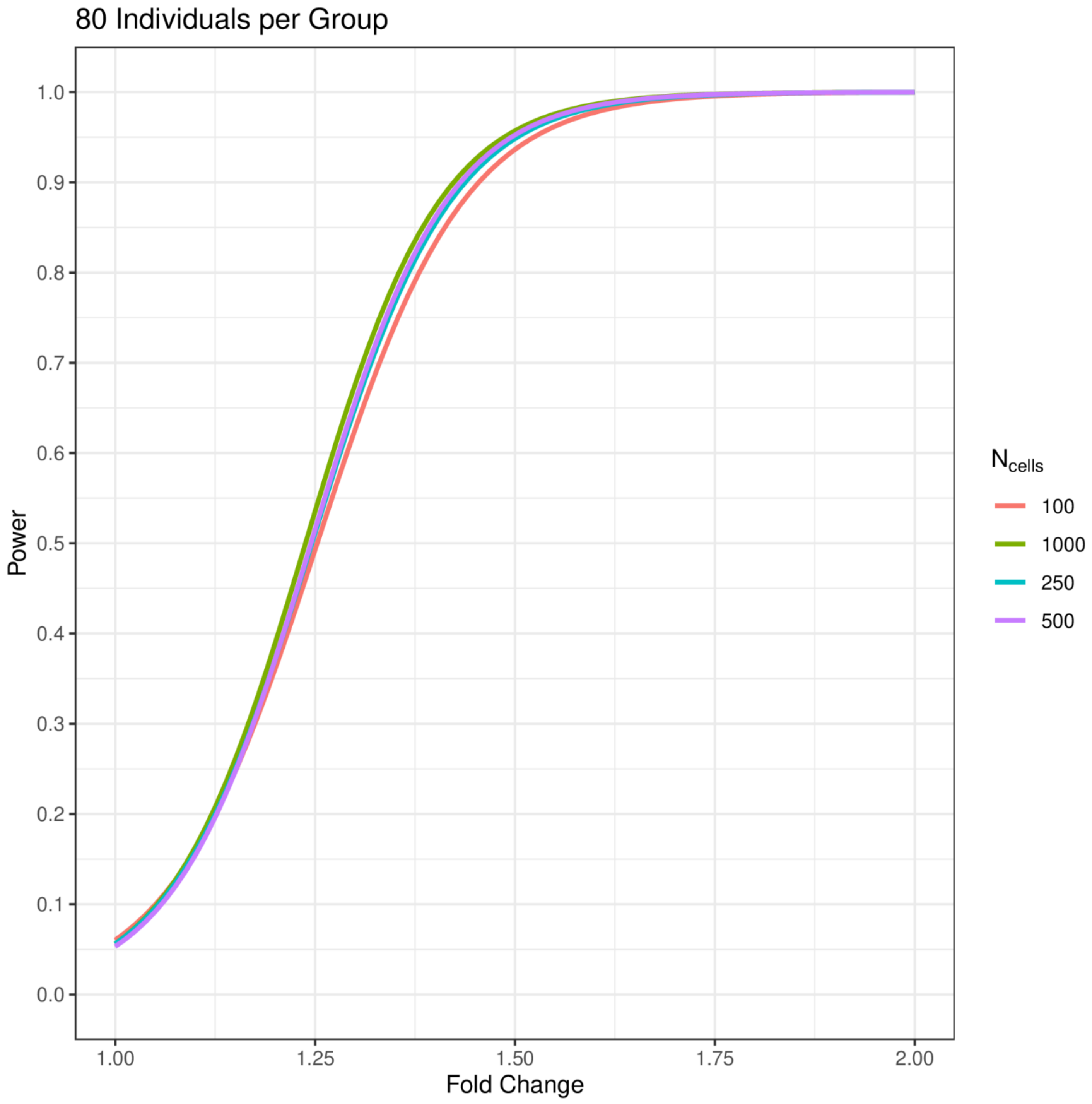
80 Individuals per Group
1.0
0.9
0.8
0.7
0.6
0.5
0.4
0.3
0.2
0.1
0.0
Power
1.00
1.25
1.50
1.75
2.00
Fold Change
N_{cells}
100
1000
250
500

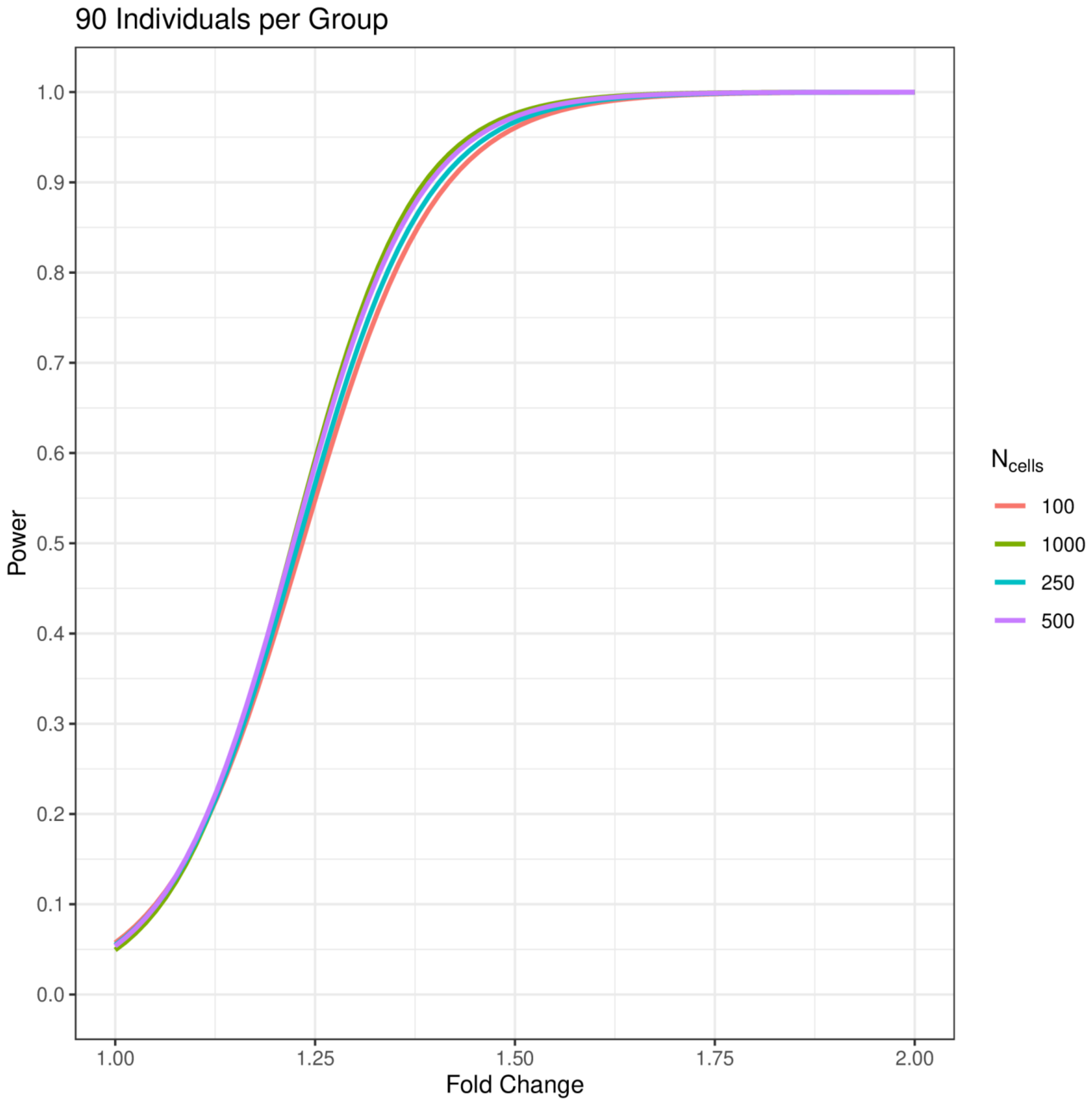
90 Individuals per Group
1.0
0.9
0.8
0.7
0.6
0.5
0.4
0.3
0.2
0.1
0.0
Power
1.00
1.25
1.50
1.75
2.00
Fold Change
N_{cells}
100
1000
250
500

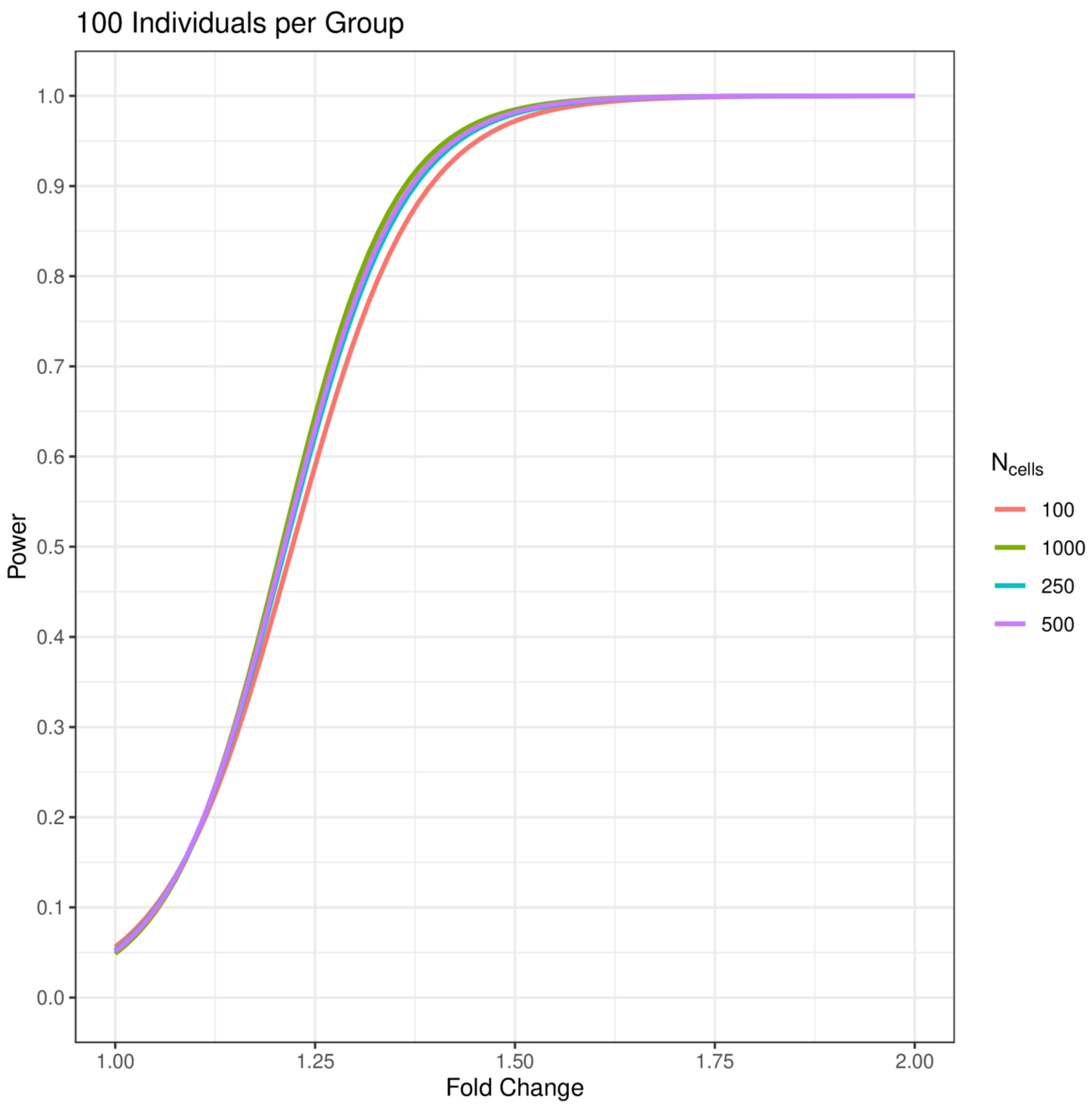
100 Individuals per Group
1.0
0.9
0.8
0.7
0.6
0.5
0.4
0.3
0.2
0.1
0.0
Power
1.00
1.25
1.50
1.75
2.00
Fold Change
N_{cells}
100
1000
250
500

Supplementary Fig. 10: Power calculations using MAST with a random effect for individual. Power curves for MAST using a random effect to account for intra-individual correlation. Curves are computed for 100, 250, 500, and 1,000 cells per individual using an $\alpha = 0.01$. The number of individuals per group ranges from 3 to 100 and is listed above each plot.

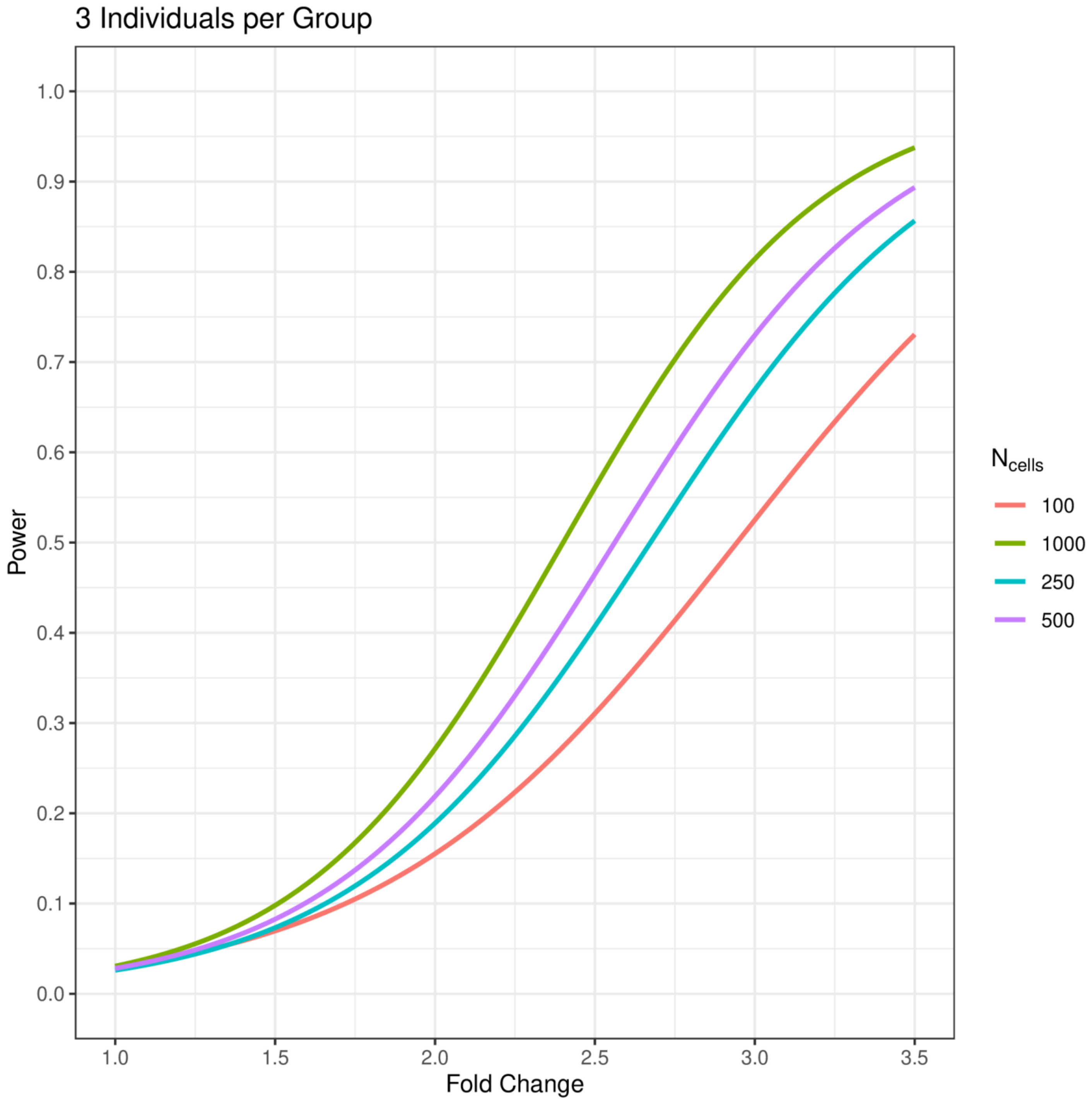

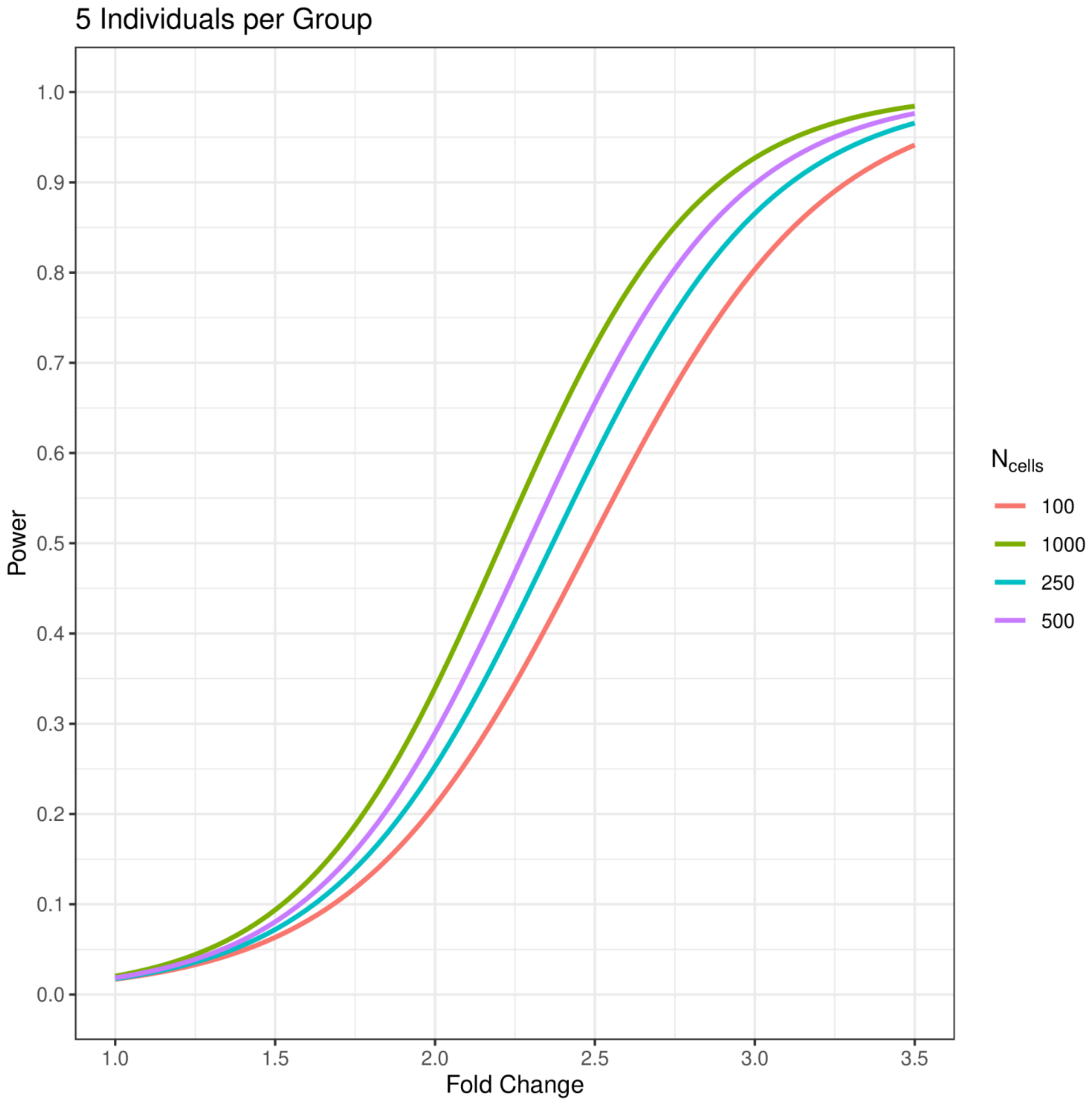
5 Individuals per Group
Power
Fold Change
1.0
0.9
0.8
0.7
0.6
0.5
0.4
0.3
0.2
0.1
0.0
1.0
1.5
2.0
2.5
3.0
3.5
N_{cells}
100
1000
250
500

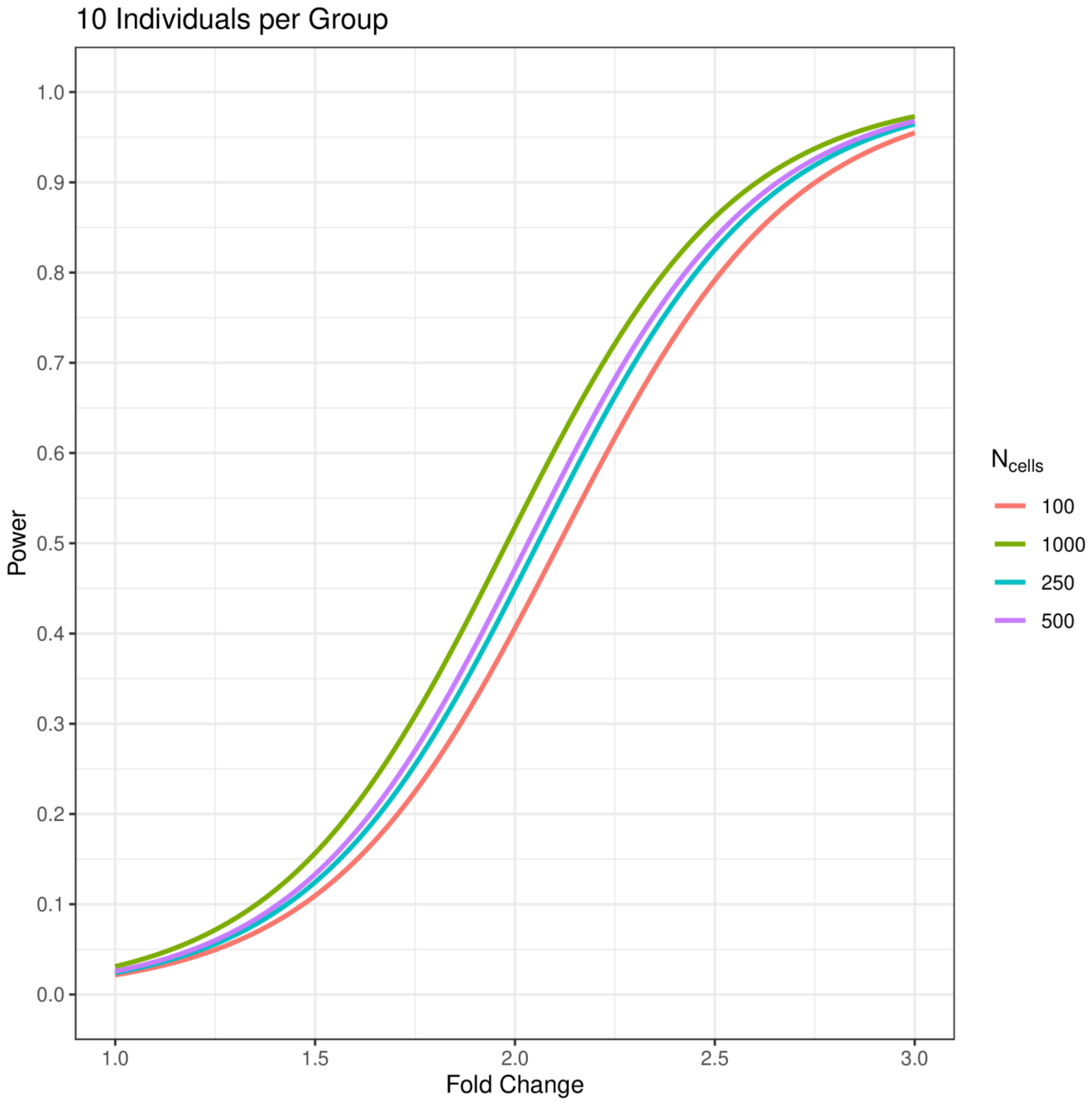
10 Individuals per Group
Power
1.0
0.9
0.8
0.7
0.6
0.5
0.4
0.3
0.2
0.1
0.0
1.0
1.5
2.0
2.5
3.0
Fold Change
N_{cells}
100
1000
250
500

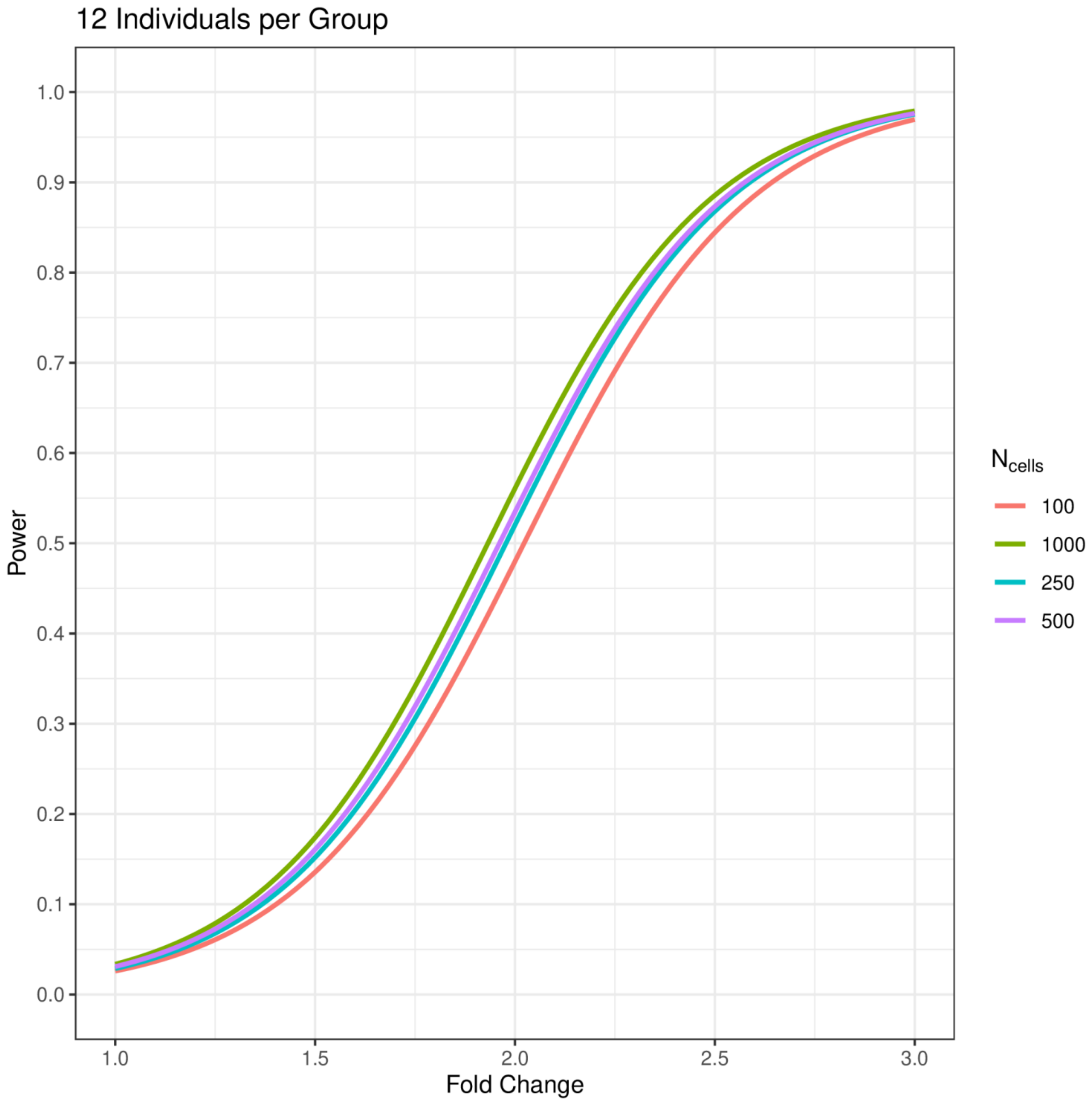
12 Individuals per Group
Power
1.0
0.9
0.8
0.7
0.6
0.5
0.4
0.3
0.2
0.1
0.0
1.0
1.5
2.0
2.5
3.0
Fold Change
N_{cells}
100
1000
250
500

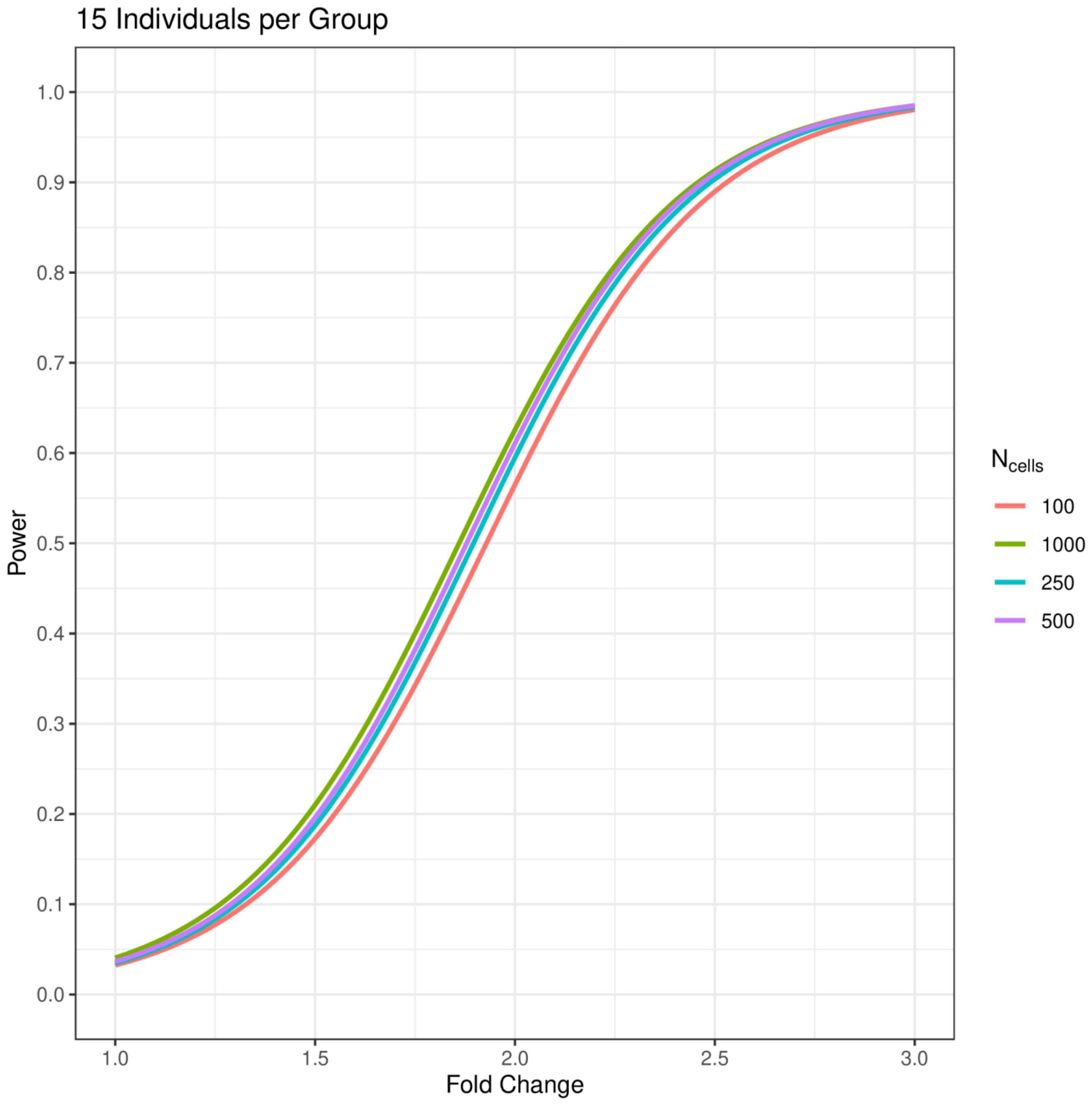
15 Individuals per Group
Power
1.0
0.9
0.8
0.7
0.6
0.5
0.4
0.3
0.2
0.1
0.0
1.0
1.5
2.0
2.5
3.0
Fold Change
N_{cells}
100
1000
250
500

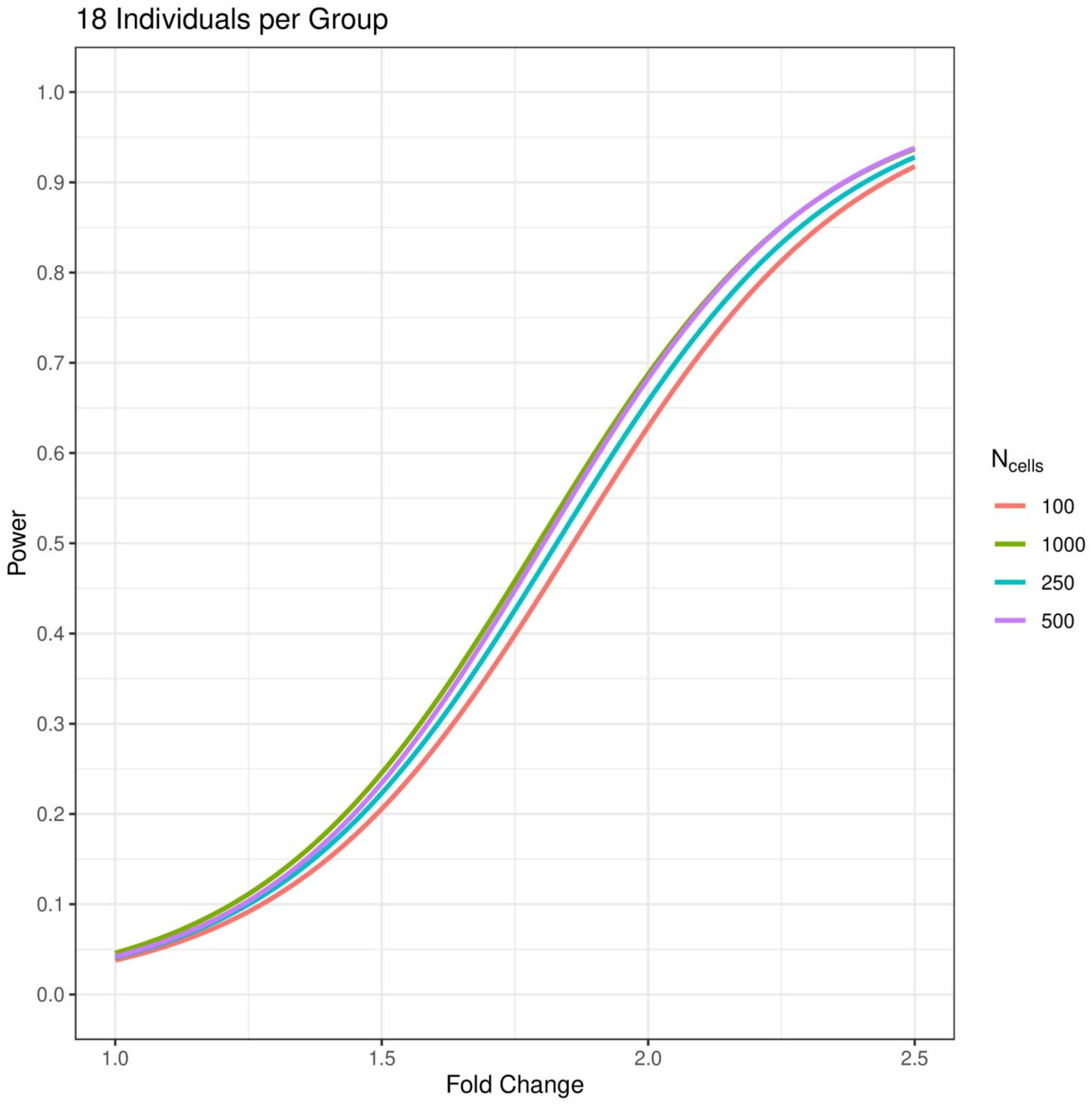
18 Individuals per Group
1.0
0.9
0.8
0.7
0.6
0.5
0.4
0.3
0.2
0.1
0.0
Power
1.0
1.5
2.0
2.5
Fold Change
N_{cells}
100
1000
250
500

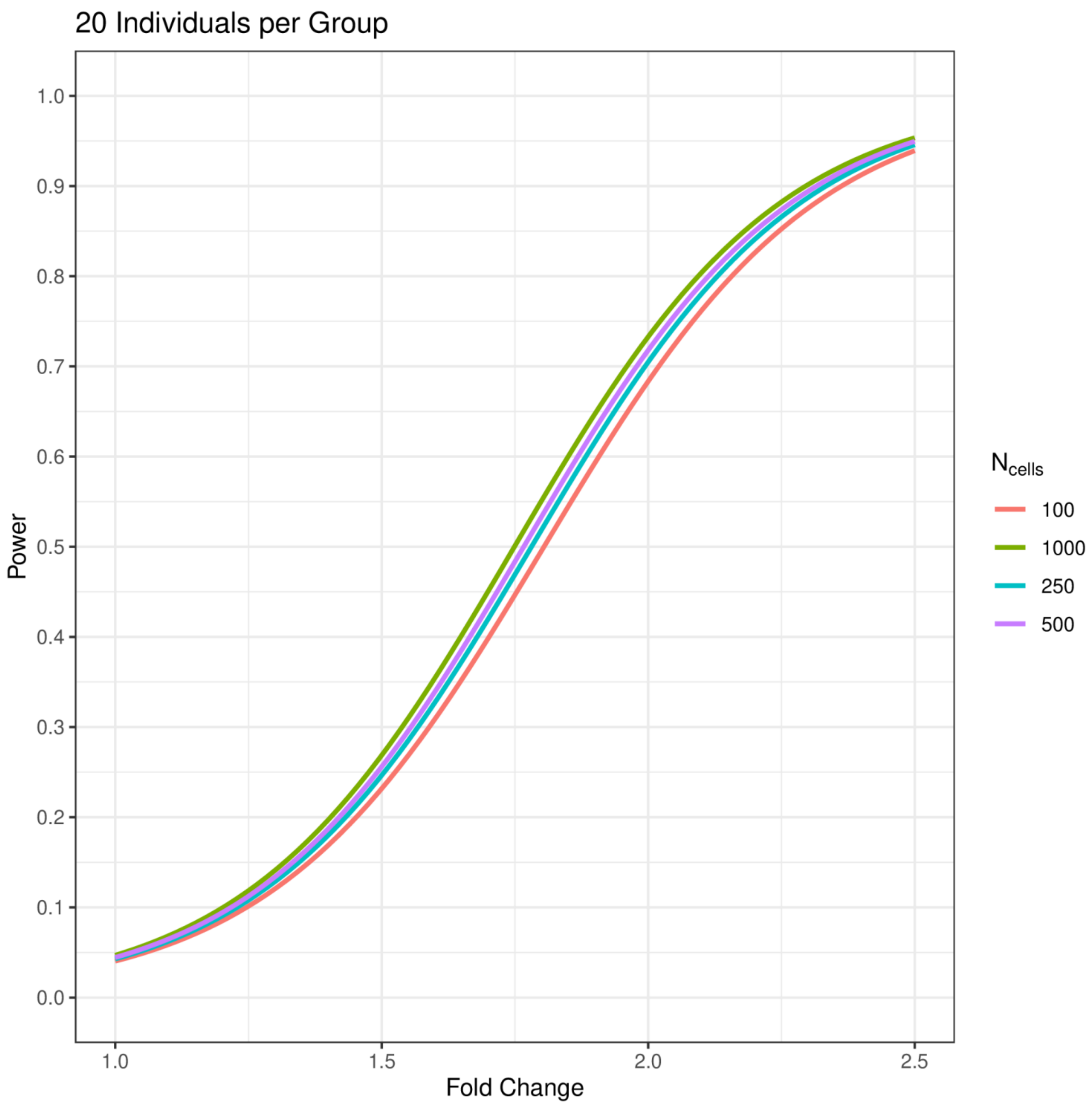
20 Individuals per Group
Power
1.0
0.9
0.8
0.7
0.6
0.5
0.4
0.3
0.2
0.1
0.0
1.0
1.5
2.0
2.5
Fold Change
N_{cells}
100
1000
250
500

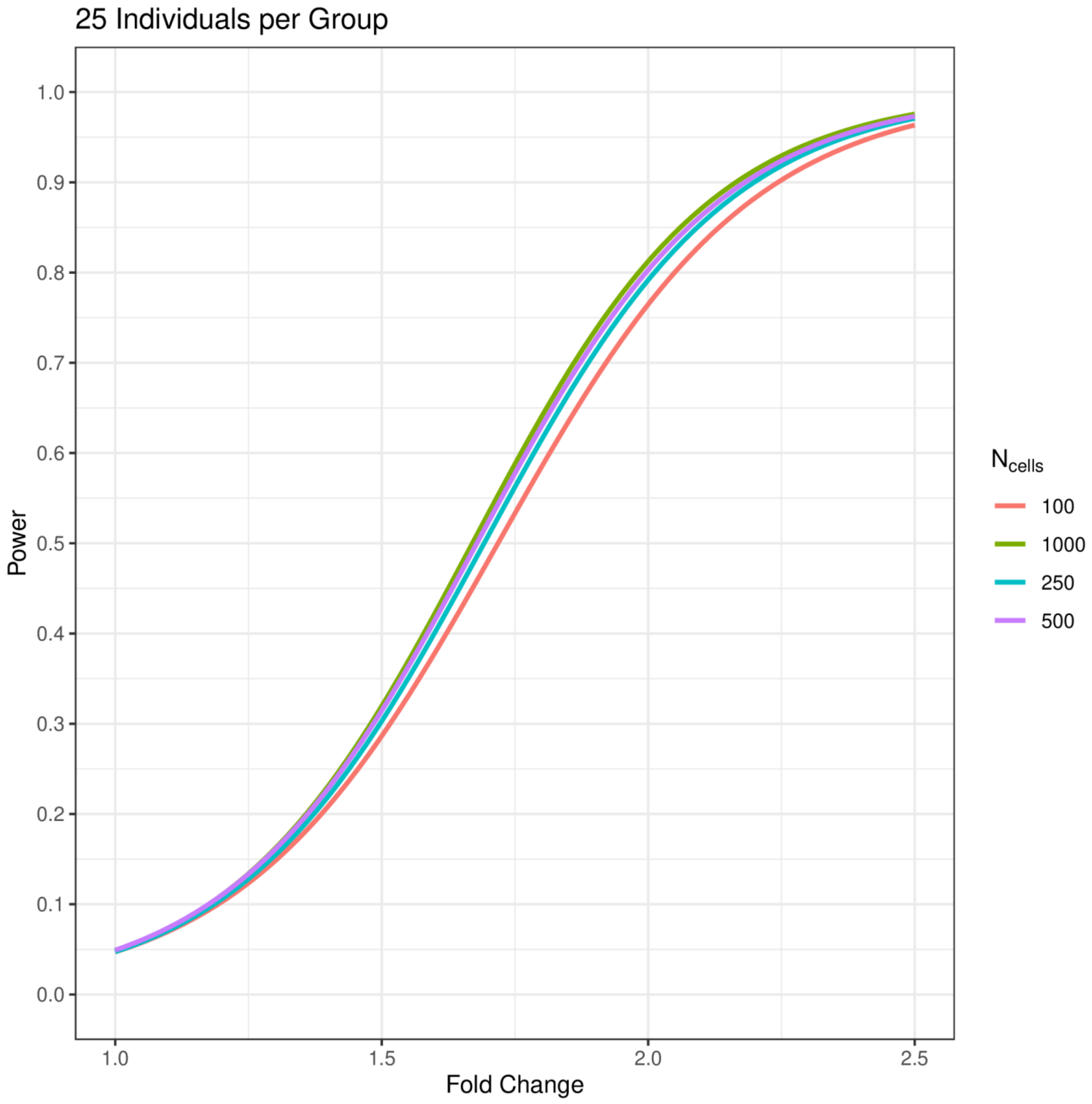
25 Individuals per Group
Power
1.0
0.9
0.8
0.7
0.6
0.5
0.4
0.3
0.2
0.1
0.0
1.0
1.5
2.0
2.5
Fold Change
N cells
100
1000
250
500

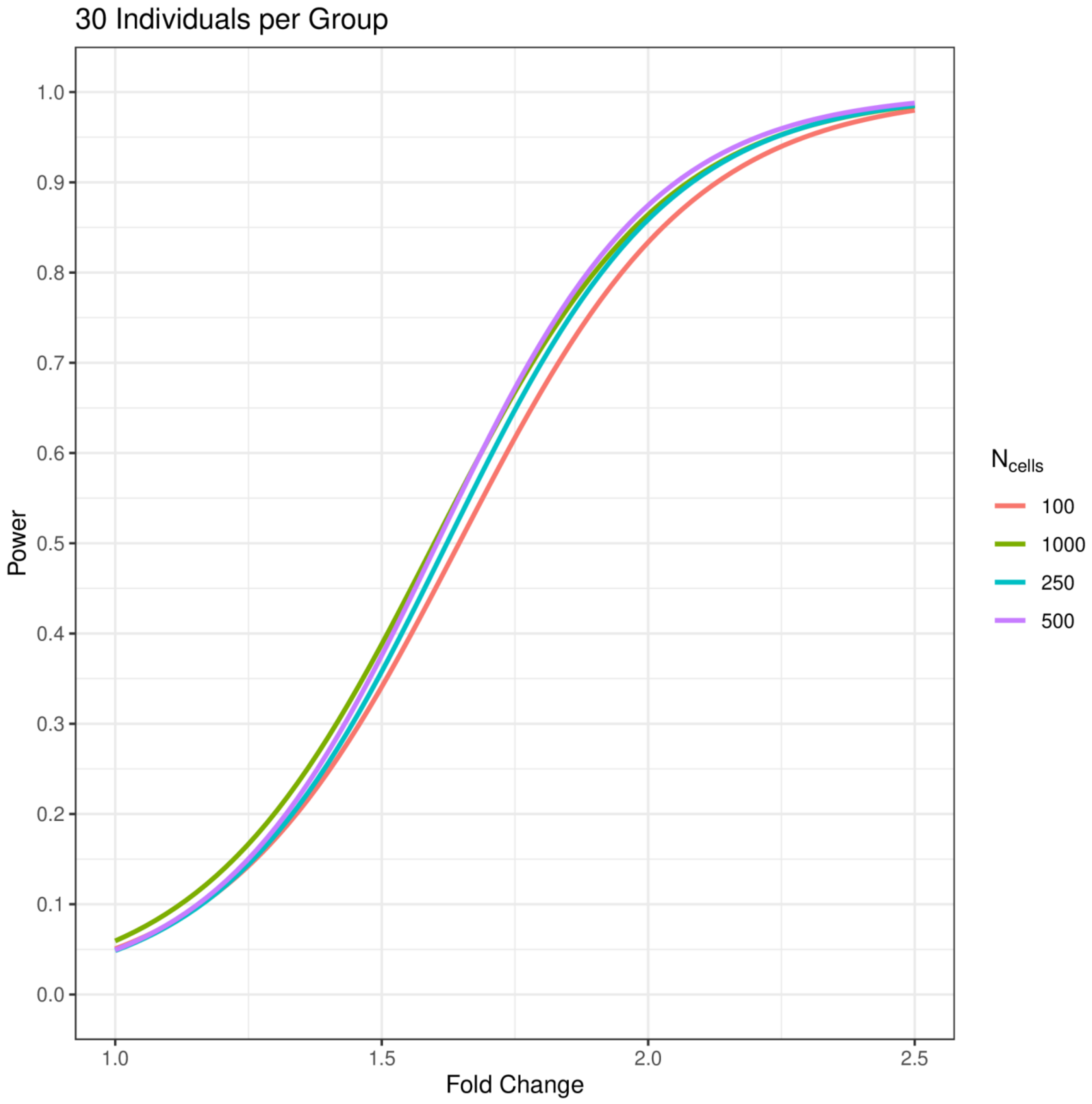
30 Individuals per Group
Power
1.0
0.9
0.8
0.7
0.6
0.5
0.4
0.3
0.2
0.1
0.0
1.0
1.5
2.0
2.5
Fold Change
N cells
100
1000
250
500

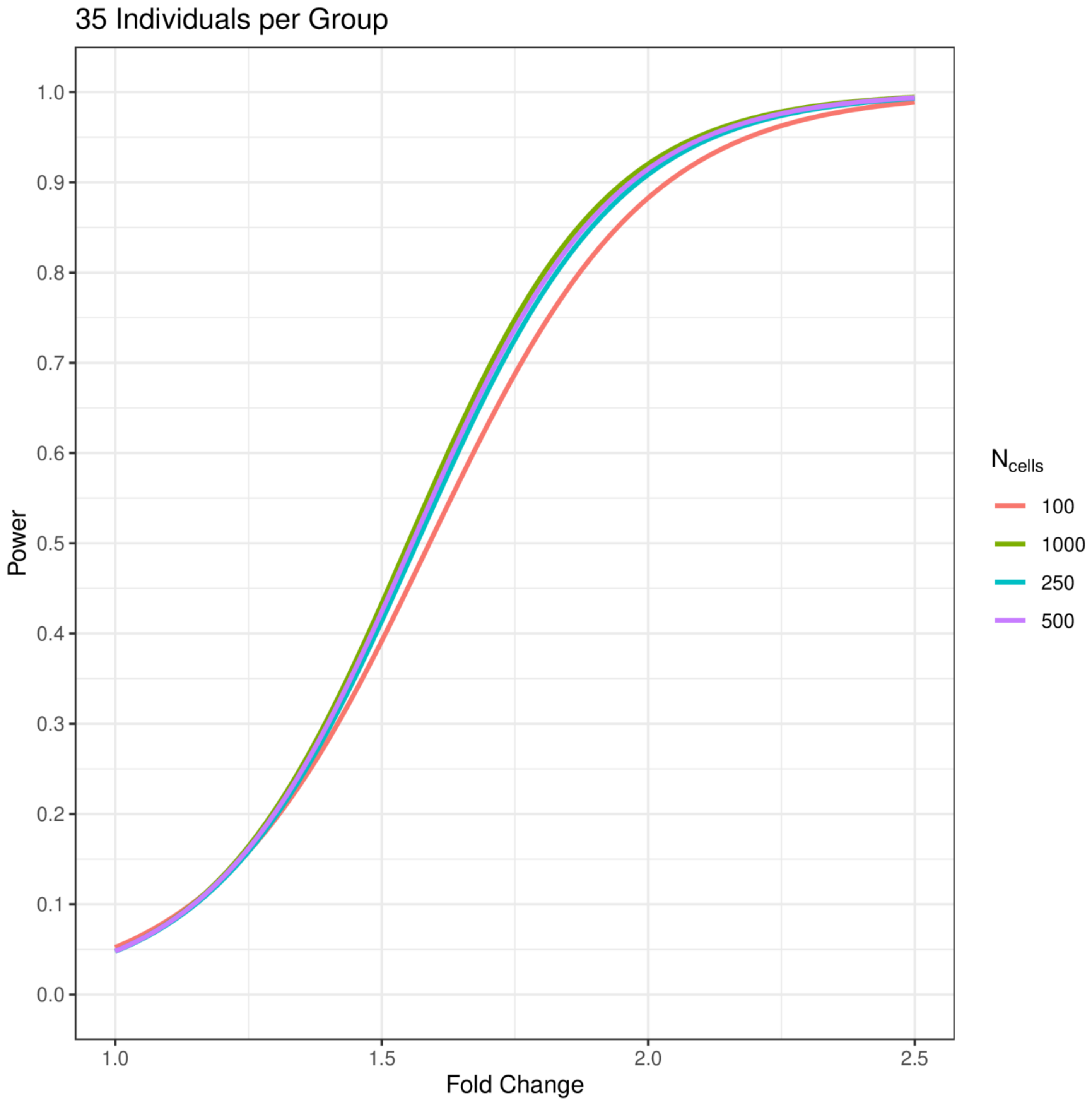

35 Individuals per Group
Power
1.0
0.9
0.8
0.7
0.6
0.5
0.4
0.3
0.2
0.1
0.0
1.0
1.5
2.0
2.5
Fold Change
N_{cells}
100
1000
250
500

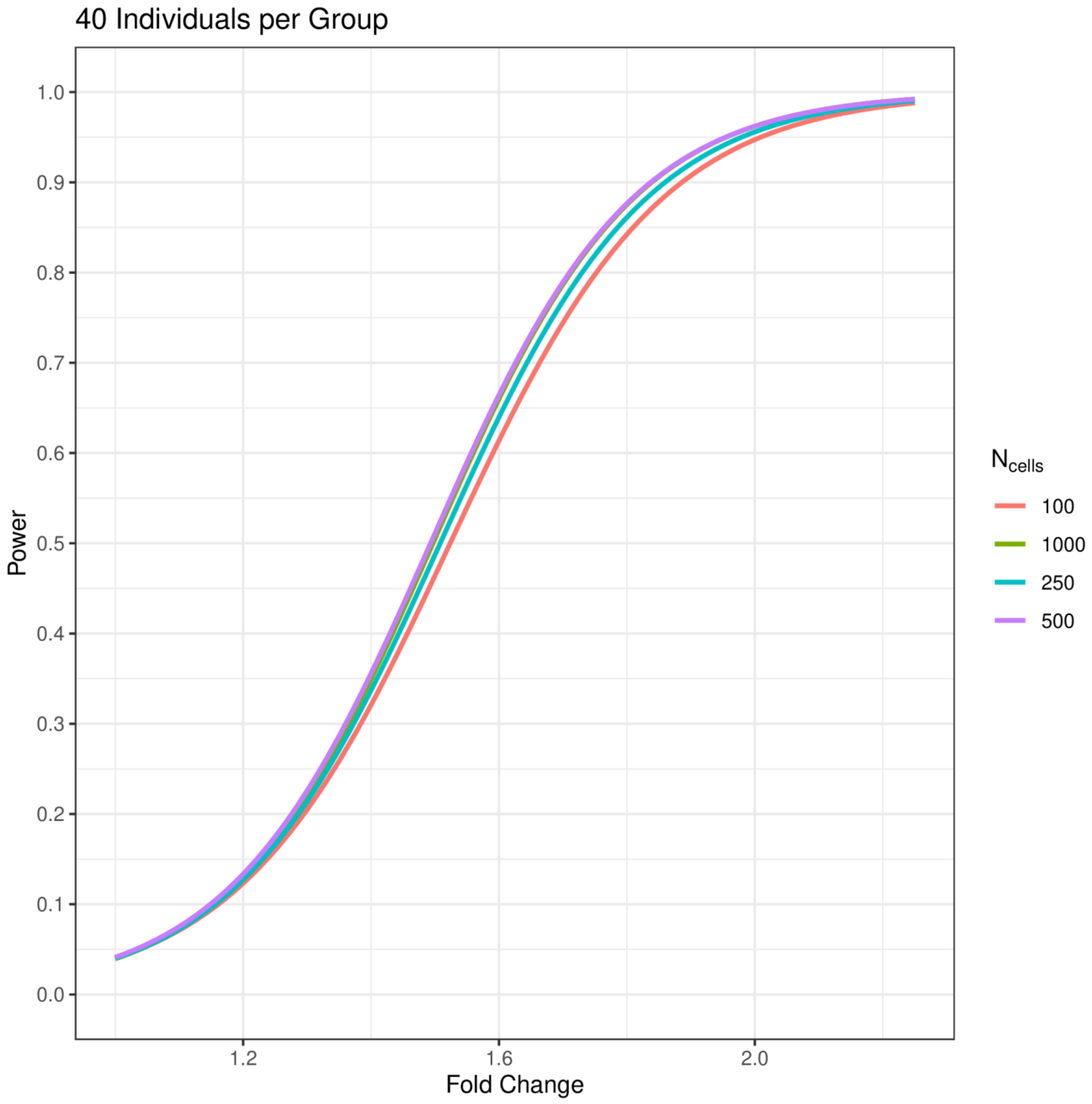
40 Individuals per Group
Power
1.0
0.9
0.8
0.7
0.6
0.5
0.4
0.3
0.2
0.1
0.0
1.2
1.6
2.0
Fold Change
N_{cells}
100
1000
250
500

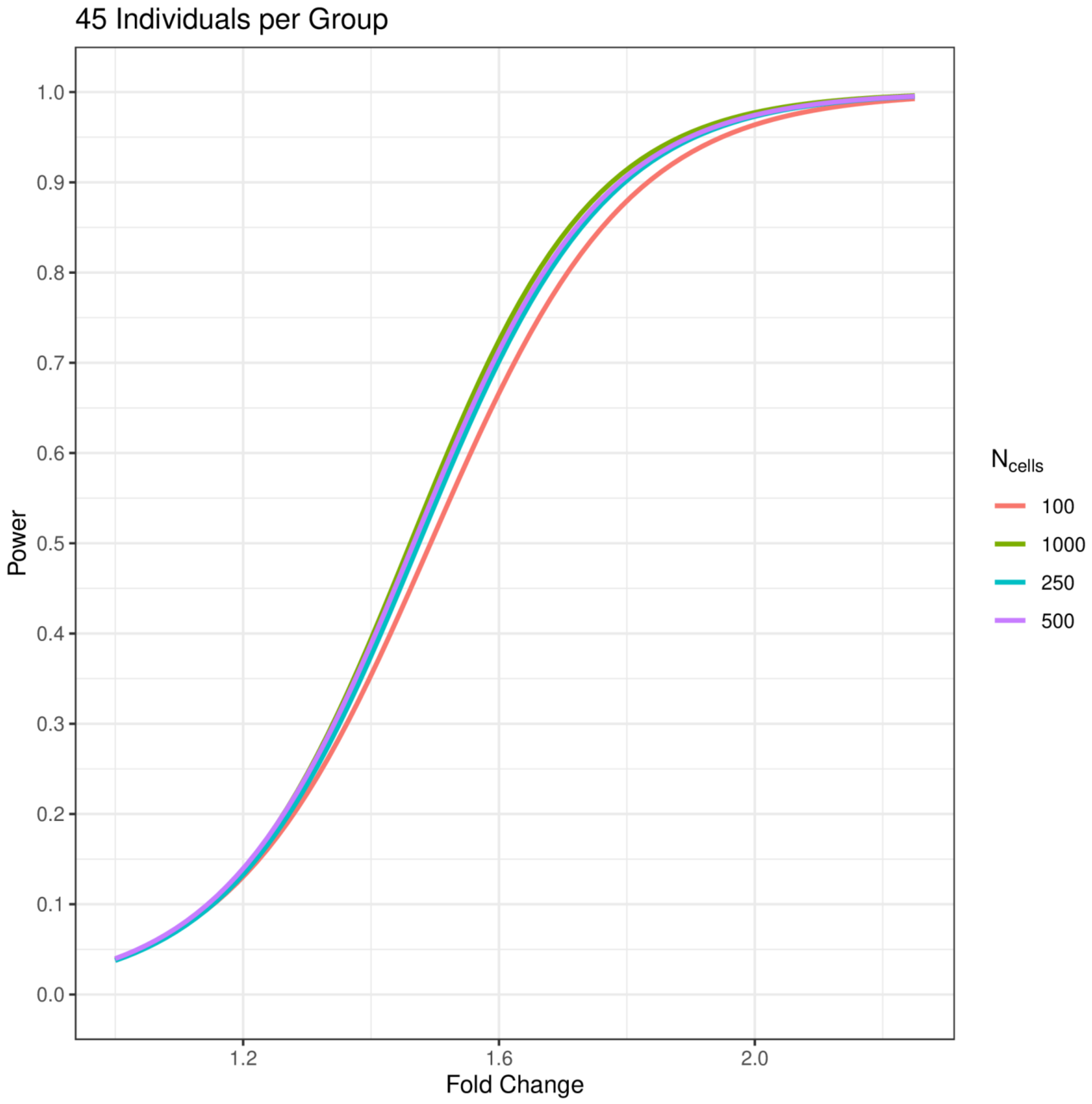
45 Individuals per Group
Power
1.0
0.9
0.8
0.7
0.6
0.5
0.4
0.3
0.2
0.1
0.0
1.2
1.6
2.0
Fold Change
N_{cells}
100
1000
250
500

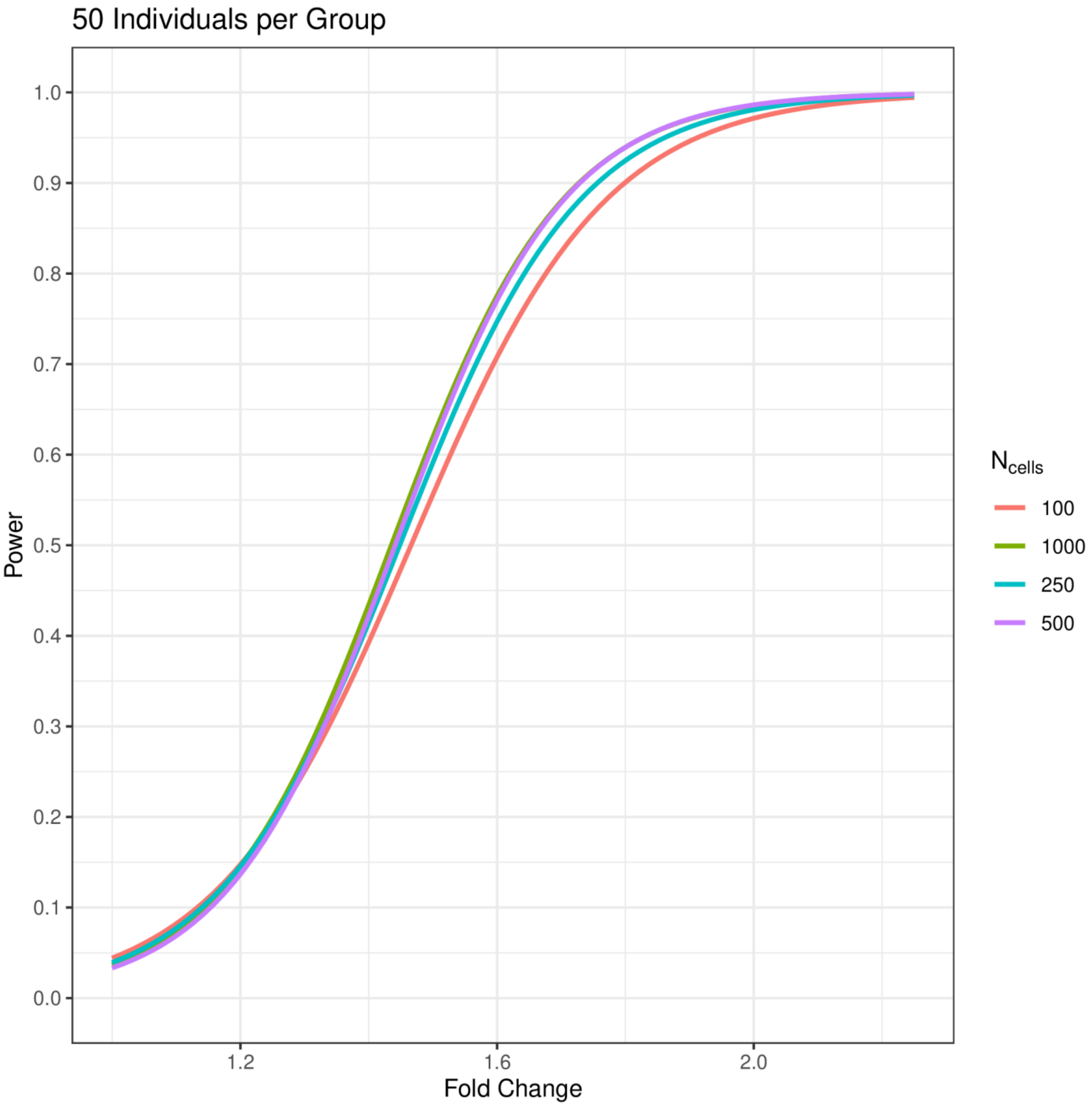
50 Individuals per Group
Power
1.0
0.9
0.8
0.7
0.6
0.5
0.4
0.3
0.2
0.1
0.0
1.2
1.6
2.0
Fold Change
N cells
100
1000
250
500

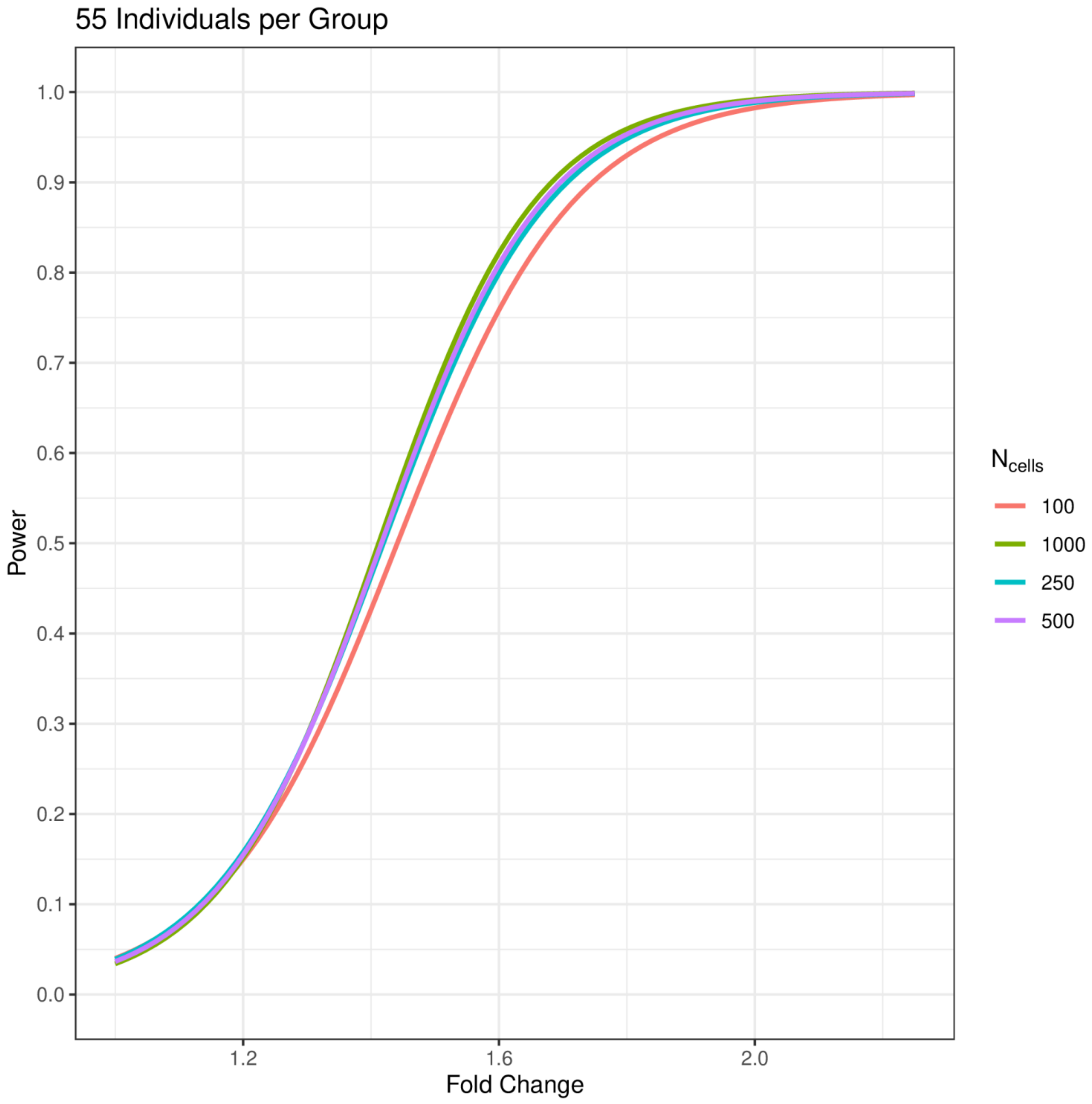
55 Individuals per Group
1.0
0.9
0.8
0.7
0.6
0.5
0.4
0.3
0.2
0.1
0.0
Power
1.2
1.6
2.0
Fold Change
N cells
100
1000
250
500

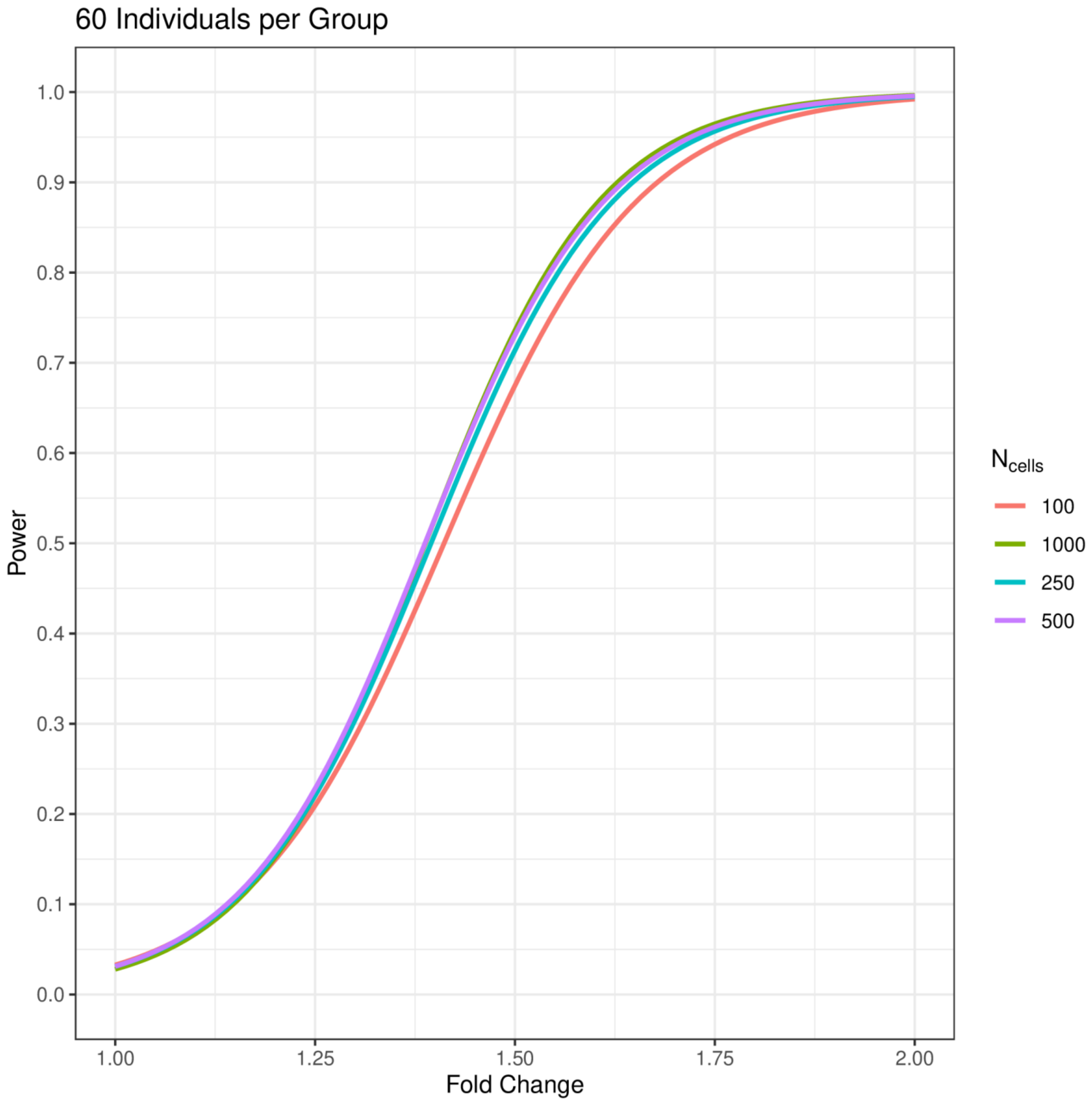
60 Individuals per Group
Power
1.0
0.9
0.8
0.7
0.6
0.5
0.4
0.3
0.2
0.1
0.0
1.00
1.25
1.50
1.75
2.00
Fold Change
N_{cells}
100
1000
250
500

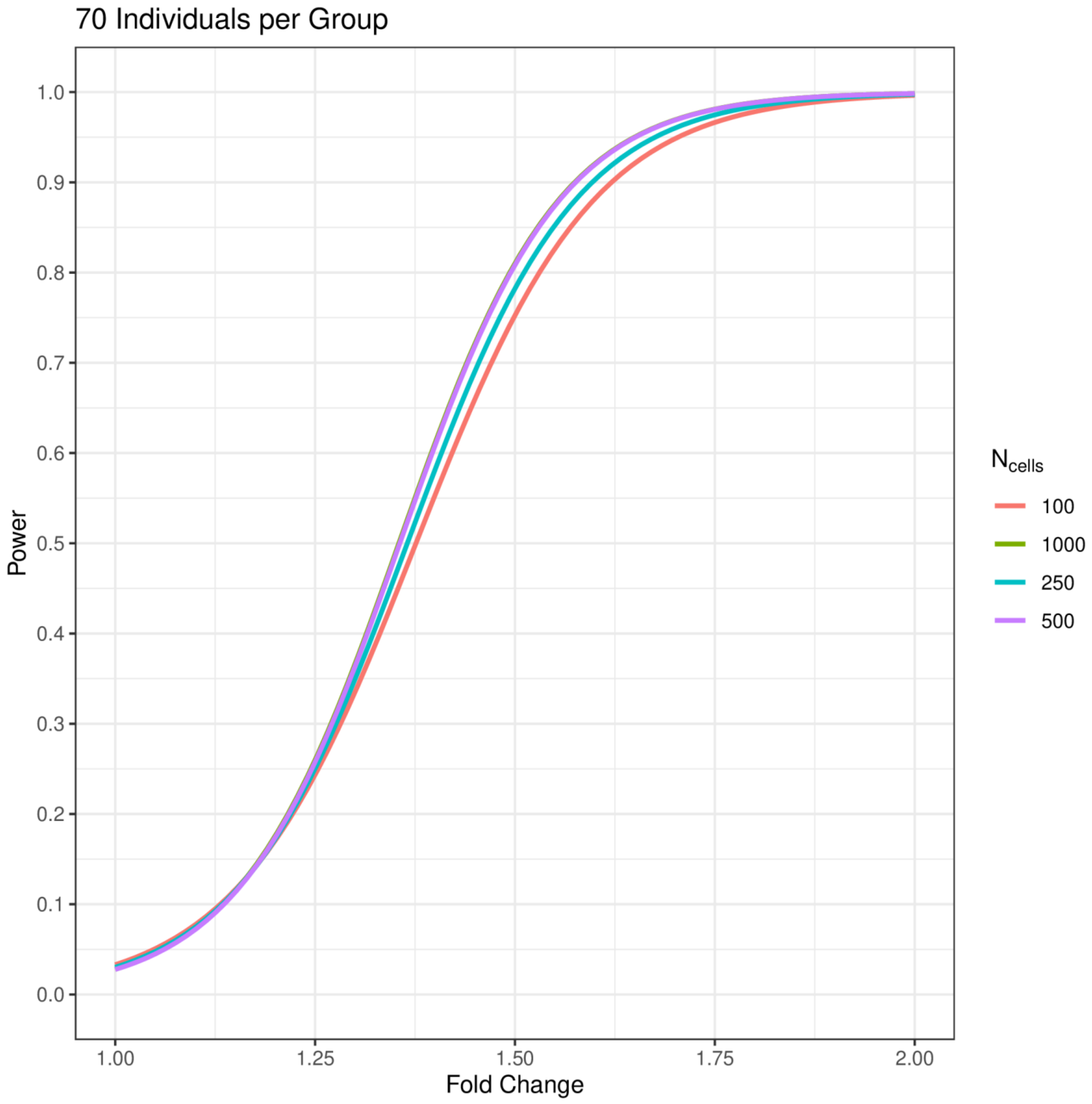
70 Individuals per Group
Power
1.0
0.9
0.8
0.7
0.6
0.5
0.4
0.3
0.2
0.1
0.0
1.00
1.25
1.50
1.75
2.00
Fold Change
N_{cells}
100
1000
250
500

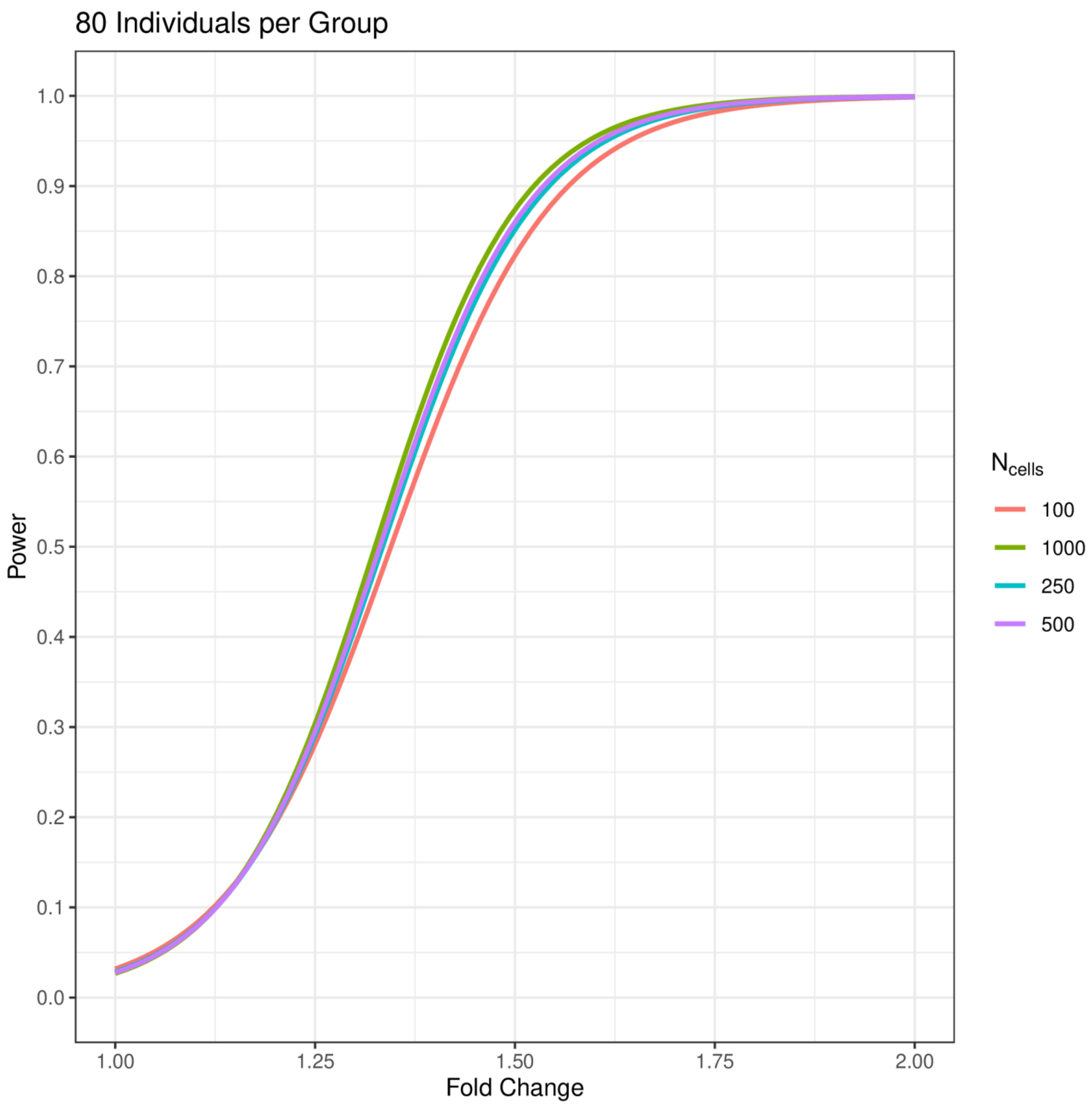
80 Individuals per Group
Power
1.0
0.9
0.8
0.7
0.6
0.5
0.4
0.3
0.2
0.1
0.0
1.00
1.25
1.50
1.75
2.00
Fold Change
N_{cells}
100
1000
250
500

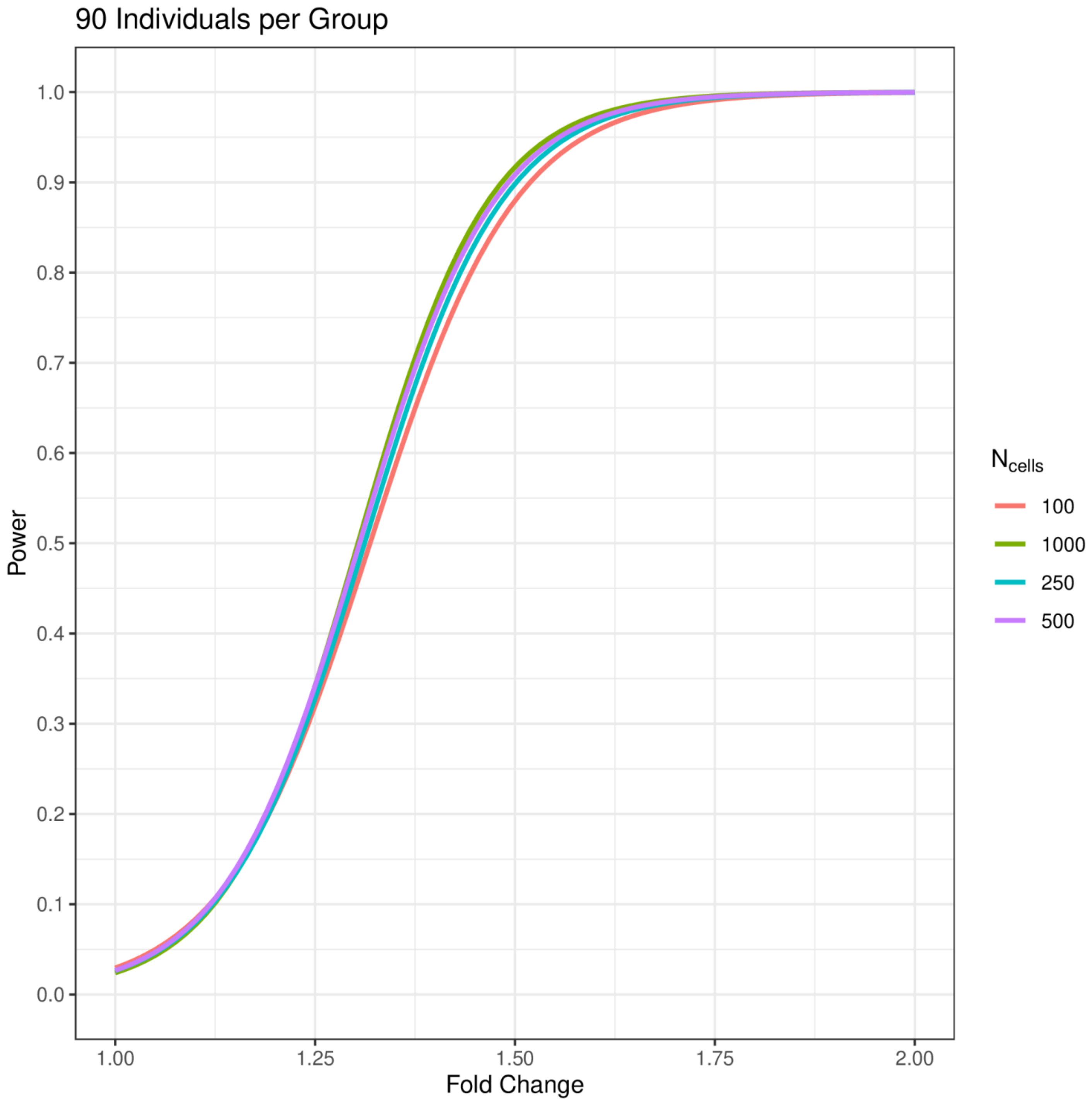
90 Individuals per Group
Power
1.0
0.9
0.8
0.7
0.6
0.5
0.4
0.3
0.2
0.1
0.0
1.00
1.25
1.50
1.75
2.00
Fold Change
N_{cells}
100
1000
250
500

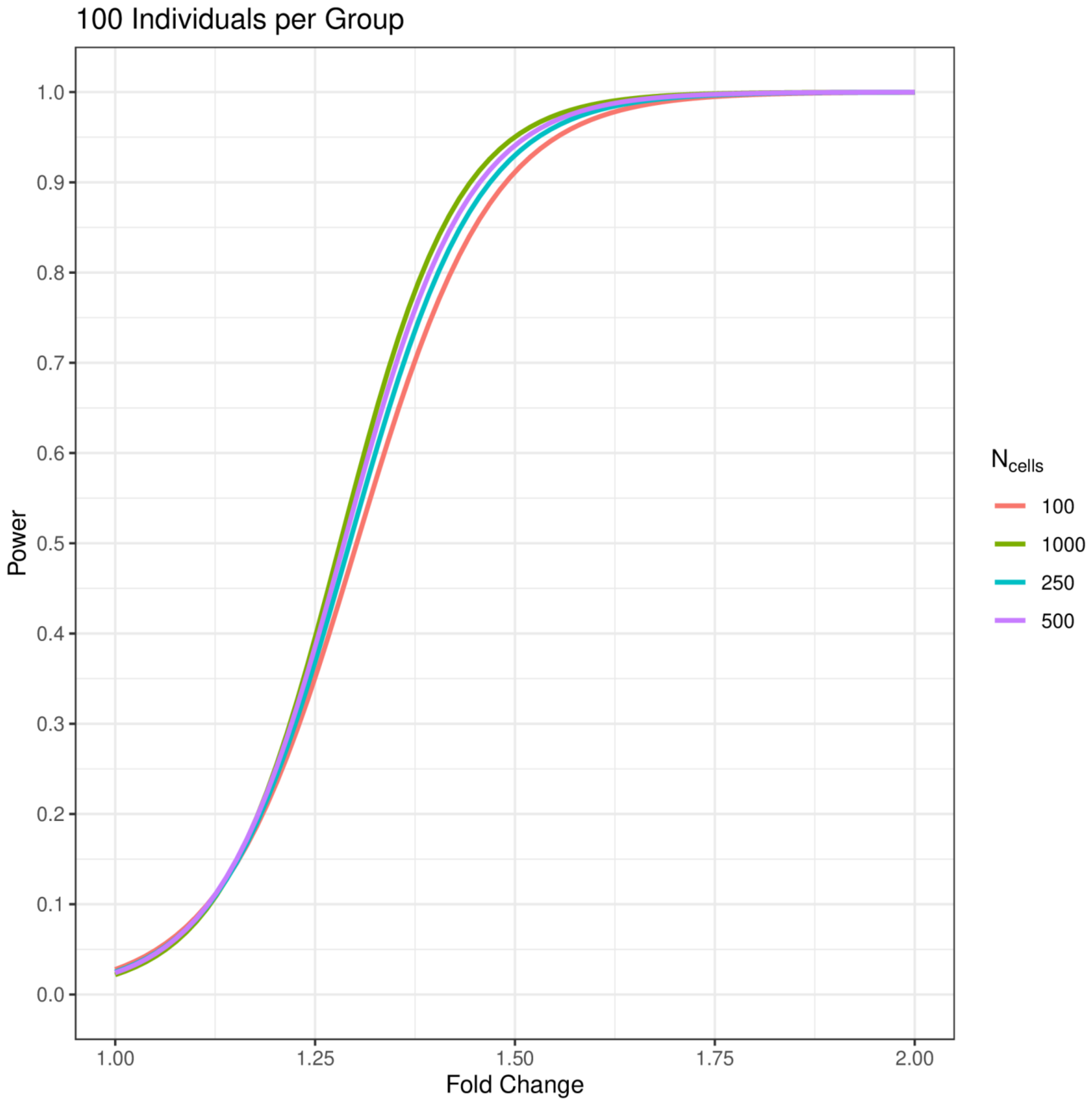
100 Individuals per Group
Power
Fold Change
1.0
0.9
0.8
0.7
0.6
0.5
0.4
0.3
0.2
0.1
0.0
1.00
1.25
1.50
1.75
2.00
N_{cells}
100
1000
250
500

Supplementary Fig. 11: Power calculations using MAST with a random effect for the individual. Power curves for MAST using a random effect to account for intra-individual correlation. Curves are computed for 100 (red line), 250, Blue line) 500 (purple line), and 1,000 (red line) cells per individual with $\alpha = 0.001$. The numbers of individuals per group range from 5 to 100 and are listed above each plot.

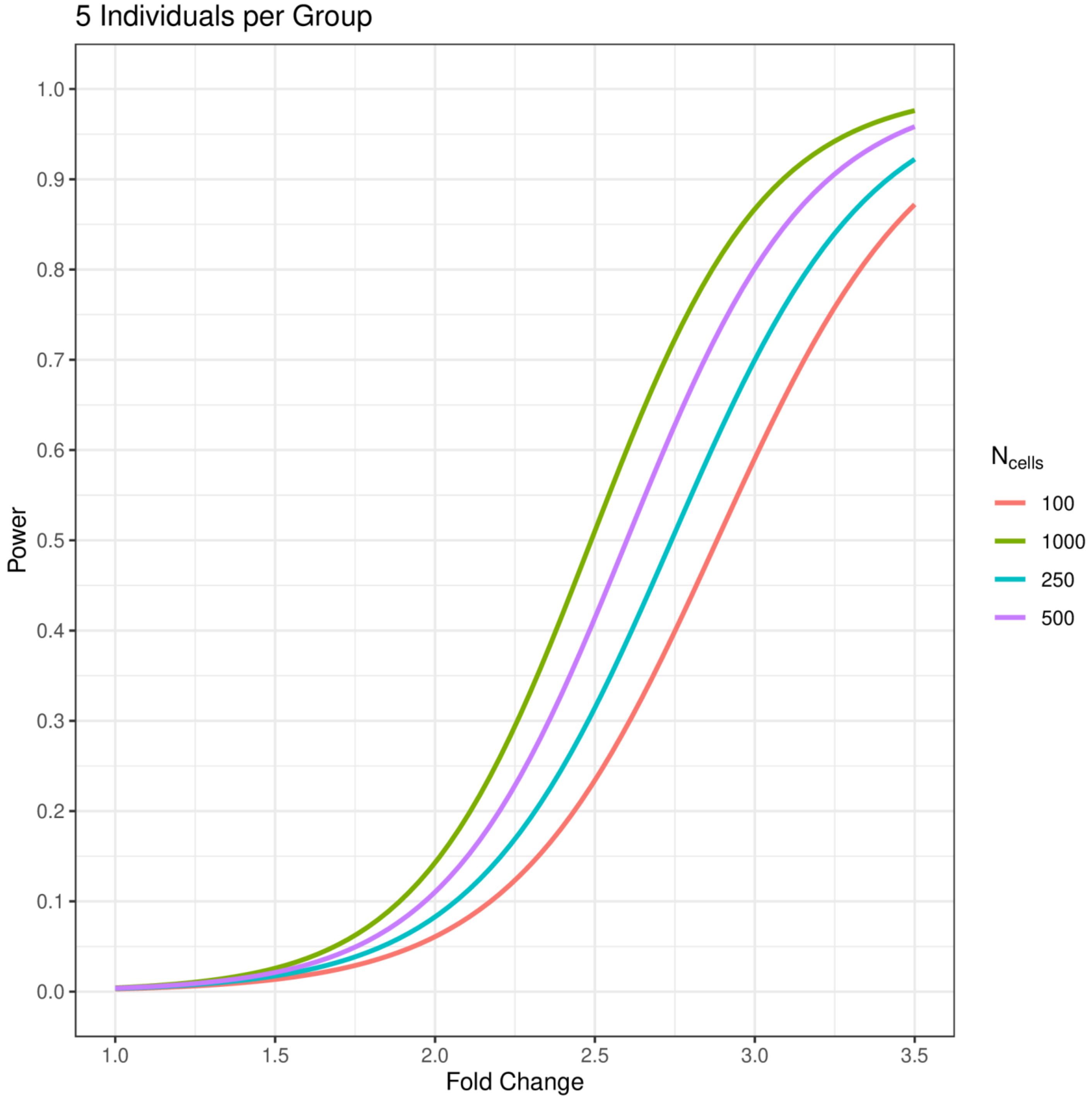

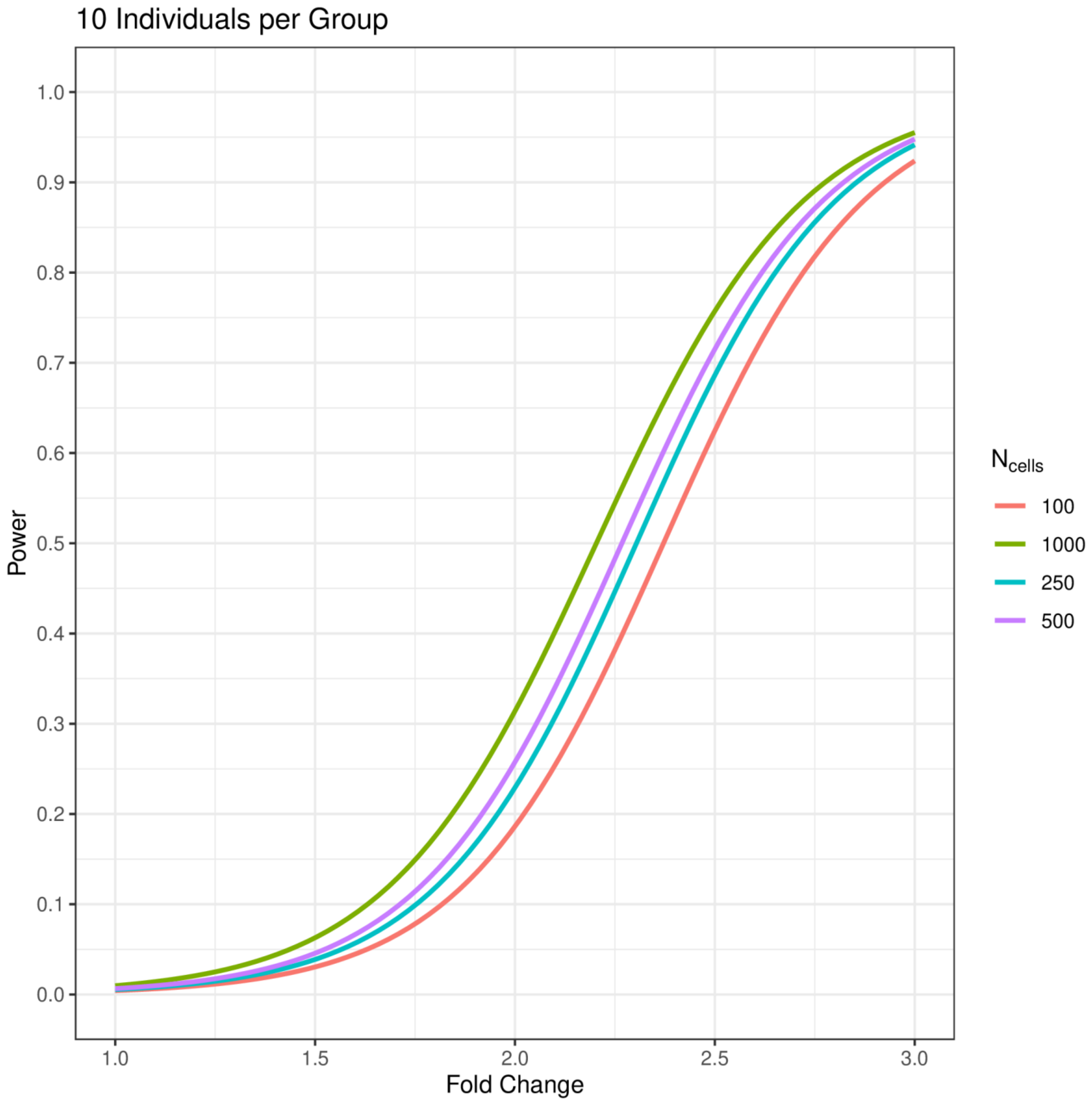
10 Individuals per Group
1.0
0.9
0.8
0.7
0.6
0.5
0.4
0.3
0.2
0.1
0.0
Power
1.0
1.5
2.0
2.5
3.0
Fold Change
N cells
100
1000
250
500

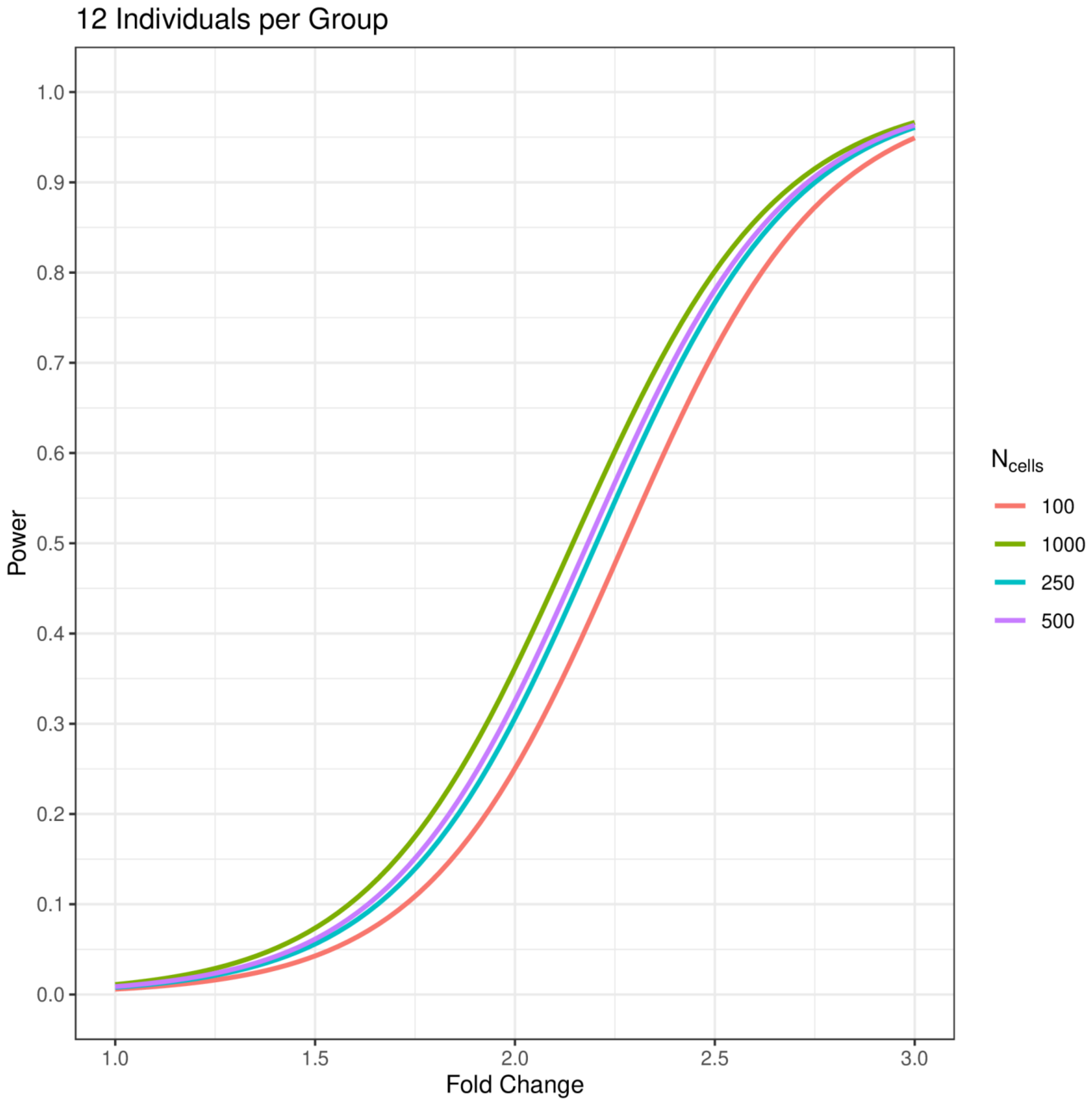
12 Individuals per Group
Power
Fold Change
1.0
0.9
0.8
0.7
0.6
0.5
0.4
0.3
0.2
0.1
0.0
1.0
1.5
2.0
2.5
3.0
N_{cells}
100
1000
250
500

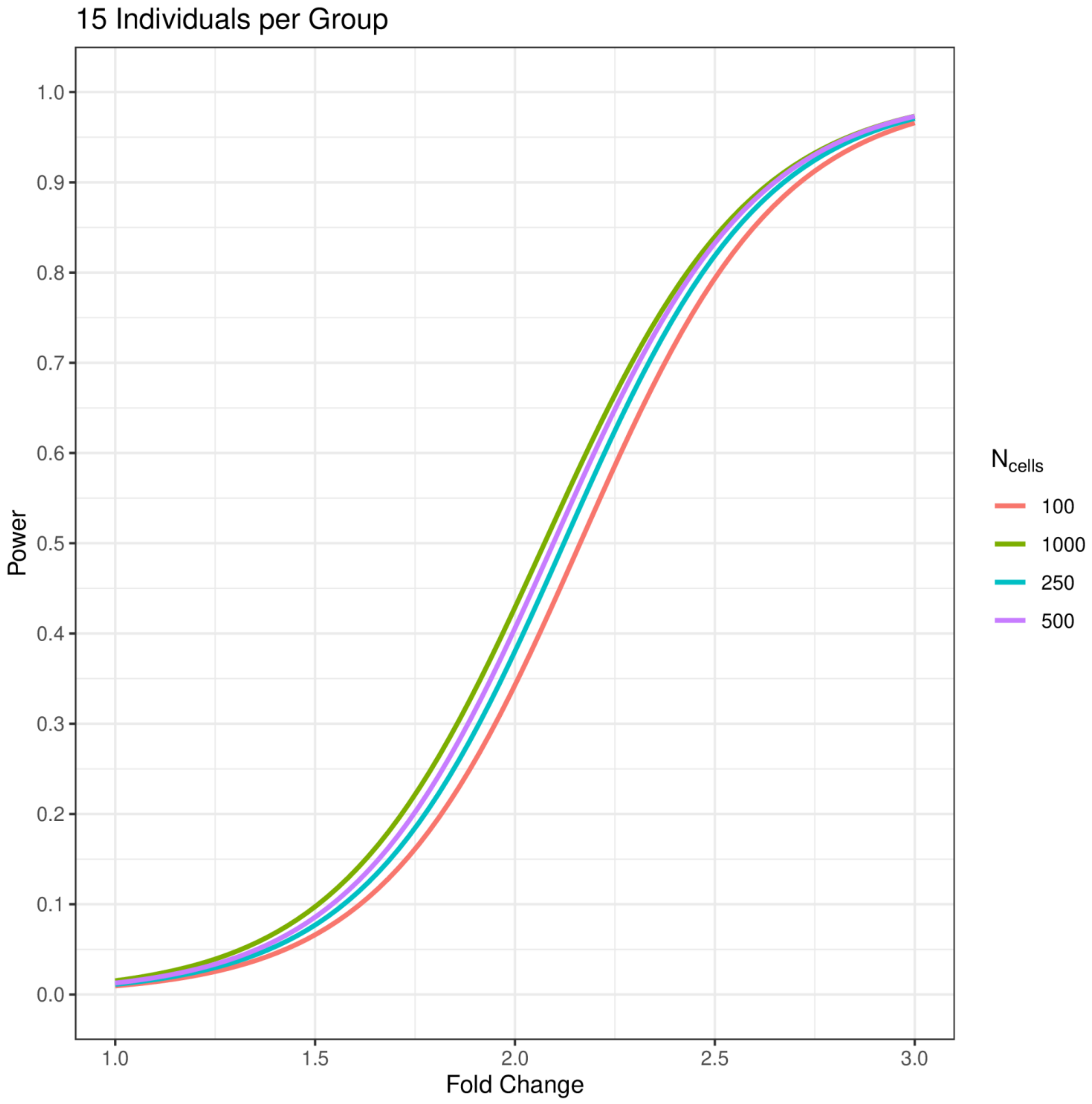
15 Individuals per Group
Power
1.0
0.9
0.8
0.7
0.6
0.5
0.4
0.3
0.2
0.1
0.0
1.0
1.5
2.0
2.5
3.0
Fold Change
N_{cells}
100
1000
250
500

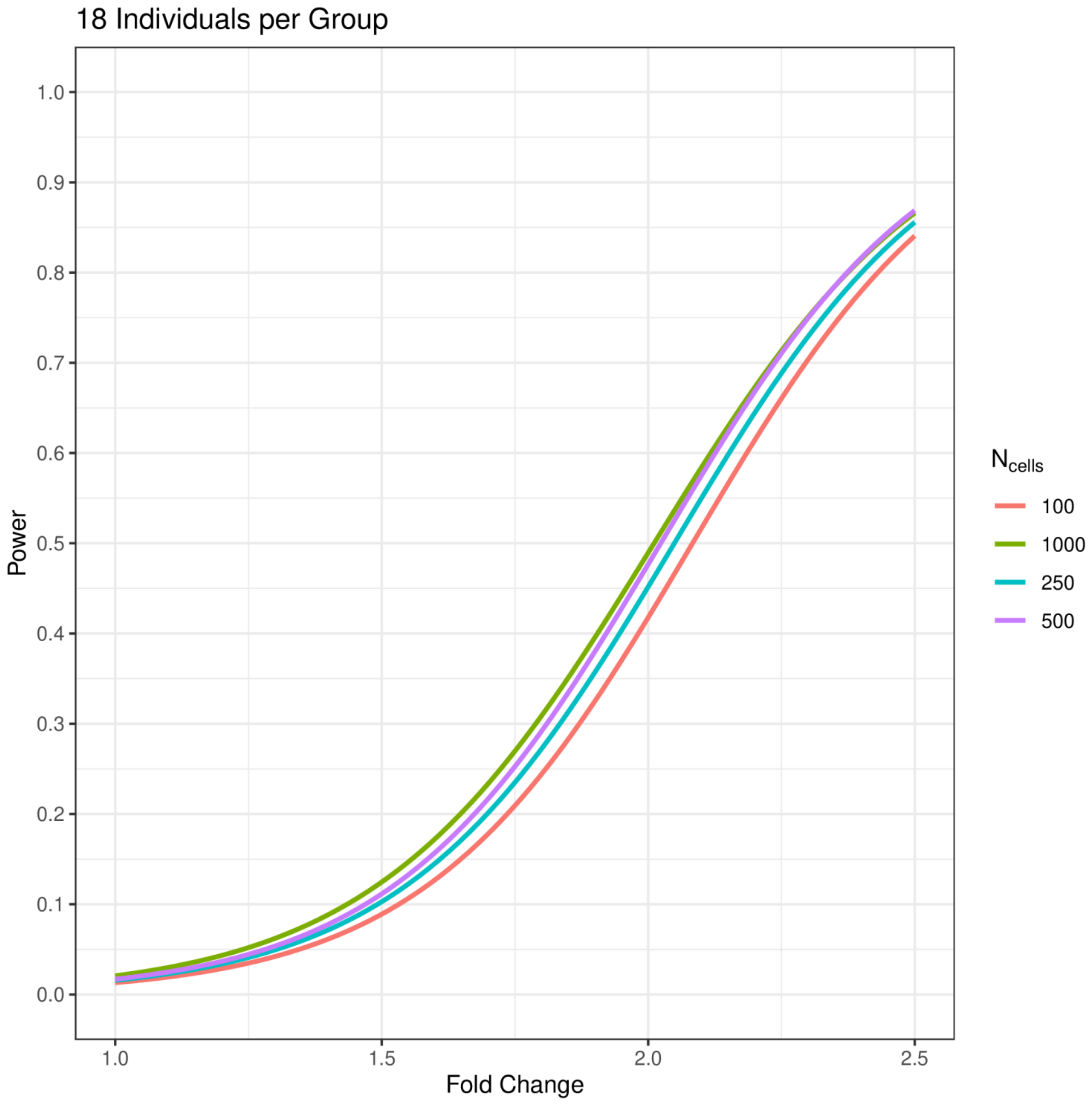
18 Individuals per Group
Power
1.0
0.9
0.8
0.7
0.6
0.5
0.4
0.3
0.2
0.1
0.0
1.0
1.5
2.0
2.5
Fold Change
N_{cells}
100
1000
250
500

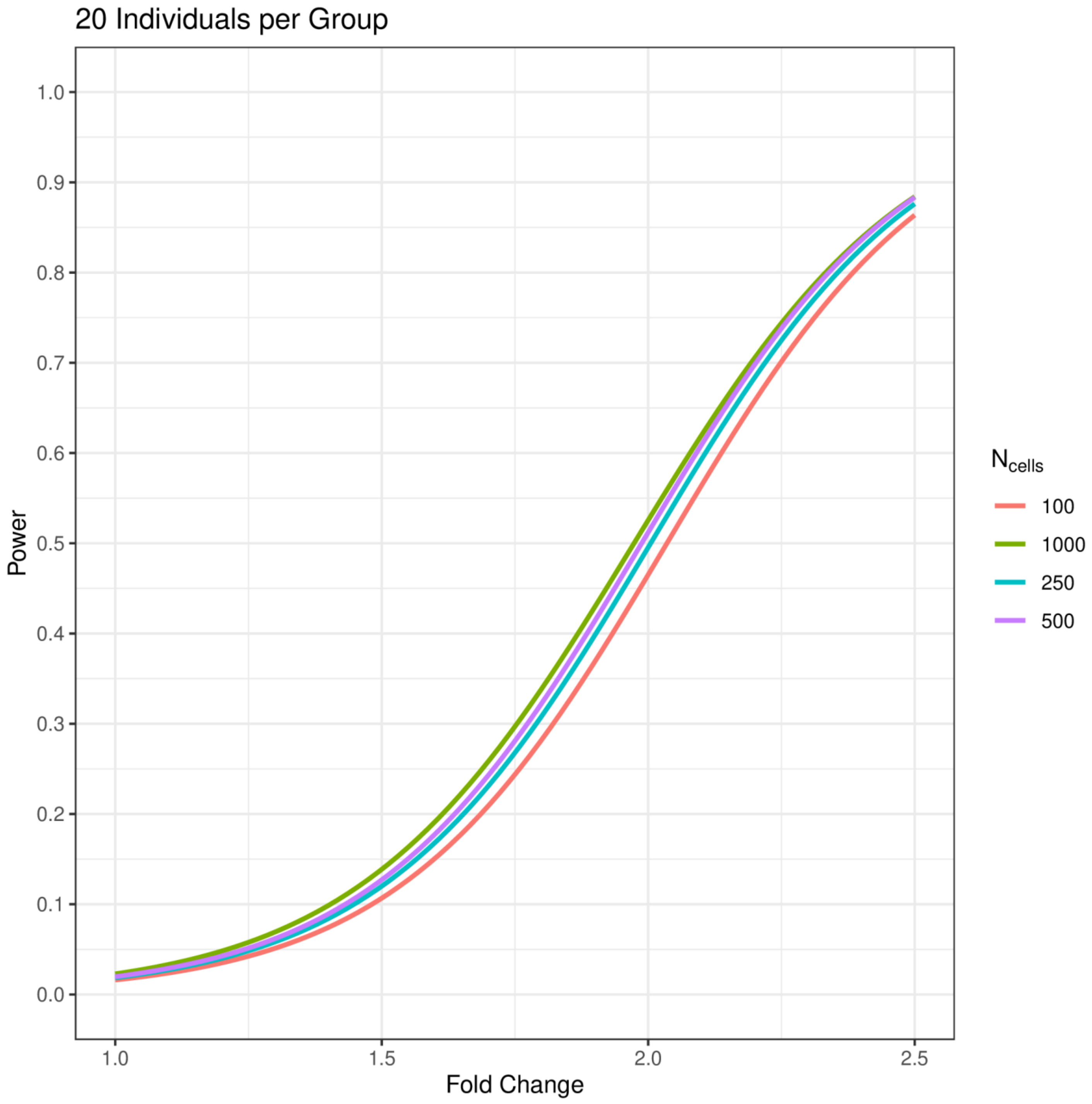
20 Individuals per Group
Power
1.0
0.9
0.8
0.7
0.6
0.5
0.4
0.3
0.2
0.1
0.0
1.0
1.5
2.0
2.5
Fold Change
N_{cells}
100
1000
250
500

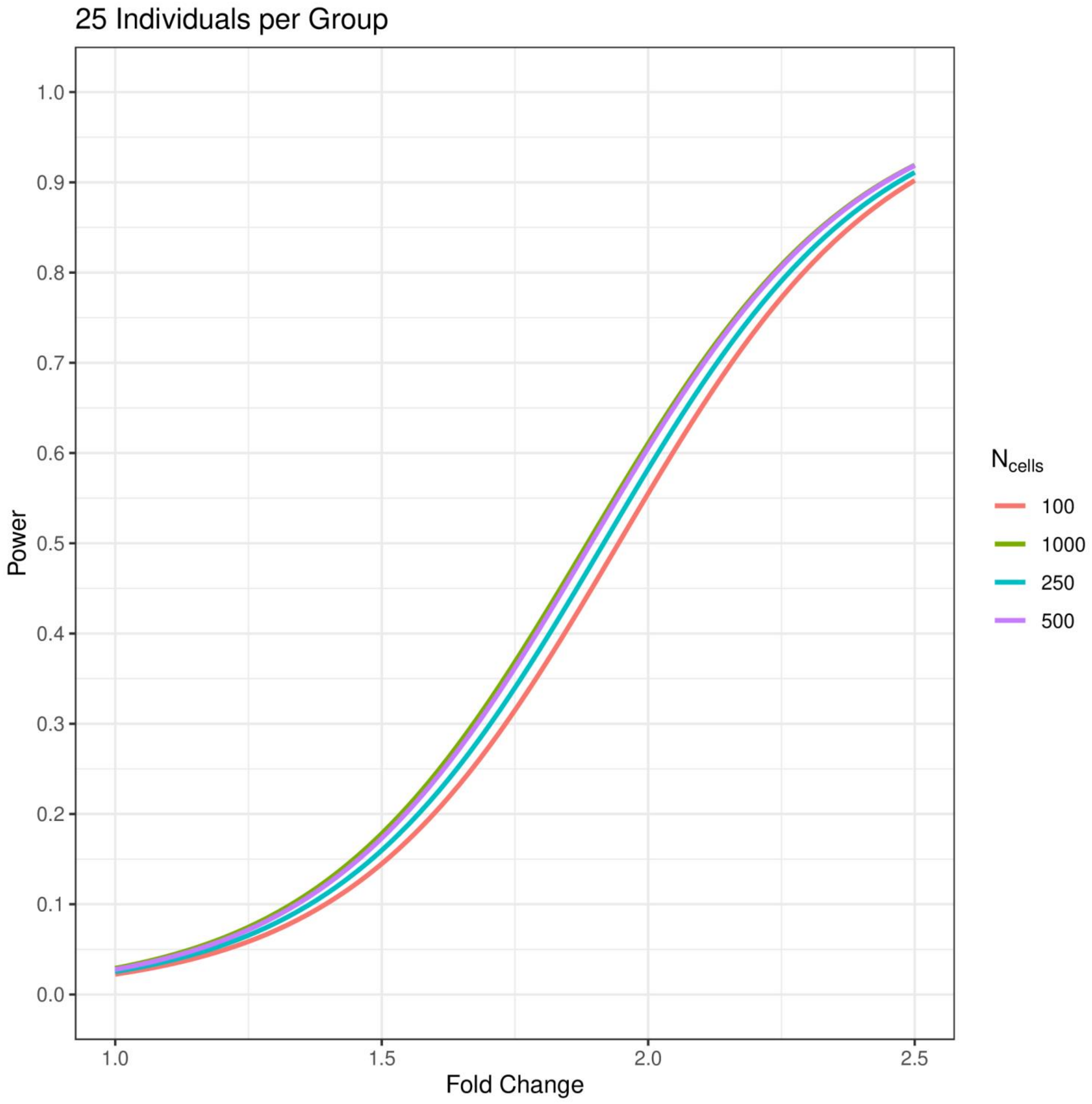
25 Individuals per Group
Power
1.0
0.9
0.8
0.7
0.6
0.5
0.4
0.3
0.2
0.1
0.0
1.0
1.5
2.0
2.5
Fold Change
N_{cells}
100
1000
250
500

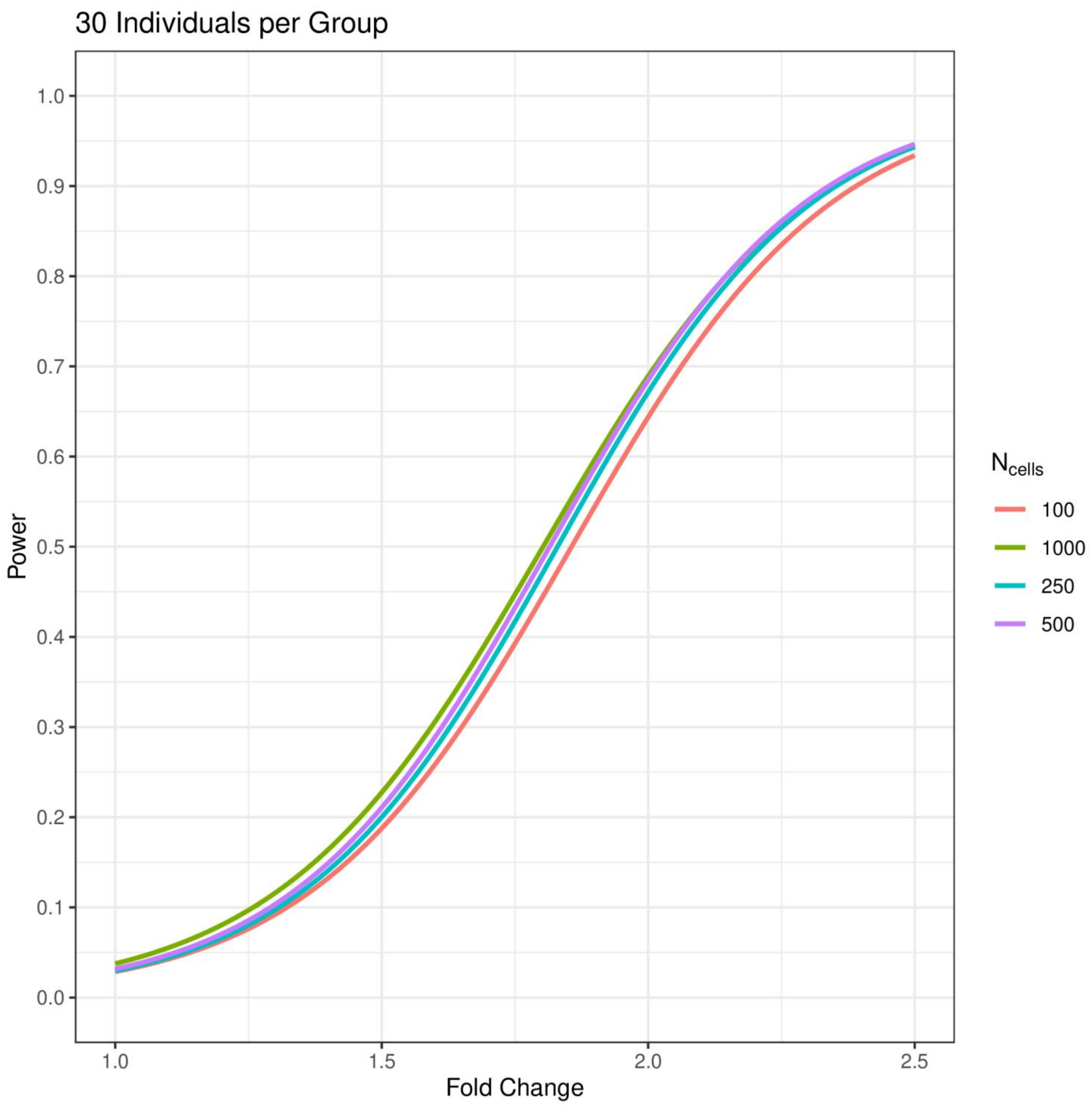
30 Individuals per Group
Power
1.0
0.9
0.8
0.7
0.6
0.5
0.4
0.3
0.2
0.1
0.0
1.0
1.5
2.0
2.5
Fold Change
N_{cells}
100
1000
250
500

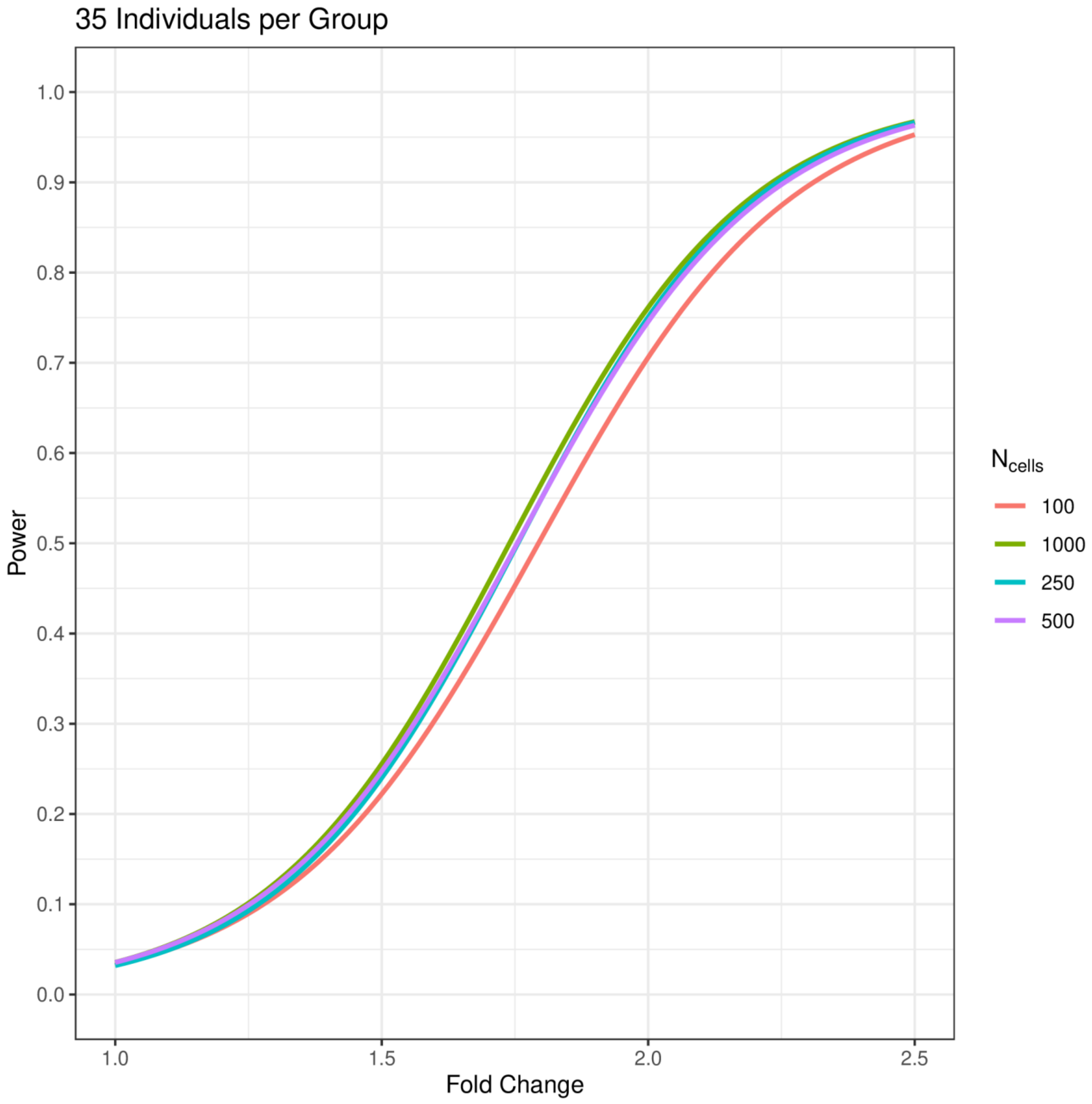

35 Individuals per Group
Power
1.0
0.9
0.8
0.7
0.6
0.5
0.4
0.3
0.2
0.1
0.0
1.0
1.5
2.0
2.5
Fold Change
N_{cells}
100
1000
250
500

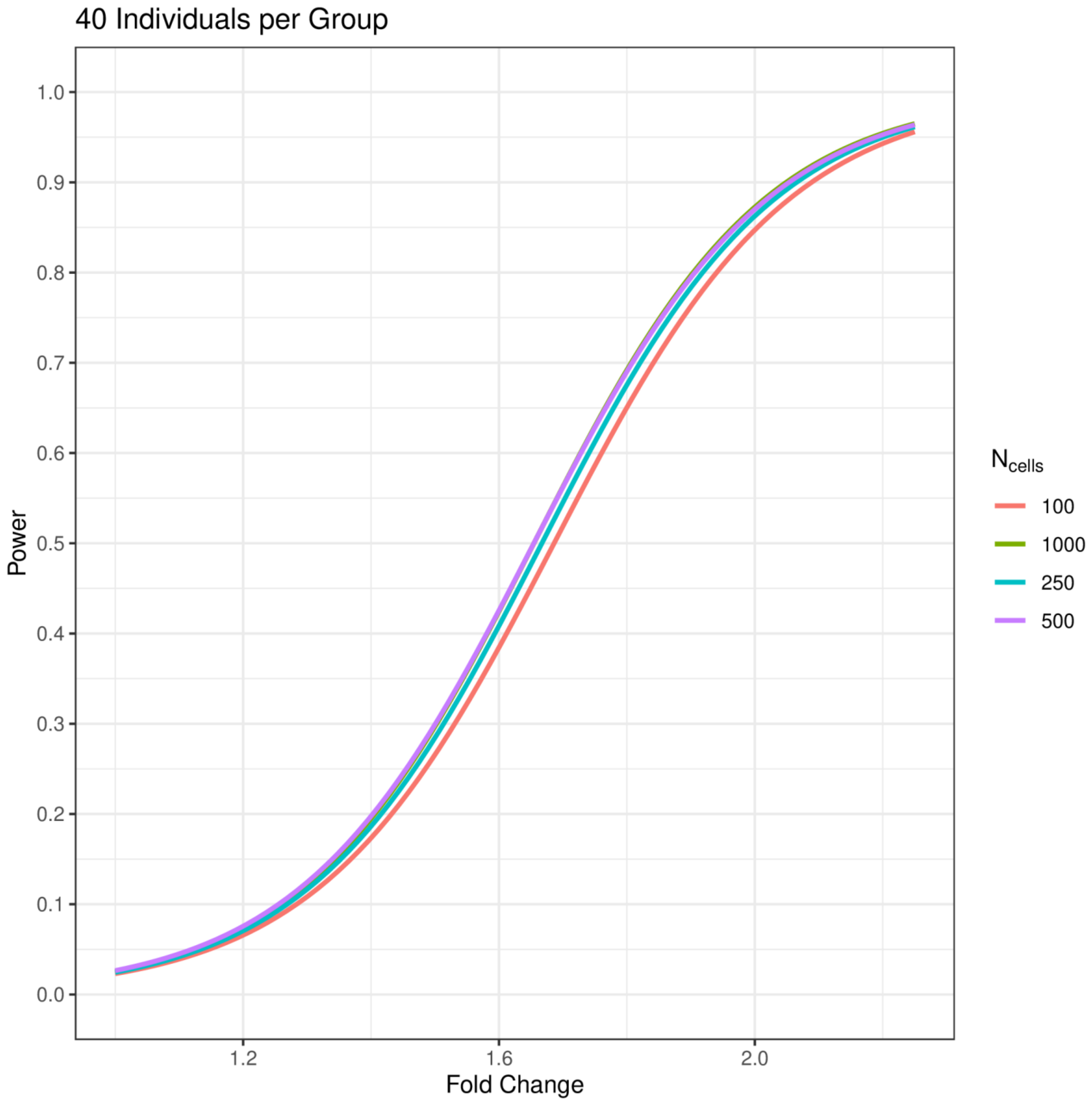

40 Individuals per Group
Power
1.0
0.9
0.8
0.7
0.6
0.5
0.4
0.3
0.2
0.1
0.0
1.2
1.6
2.0
Fold Change
N_{cells}
100
1000
250
500

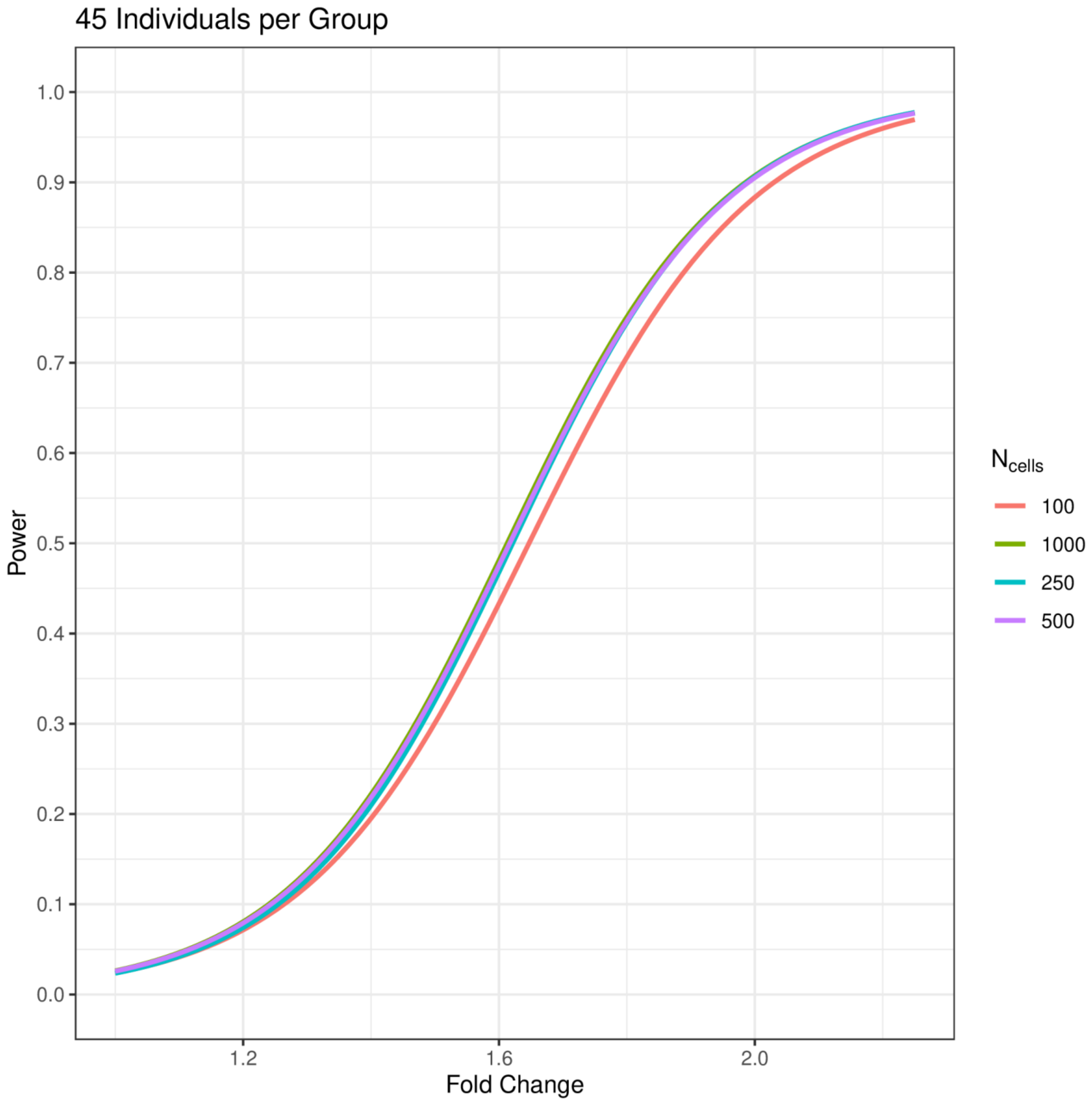
45 Individuals per Group
Power
Fold Change
1.0
0.9
0.8
0.7
0.6
0.5
0.4
0.3
0.2
0.1
0.0
1.2
1.6
2.0
Ncells
100
1000
250
500

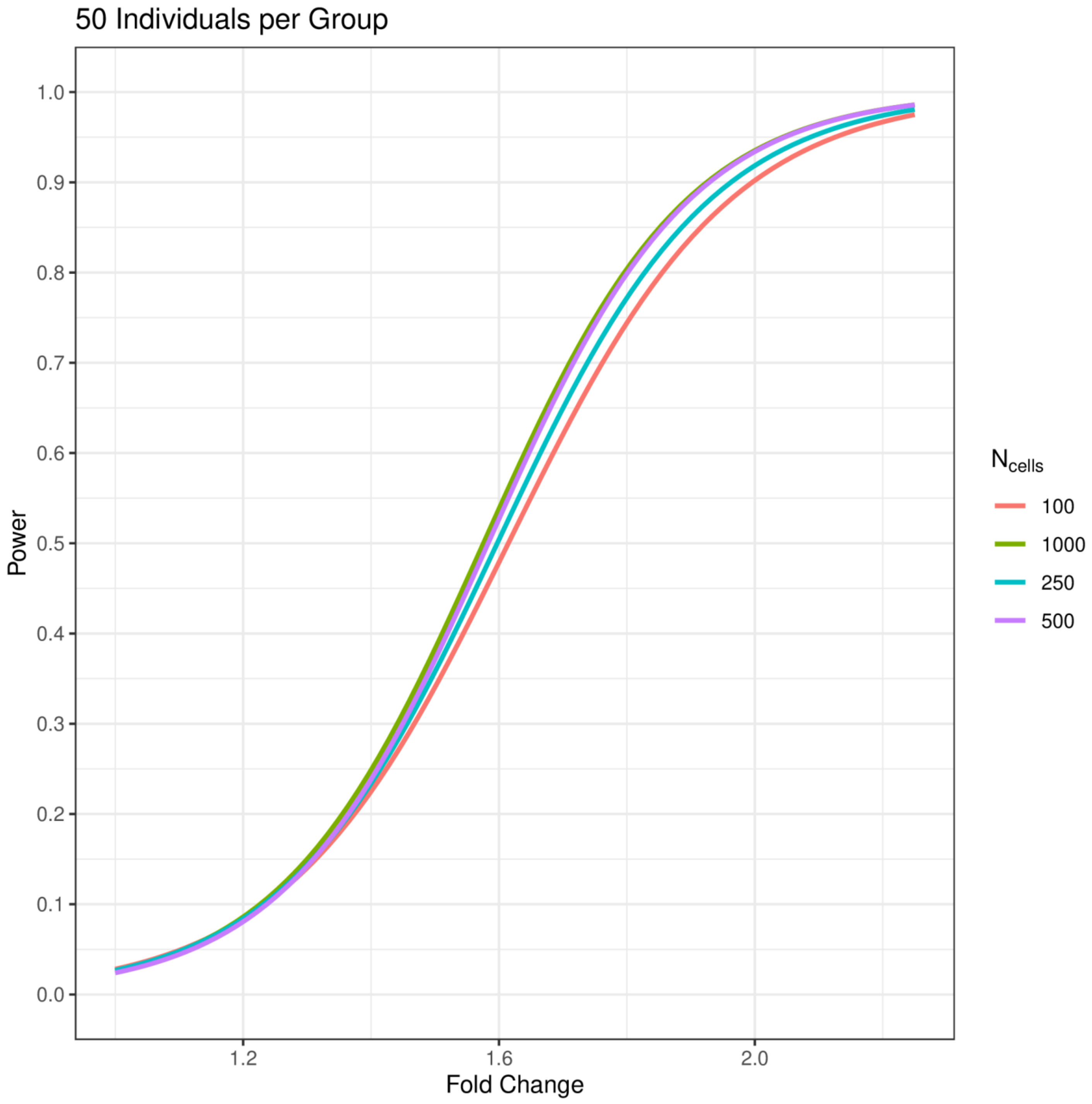
50 Individuals per Group
Power
1.0
0.9
0.8
0.7
0.6
0.5
0.4
0.3
0.2
0.1
0.0
1.2
1.6
2.0
Fold Change
N_{cells}
100
1000
250
500

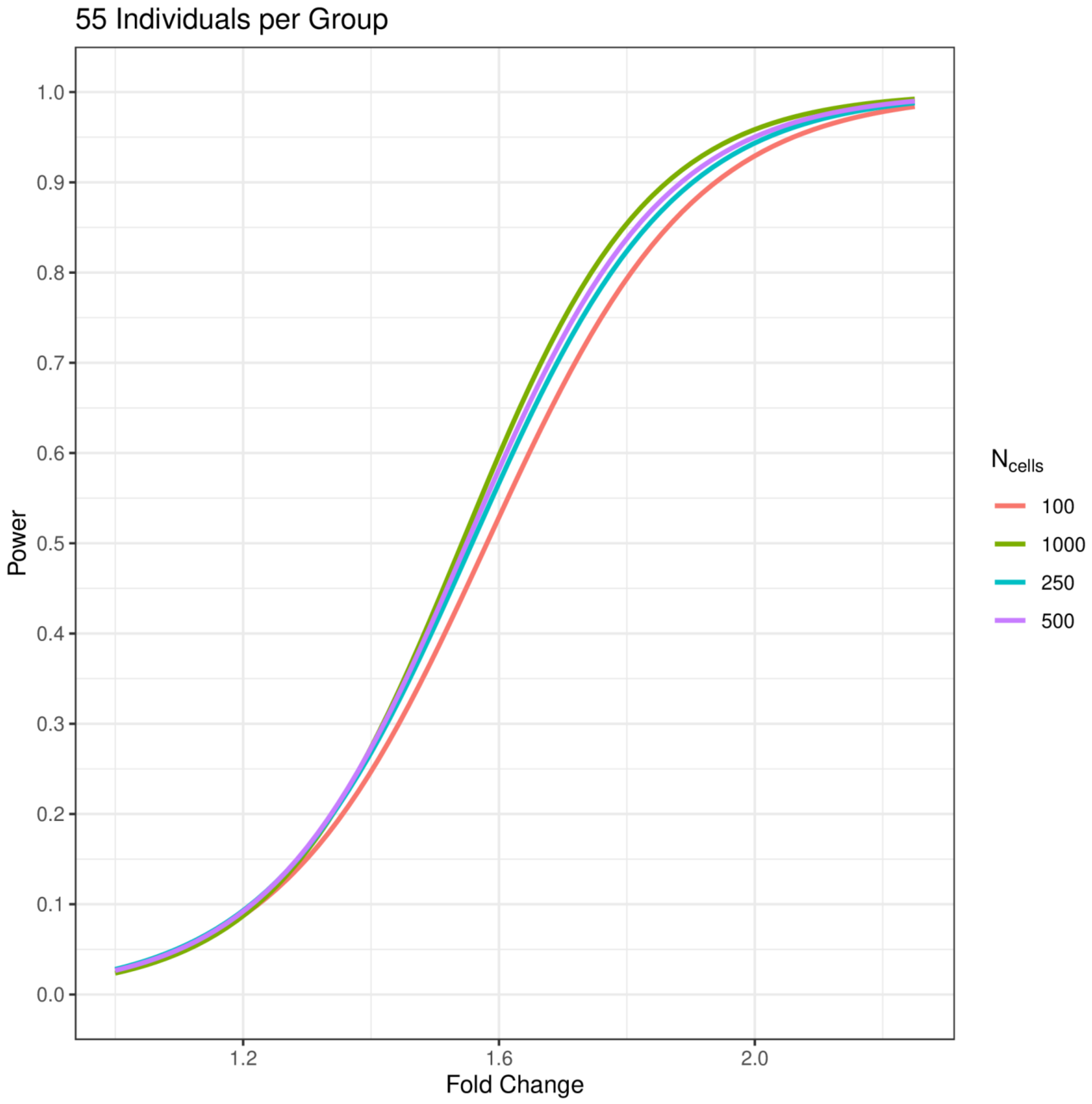
55 Individuals per Group
Power
1.0
0.9
0.8
0.7
0.6
0.5
0.4
0.3
0.2
0.1
0.0
1.2
1.6
2.0
Fold Change
N_{cells}
100
1000
250
500

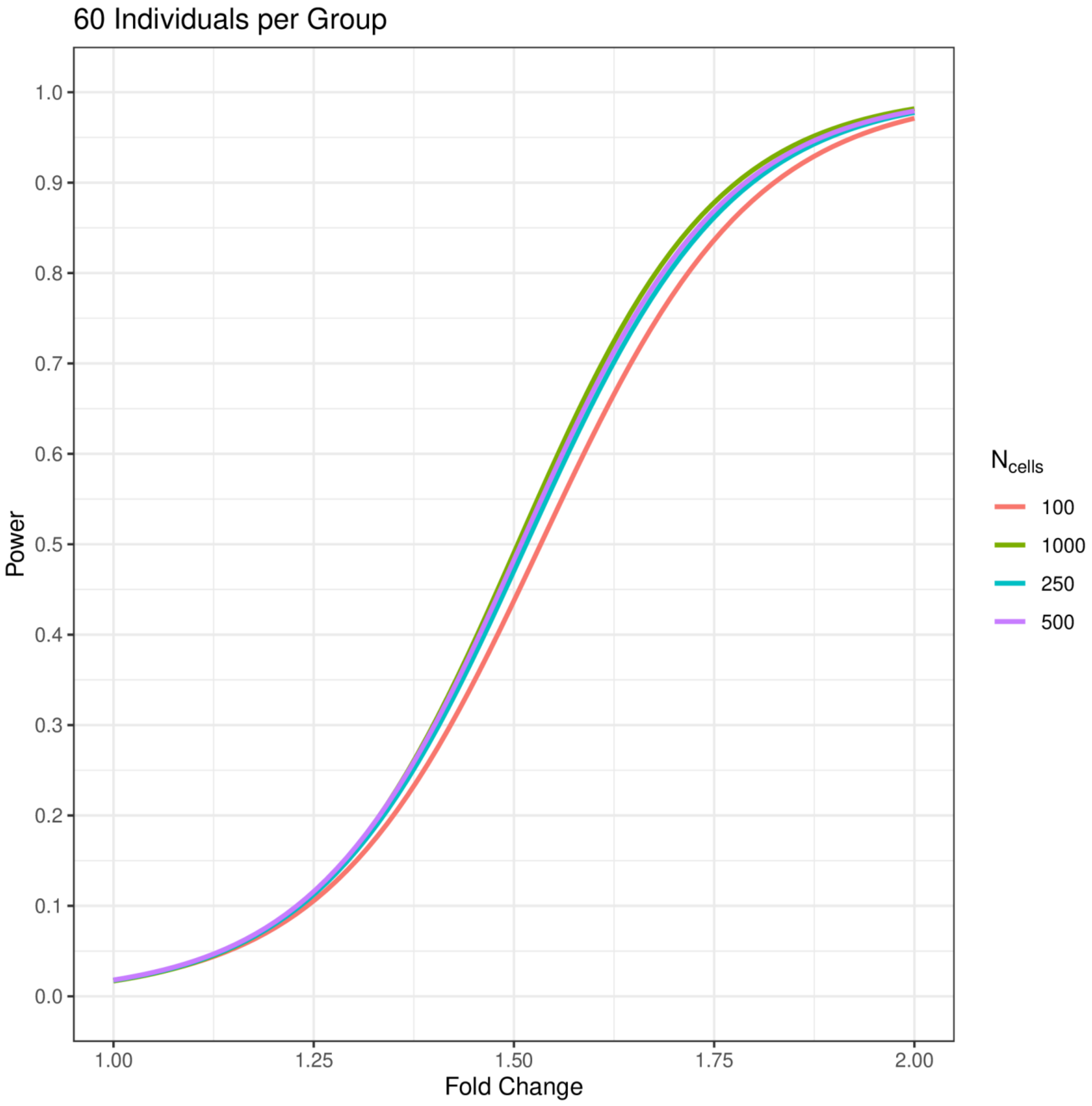
60 Individuals per Group
1.0
0.9
0.8
0.7
0.6
0.5
0.4
0.3
0.2
0.1
0.0
Power
1.00
1.25
1.50
1.75
2.00
Fold Change
N cells
100
1000
250
500

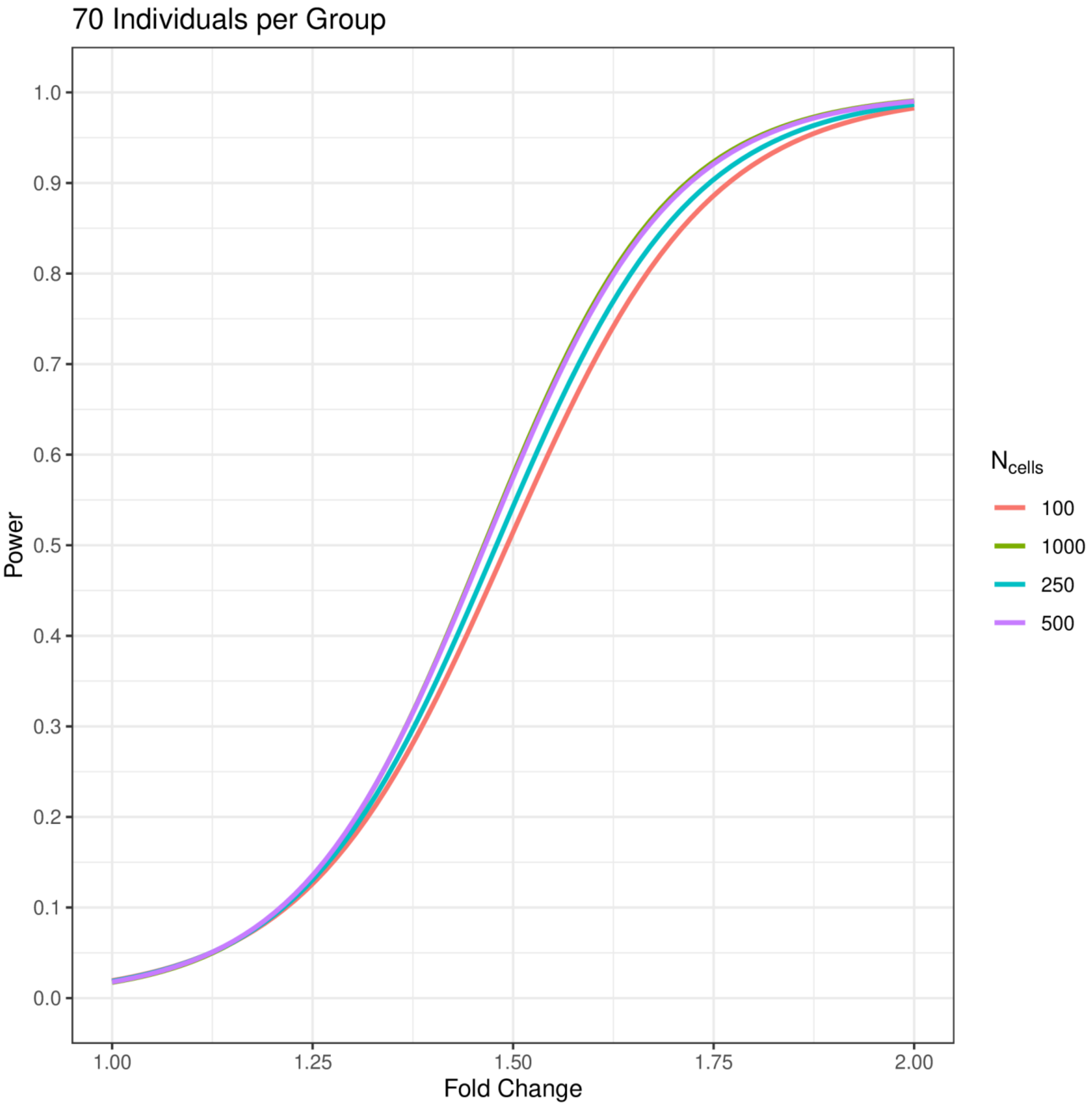
70 Individuals per Group
Power
Fold Change
1.0
0.9
0.8
0.7
0.6
0.5
0.4
0.3
0.2
0.1
0.0
1.00
1.25
1.50
1.75
2.00
N_{cells}
100
1000
250
500

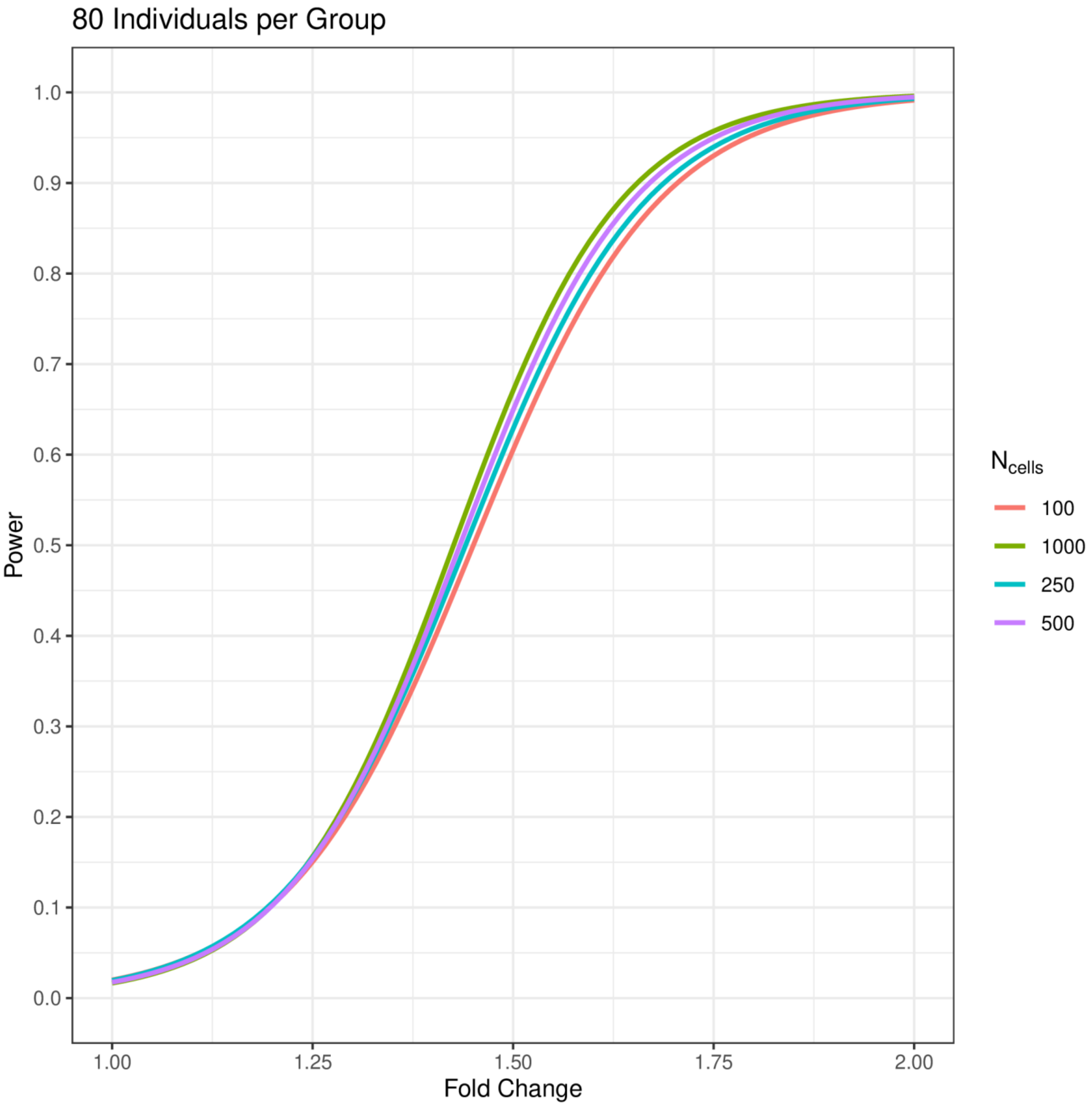
80 Individuals per Group
Power
Fold Change
1.0
0.9
0.8
0.7
0.6
0.5
0.4
0.3
0.2
0.1
0.0
1.00
1.25
1.50
1.75
2.00
N_{cells}
100
1000
250
500

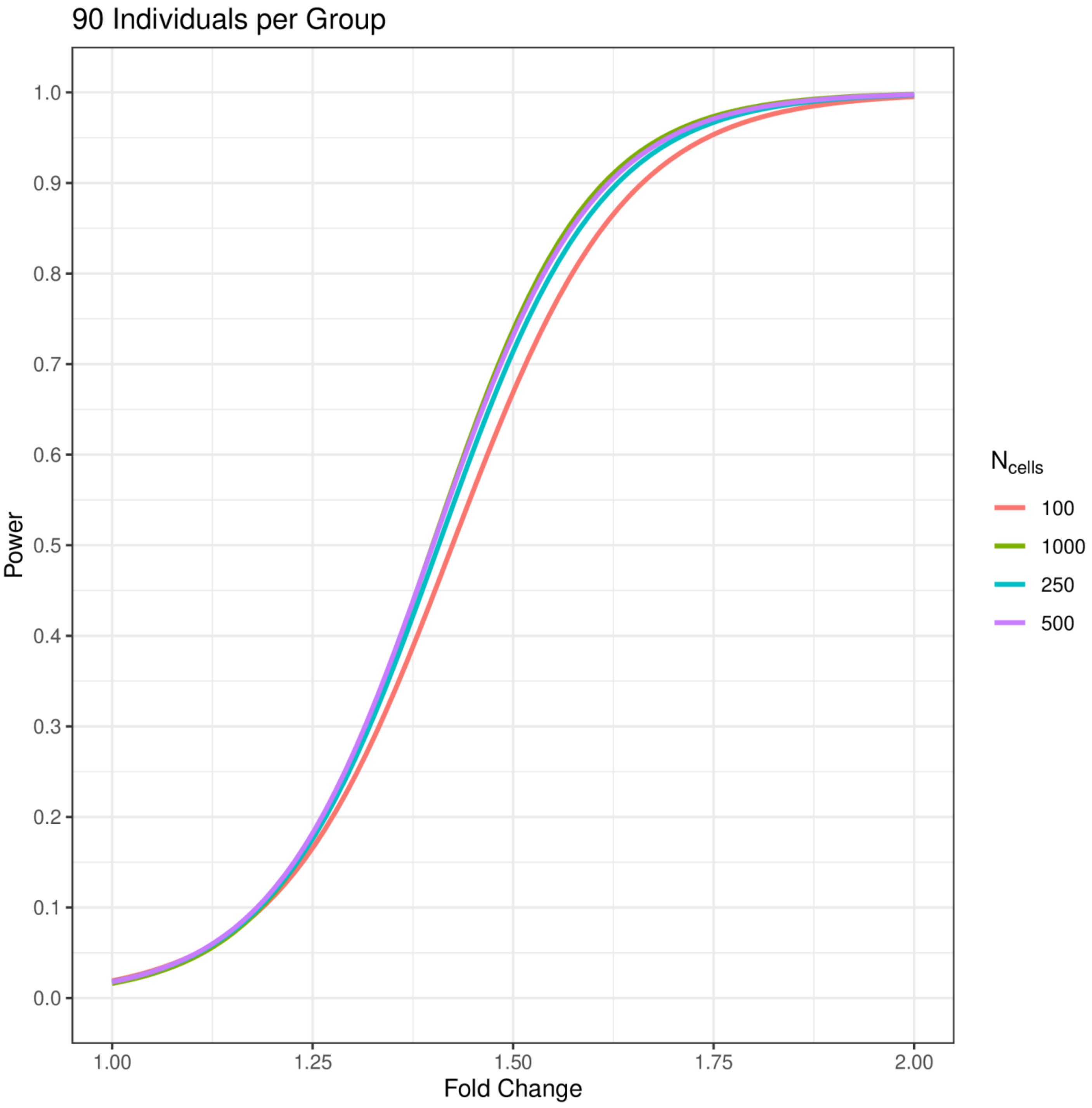
90 Individuals per Group
Power
1.0
0.9
0.8
0.7
0.6
0.5
0.4
0.3
0.2
0.1
0.0
1.00
1.25
1.50
1.75
2.00
Fold Change
N_{cells}
100
1000
250
500

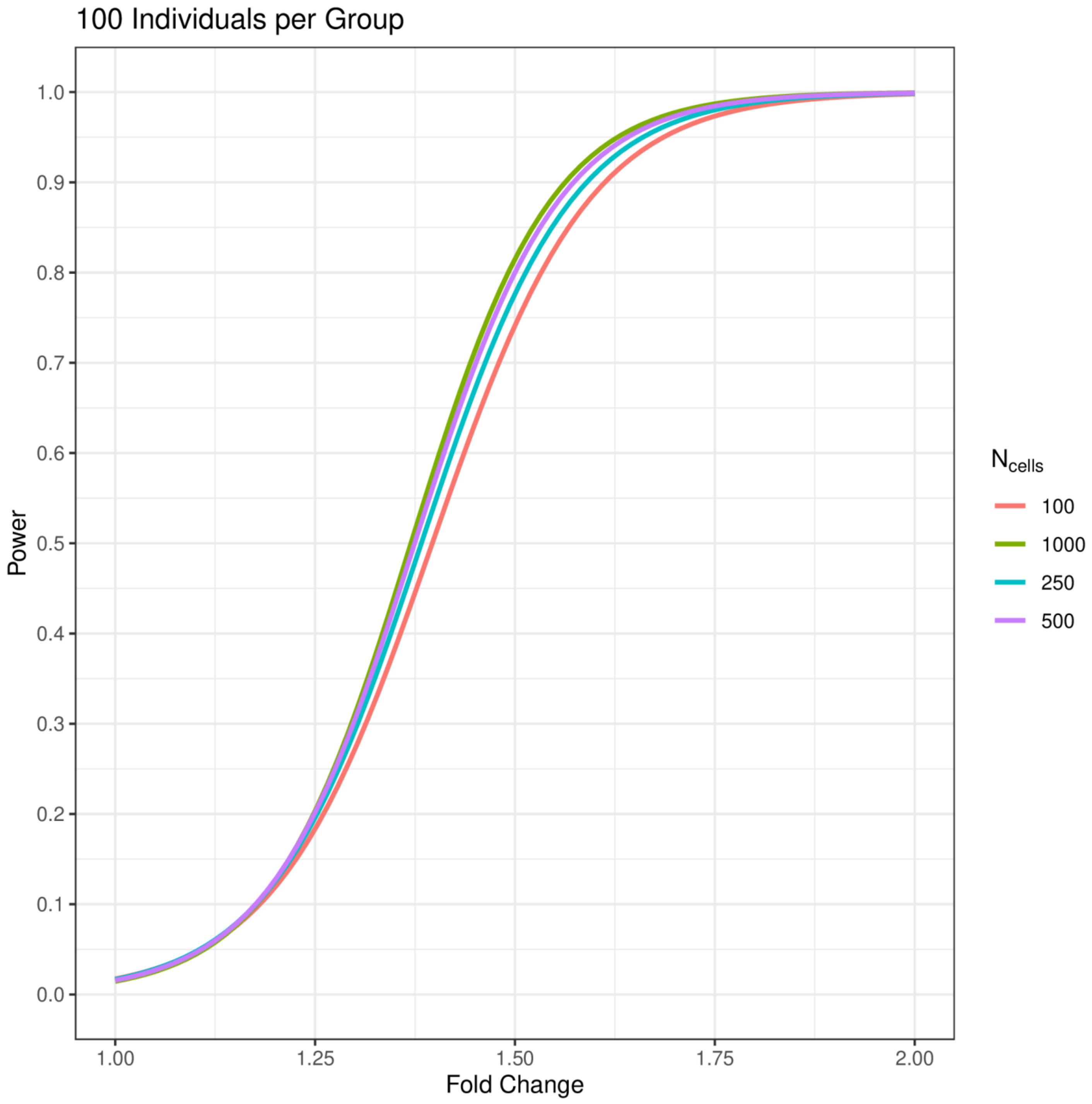
100 Individuals per Group
Power
Fold Change
1.0
0.9
0.8
0.7
0.6
0.5
0.4
0.3
0.2
0.1
0.0
1.00
1.25
1.50
1.75
2.00
N_{cells}
100
1000
250
500

Supplementary Table 1: Type I error rates and their associated confidence intervals (95%) for some currently applied tools in single-cell analyses. Type I error rates of 10 different methods under 20 different conditions and a significance threshold of $p<0.05$. Hypothesis tests were two-sided with no correction for multiple comparisons – as each gene was simulated and analyzed independently. 250,000 iterations were computed to obtain an error rate for each method.

		Two-part Hurdle			Tweedie			Pseudobulk			
N_{ind}	N_{cells}	Default	Corrected	RE	GLMM	GLM	GEE1	Mean	Sum	Tobit	Modified *t*
5	50	0.561 (0.559-0.563)	0.637 (0.635-0.639)	0.069 (0.068-0.07)	0.082 (0.081-0.083)	0.34 (0.338-0.342)	0.114 (0.113-0.116)	0.023 (0.023-0.024)	0.035 (0.034-0.035)	0.353 (0.351-0.355)	0.4 (0.398-0.402)
	100	0.677 (0.676-0.679)	0.719 (0.717-0.72)	0.064 (0.063-0.065)	0.084 (0.083-0.086)	0.463 (0.461-0.465)	0.11 (0.109-0.111)	0.022 (0.021-0.022)	0.032 (0.031-0.033)	0.471 (0.469-0.473)	0.51 (0.508-0.511)
	250	0.798 (0.796-0.799)	0.778 (0.776-0.78)	0.066 (0.065-0.067)	0.083 (0.082-0.084)	0.609 (0.607-0.611)	0.103 (0.102-0.104)	0.023 (0.023-0.024)	0.028 (0.027-0.029)	0.628 (0.626-0.63)	0.644 (0.642-0.646)
	500	0.862 (0.861-0.863)	0.803 (0.801-0.805)	0.065 (0.064-0.066)	0.081 (0.08-0.082)	0.705 (0.704-0.707)	0.104 (0.103-0.105)	0.023 (0.022-0.024)	0.026 (0.026-0.027)	0.725 (0.723-0.727)	0.718 (0.716-0.72)
10	50	0.563 (0.561-0.565)	0.611 (0.609-0.613)	0.055 (0.054-0.056)	0.064 (0.063-0.065)	0.35 (0.348-0.352)	0.076 (0.075-0.077)	0.024 (0.023-0.024)	0.021 (0.021-0.022)	0.345 (0.343-0.347)	0.397 (0.395-0.398)
	100	0.689 (0.687-0.69)	0.718 (0.716-0.719)	0.053 (0.052-0.054)	0.065 (0.064-0.066)	0.462 (0.46-0.464)	0.077 (0.076-0.078)	0.024 (0.024-0.025)	0.02 (0.019-0.02)	0.47 (0.468-0.472)	0.502 (0.5-0.504)
	250	0.81 (0.808-0.811)	0.793 (0.792-0.795)	0.049 (0.048-0.05)	0.064 (0.063-0.064)	0.61 (0.609-0.612)	0.074 (0.073-0.075)	0.022 (0.022-0.023)	0.019 (0.018-0.019)	0.624 (0.622-0.626)	0.635 (0.633-0.637)
	500	0.875 (0.874-0.876)	0.827 (0.826-0.829)	0.049 (0.048-0.05)	0.061 (0.06-0.062)	0.705 (0.703-0.707)	0.073 (0.072-0.074)	0.021 (0.02-0.022)	0.018 (0.017-0.018)	0.722 (0.72-0.724)	0.717 (0.715-0.719)
20	50	0.562 (0.56-0.564)	0.606 (0.604-0.607)	0.051 (0.05-0.052)	0.056 (0.055-0.057)	0.344 (0.342-0.346)	0.063 (0.062-0.064)	0.024 (0.023-0.025)	0.016 (0.016-0.017)	0.343 (0.342-0.345)	0.393 (0.391-0.395)
	100	0.687 (0.685-0.688)	0.705 (0.703-0.706)	0.048 (0.048-0.049)	0.056 (0.056-0.057)	0.459 (0.457-0.461)	0.064 (0.063-0.065)	0.024 (0.024-0.025)	0.014 (0.013-0.014)	0.466 (0.464-0.468)	0.503 (0.501-0.505)
	250	0.817 (0.815-0.818)	0.805 (0.804-0.807)	0.042 (0.041-0.043)	0.058 (0.057-0.059)	0.61 (0.608-0.612)	0.06 (0.059-0.061)	0.022 (0.022-0.023)	0.011 (0.011-0.012)	0.619 (0.618-0.621)	0.637 (0.636-0.639)
	500	0.884 (0.883-0.885)	0.844 (0.843-0.846)	0.042 (0.041-0.043)	0.055 (0.054-0.056)	0.705 (0.703-0.707)	0.062 (0.061-0.063)	0.021 (0.02-0.021)	0.01 (0.01-0.011)	0.72 (0.718-0.722)	0.716 (0.714-0.717)
30	50	0.563 (0.561-0.565)	0.604 (0.602-0.606)	0.053 (0.052-0.054)	0.054 (0.053-0.055)	0.341 (0.34-0.343)	0.058 (0.058-0.059)	0.025 (0.024-0.026)	0.013 (0.013-0.014)	0.344 (0.342-0.346)	0.395 (0.393-0.397)
	100	0.691 (0.689-0.693)	0.698 (0.696-0.699)	0.049 (0.048-0.05)	0.056 (0.055-0.056)	0.463 (0.461-0.465)	0.058 (0.057-0.059)	0.025 (0.024-0.025)	0.012 (0.011-0.012)	0.469 (0.467-0.471)	0.504 (0.502-0.506)
	250	0.818 (0.816-0.819)	0.803 (0.801-0.804)	0.044 (0.044-0.045)	0.055 (0.054-0.056)	0.608 (0.606-0.61)	0.057 (0.056-0.058)	0.022 (0.022-0.023)	0.01 (0.01-0.01)	0.624 (0.622-0.626)	0.636 (0.634-0.638)
	500	0.886 (0.885-0.888)	0.853 (0.852-0.854)	0.041 (0.04-0.042)	0.055 (0.054-0.056)	0.707 (0.705-0.709)	0.058 (0.057-0.059)	0.022 (0.021-0.022)	0.009 (0.008-0.009)	0.719 (0.717-0.721)	0.706 (0.704-0.708)
40	50	0.561 (0.559-0.563)	0.602 (0.6-0.603)	0.051 (0.05-0.052)	0.054 (0.053-0.055)	0.345 (0.343-0.347)	0.055 (0.054-0.055)	0.025 (0.025-0.026)	0.013 (0.013-0.014)	0.34 (0.338-0.342)	0.393 (0.391-0.394)
	100	0.689 (0.687-0.691)	0.699 (0.697-0.701)	0.049 (0.048-0.05)	0.053 (0.052-0.054)	0.455 (0.453-0.457)	0.055 (0.054-0.056)	0.026 (0.025-0.027)	0.012 (0.011-0.012)	0.467 (0.465-0.469)	0.502 (0.5-0.504)
	250	0.82 (0.818-0.821)	0.803 (0.801-0.804)	0.044 (0.043-0.045)	0.053 (0.052-0.054)	0.607 (0.605-0.609)	0.053 (0.052-0.054)	0.022 (0.022-0.023)	0.01 (0.009-0.01)	0.622 (0.62-0.624)	0.639 (0.637-0.641)
	500	0.888 (0.887-0.89)	0.856 (0.855-0.857)	0.042 (0.041-0.043)	0.053 (0.052-0.054)	0.704 (0.702-0.706)	0.054 (0.053-0.055)	0.022 (0.022-0.023)	0.008 (0.008-0.008)	0.721 (0.719-0.723)	0.713 (0.711-0.715)

*Default denotes MAST was implemented without random effects, RE denotes random effects, Corrected denotes data were batch-corrected for individual prior to analysis without using individual as a random effect, GLM denotes generalized linear model, and GLMM denotes generalized linear mixed-effects model.

**Two-part Hurdle model as implemented in MAST, Tweedie distribution as implemented in 'glmmTMB', GEE1 as implemented in 'geepack', Pseudobulk averaged or summed across cells within an individual and was implemented in DESeq2, Modified t as implemented in ROTS, and Tobit as implemented in Monocle.

Supplementary Table 2: Type I error rates for some currently applied tools in single-cell analyses. Type I error rates of 10 different methods under 20 different conditions and a significance threshold of p<0.01. Hypothesis tests were two-sided with no correction for multiple comparisons – as each gene was simulated and analyzed independently. 250,000 iterations were computed to obtain an error rate for each method.

		Two-part Hurdle			Tweedie			Pseudobulk			
N_{ind}	N_{cells}	Default	Corrected	RE	GLMM	GLM	GEE1	Mean	Sum	Tobit	Modified *t*
5	50	0.447	0.567	0.016	0.028	0.232	0.046	0.005	0.011	0.233	0.286
	100	0.584	0.674	0.015	0.029	0.351	0.042	0.004	0.009	0.353	0.397
	250	0.736	0.748	0.015	0.026	0.516	0.037	0.005	0.008	0.528	0.547
	500	0.820	0.776	0.015	0.024	0.626	0.037	0.005	0.007	0.645	0.630
10	50	0.447	0.532	0.010	0.016	0.234	0.022	0.004	0.005	0.229	0.282
	100	0.593	0.659	0.010	0.015	0.348	0.020	0.005	0.004	0.350	0.390
	250	0.744	0.763	0.009	0.013	0.512	0.019	0.004	0.004	0.524	0.538
	500	0.830	0.805	0.009	0.012	0.626	0.018	0.004	0.004	0.642	0.628
20	50	0.447	0.528	0.009	0.012	0.230	0.015	0.004	0.003	0.225	0.277
	100	0.591	0.642	0.008	0.012	0.345	0.014	0.004	0.002	0.342	0.391
	250	0.752	0.767	0.007	0.011	0.512	0.013	0.004	0.002	0.517	0.539
	500	0.838	0.820	0.007	0.010	0.624	0.012	0.003	0.001	0.639	0.628
30	50	0.447	0.527	0.010	0.011	0.227	0.013	0.004	0.002	0.223	0.278
	100	0.593	0.637	0.009	0.011	0.348	0.013	0.004	0.001	0.348	0.391
	250	0.752	0.759	0.007	0.010	0.512	0.012	0.004	0.001	0.523	0.541
	500	0.842	0.827	0.007	0.010	0.626	0.013	0.003	0.001	0.637	0.615
40	50	0.445	0.525	0.009	0.011	0.230	0.012	0.004	0.002	0.221	0.277
	100	0.591	0.638	0.009	0.011	0.342	0.012	0.004	0.001	0.345	0.390
	250	0.751	0.758	0.007	0.010	0.508	0.009	0.004	0.001	0.519	0.541
	500	0.844	0.828	0.006	0.009	0.622	0.011	0.004	0.001	0.639	0.624

*Default denotes MAST was implemented without random effects, RE denotes random effects, Corrected denotes data were batch-corrected for individual prior to analysis without using individual as a random effect, GLM denotes generalized linear model, and GLMM denotes generalized linear mixed-effects model.
**Two-part Hurdle model as implemented in MAST, Tweedie distribution as implemented in 'glmmTMB', GEE1 as implemented in 'geepack', Pseudobulk averaged or summed across cells within an individual and was implemented in DESeq2, Modified t as implemented in ROTS, and Tobit as implemented in Monocle.

Supplementary Table 3: Type I error rates for some currently applied tools in single-cell analyses. Type I error rates of 10 different methods under 20 different conditions and a significance threshold of $p<0.001$. Hypothesis tests were two-sided with no correction for multiple comparisons – as each gene was simulated and analyzed independently. 250,000 iterations were computed to obtain an error rate for each method.

		**Two-part Hurdle**			**Tweedie**			**Pseudobulk**			
N_{ind}	N_{cells}	**Default**	**Corrected**	**RE**	**GLMM**	**GLM**	**GEE1**	**Mean**	**Sum**	**Tobit**	**Modified *t***
5	50	$3.40x10^{-1}$	$4.84x10^{-1}$	$2.27x10^{-3}$	$8.79x10^{-3}$	$1.48x10^{-1}$	$1.65x10^{-2}$	$8.43x10^{-4}$	$3.53x10^{-3}$	$1.39x10^{-1}$	$1.94x10^{-1}$
	100	$4.87x10^{-1}$	$6.22x10^{-1}$	$1.84x10^{-3}$	$7.98x10^{-3}$	$2.53x10^{-1}$	$1.46x10^{-2}$	$4.96x10^{-4}$	$2.66x10^{-3}$	$2.45x10^{-1}$	$2.99x10^{-1}$
	250	$6.66x10^{-1}$	$7.18x10^{-1}$	$1.96x10^{-3}$	$7.55x10^{-3}$	$4.23x10^{-1}$	$1.20x10^{-2}$	$7.36x10^{-4}$	$1.76x10^{-3}$	$4.25x10^{-1}$	$4.52x10^{-1}$
	500	$7.71x10^{-1}$	$7.52x10^{-1}$	$2.19x10^{-3}$	$6.85x10^{-3}$	$5.46x10^{-1}$	$1.22x10^{-2}$	$6.97x10^{-4}$	$1.56x10^{-3}$	$5.59x10^{-1}$	$5.40x10^{-1}$
10	50	$3.39x10^{-1}$	$4.53x10^{-1}$	$1.03x10^{-3}$	$2.81x10^{-3}$	$1.46x10^{-1}$	$4.81x10^{-3}$	$5.10x10^{-4}$	$1.35x10^{-3}$	$1.36x10^{-1}$	$1.88x10^{-1}$
	100	$4.92x10^{-1}$	$5.87x10^{-1}$	$9.39x10^{-4}$	$2.58x10^{-3}$	$2.49x10^{-1}$	$3.92x10^{-3}$	$6.48x10^{-4}$	$8.32x10^{-4}$	$2.41x10^{-1}$	$2.93x10^{-1}$
	250	$6.71x10^{-1}$	$7.29x10^{-1}$	$8.41x10^{-4}$	$1.97x10^{-3}$	$4.18x10^{-1}$	$3.57x10^{-3}$	$3.41x10^{-4}$	$6.46x10^{-4}$	$4.21x10^{-1}$	$4.41x10^{-1}$
	500	$7.78x10^{-1}$	$7.82x10^{-1}$	$9.53x10^{-4}$	$1.68x10^{-3}$	$5.42x10^{-1}$	$2.86x10^{-3}$	$3.14x10^{-4}$	$6.46x10^{-4}$	$5.54x10^{-1}$	$5.39x10^{-1}$
20	50	$3.39x10^{-1}$	$4.50x10^{-1}$	$9.28x10^{-4}$	$1.47x10^{-3}$	$1.43x10^{-1}$	$2.11x10^{-3}$	$3.79x10^{-4}$	$6.48x10^{-4}$	$1.34x10^{-1}$	$1.83x10^{-1}$
	100	$4.92x10^{-1}$	$5.78x10^{-1}$	$6.90x10^{-4}$	$1.35x10^{-3}$	$2.48x10^{-1}$	$2.02x10^{-3}$	$5.05x10^{-4}$	$3.11x10^{-4}$	$2.36x10^{-1}$	$2.90x10^{-1}$
	250	$6.76x10^{-1}$	$7.18x10^{-1}$	$5.88x10^{-4}$	$9.21x10^{-4}$	$4.17x10^{-1}$	$1.31x10^{-3}$	$3.53x10^{-4}$	$1.94x10^{-4}$	$4.15x10^{-1}$	$4.45x10^{-1}$
	500	$7.84x10^{-1}$	$7.93x10^{-1}$	$7.33x10^{-4}$	$9.09x10^{-4}$	$5.40x10^{-1}$	$1.15x10^{-3}$	$2.18x10^{-4}$	$1.29x10^{-4}$	$5.51x10^{-1}$	$5.37x10^{-1}$
30	50	$3.37x10^{-1}$	$4.46x10^{-1}$	$7.98x10^{-4}$	$8.64x10^{-4}$	$1.39x10^{-1}$	$1.49x10^{-3}$	$3.58x10^{-4}$	$2.27x10^{-4}$	$1.31x10^{-1}$	$1.86x10^{-1}$
	100	$4.92x10^{-1}$	$5.70x10^{-1}$	$5.93x10^{-4}$	$8.62x10^{-4}$	$2.47x10^{-1}$	$1.22x10^{-3}$	$4.04x10^{-4}$	$1.55x10^{-4}$	$2.40x10^{-1}$	$2.91x10^{-1}$
	250	$6.76x10^{-1}$	$7.06x10^{-1}$	$7.01x10^{-4}$	$9.36x10^{-4}$	$4.16x10^{-1}$	$9.74x10^{-4}$	$3.66x10^{-4}$	$8.40x10^{-5}$	$4.21x10^{-1}$	$4.44x10^{-1}$
	500	$7.88x10^{-1}$	$7.96x10^{-1}$	$6.14x10^{-4}$	$8.30x10^{-4}$	$5.42x10^{-1}$	$1.64x10^{-3}$	$2.46x10^{-4}$	$2.58x10^{-5}$	$5.50x10^{-1}$	$5.23x10^{-1}$
40	50	$3.37x10^{-1}$	$4.45x10^{-1}$	$7.15x10^{-4}$	$1.19x10^{-3}$	$1.42x10^{-1}$	$1.54x10^{-3}$	$3.90x10^{-4}$	$1.42x10^{-4}$	$1.30x10^{-1}$	$1.84x10^{-1}$
	100	$4.90x10^{-1}$	$5.71x10^{-1}$	$8.31x10^{-4}$	$1.20x10^{-3}$	$2.43x10^{-1}$	$1.20x10^{-3}$	$3.46x10^{-4}$	$1.36x10^{-4}$	$2.37x10^{-1}$	$2.92x10^{-1}$
	250	$6.76x10^{-1}$	$7.08x10^{-1}$	$4.94x10^{-4}$	$8.58x10^{-4}$	$4.13x10^{-1}$	$1.20x10^{-3}$	$2.84x10^{-4}$	$9.04x10^{-5}$	$4.14x10^{-1}$	$4.44x10^{-1}$
	500	$7.90x10^{-1}$	$7.90x10^{-1}$	$4.46x10^{-4}$	$5.21x10^{-4}$	$5.40x10^{-1}$	$8.39x10^{-4}$	$3.40x10^{-4}$	$3.90x10^{-5}$	$5.51x10^{-1}$	$5.33x10^{-1}$

*Default denotes MAST was implemented without random effects, RE denotes random effects, Corrected denotes data were batch-corrected for individual prior to analysis without using individual as a random effect, GLM denotes generalized linear model, and GLMM denotes generalized linear mixed-effects model.

**Two-part Hurdle model as implemented in MAST, Tweedie distribution as implemented in 'glmmTMB', GEE1 as implemented in 'geepack', Pseudobulk averaged or summed across cells within an individual and was implemented in DESeq2, Modified t as implemented in ROTS, and Tobit as implemented in Monocle.

Supplementary Table 4: Type I error rates for some currently applied tools in single-cell analyses. Type I error rates of 10 different methods under 20 different conditions and a significance threshold of $p<0.0001$. Hypothesis tests were two-sided with no correction for multiple comparisons – as each gene was simulated and analyzed independently. 250,000 iterations were computed to obtain an error rate for each method.

		Two-part Hurdle			Tweedie			Pseudobulk			
N_{ind}	N_{cells}	Default	Corrected	RE	GLMM	GLM	GEE1	Mean	Sum	Tobit	Modified *t*
5	50	2.68×10^{-1}	4.18×10^{-1}	4.35×10^{-4}	3.68×10^{-3}	9.93×10^{-2}	7.71×10^{-3}	1.67×10^{-4}	1.76×10^{-3}	8.79×10^{-2}	1.40×10^{-1}
	100	4.16×10^{-1}	5.74×10^{-1}	2.65×10^{-4}	2.79×10^{-3}	1.95×10^{-1}	6.28×10^{-3}	8.72×10^{-5}	1.15×10^{-3}	1.79×10^{-1}	2.36×10^{-1}
	250	6.11×10^{-1}	6.94×10^{-1}	3.44×10^{-4}	3.18×10^{-3}	3.55×10^{-1}	4.83×10^{-3}	1.91×10^{-4}	6.55×10^{-4}	3.53×10^{-1}	3.86×10^{-1}
	500	7.28×10^{-1}	7.34×10^{-1}	3.60×10^{-4}	2.48×10^{-3}	4.85×10^{-1}	4.98×10^{-3}	1.41×10^{-4}	5.61×10^{-4}	4.95×10^{-1}	4.75×10^{-1}
10	50	2.67×10^{-1}	3.96×10^{-1}	1.12×10^{-4}	7.80×10^{-4}	9.80×10^{-2}	1.23×10^{-3}	6.45×10^{-5}	7.84×10^{-4}	8.69×10^{-2}	1.35×10^{-1}
	100	4.20×10^{-1}	5.31×10^{-1}	9.76×10^{-5}	6.11×10^{-4}	1.88×10^{-1}	9.31×10^{-4}	9.31×10^{-5}	3.97×10^{-4}	1.74×10^{-1}	2.30×10^{-1}
	250	6.11×10^{-1}	6.97×10^{-1}	9.89×10^{-5}	3.94×10^{-4}	3.51×10^{-1}	9.24×10^{-4}	8.00×10^{-5}	2.38×10^{-4}	3.46×10^{-1}	3.74×10^{-1}
	500	7.34×10^{-1}	7.65×10^{-1}	6.78×10^{-5}	4.12×10^{-4}	4.79×10^{-1}	6.09×10^{-4}	5.18×10^{-5}	1.81×10^{-4}	4.88×10^{-1}	4.72×10^{-1}
20	50	2.67×10^{-1}	3.91×10^{-1}	5.75×10^{-5}	2.00×10^{-4}	9.65×10^{-2}	3.29×10^{-4}	5.59×10^{-5}	4.28×10^{-4}	8.44×10^{-2}	1.32×10^{-1}
	100	4.19×10^{-1}	5.28×10^{-1}	4.19×10^{-5}	1.58×10^{-4}	1.85×10^{-1}	3.00×10^{-4}	8.52×10^{-5}	1.50×10^{-4}	1.69×10^{-1}	2.26×10^{-1}
	250	6.16×10^{-1}	6.73×10^{-1}	6.82×10^{-5}	1.95×10^{-4}	3.49×10^{-1}	1.51×10^{-4}	3.62×10^{-5}	4.53×10^{-5}	3.41×10^{-1}	3.77×10^{-1}
	500	7.39×10^{-1}	7.68×10^{-1}	6.81×10^{-5}	1.15×10^{-4}	4.77×10^{-1}	1.49×10^{-4}	2.87×10^{-5}	2.59×10^{-5}	4.83×10^{-1}	4.73×10^{-1}
30	50	2.67×10^{-1}	3.87×10^{-1}	1.10×10^{-4}	1.14×10^{-4}	9.27×10^{-2}	1.64×10^{-4}	4.21×10^{-5}	1.10×10^{-4}	8.35×10^{-2}	1.33×10^{-1}
	100	4.18×10^{-1}	5.20×10^{-1}	6.85×10^{-5}	1.14×10^{-4}	1.85×10^{-1}	1.49×10^{-4}	9.20×10^{-5}	8.44×10^{-5}	1.74×10^{-1}	2.26×10^{-1}
	250	6.15×10^{-1}	6.66×10^{-1}	1.10×10^{-4}	1.24×10^{-4}	3.49×10^{-1}	7.95×10^{-5}	1.44×10^{-5}	1.94×10^{-5}	3.45×10^{-1}	3.77×10^{-1}
	500	7.42×10^{-1}	7.65×10^{-1}	2.73×10^{-5}	2.34×10^{-5}	4.80×10^{-1}	-	2.88×10^{-5}	6.46×10^{-6}	4.82×10^{-1}	4.57×10^{-1}
40	50	2.64×10^{-1}	3.84×10^{-1}	1.48×10^{-4}	1.53×10^{-4}	9.25×10^{-2}	1.51×10^{-4}	4.18×10^{-5}	7.78×10^{-5}	8.06×10^{-2}	1.31×10^{-1}
	100	4.17×10^{-1}	5.19×10^{-1}	5.38×10^{-5}	1.76×10^{-4}	1.82×10^{-1}	1.89×10^{-4}	3.53×10^{-5}	3.21×10^{-5}	1.71×10^{-1}	2.29×10^{-1}
	250	6.16×10^{-1}	6.70×10^{-1}	6.87×10^{-5}	8.00×10^{-5}	3.45×10^{-1}	-	2.83×10^{-5}	1.30×10^{-5}	3.40×10^{-1}	3.76×10^{-1}
	500	7.44×10^{-1}	7.58×10^{-1}	4.15×10^{-5}	1.26×10^{-4}	4.78×10^{-1}	-	4.36×10^{-5}	1.32×10^{-5}	4.85×10^{-1}	4.66×10^{-1}

*Default denotes MAST was implemented without random effects, RE denotes random effects, Corrected denotes data were batch-corrected for individual prior to analysis without using individual as a random effect, GLM denotes generalized linear model, and GLMM denotes generalized linear mixed-effects model.
**Two-part Hurdle model as implemented in MAST, Tweedie distribution as implemented in 'glmmTMB', GEE1 as implemented in 'geepack', Pseudobulk averaged or summed across cells within an individual and was implemented in DESeq2, Modified t as implemented in ROTS, and Tobit as implemented in Monocle.

Supplementary Table 5: Rank-order correlations of some currently applied tools in single-cell analysis. Using a dataset of 5,000 independently simulated genes with varying degrees of fold-changes, p-values were estimated with each method. Hypothesis tests were two-sided with no correction for multiple comparisons – as each gene was simulated and analyzed independently. P-values were ranked alongside the simulated –log(fold-change) values and Spearman's rank-correlation coefficients were computed between each method. Methods that properly account for within-sample correlation show the strongest correlation with the absolute value of –log(fold-change).

Spearman's rank-order correlation coefficient		|-logFC|	Two-part hurdle Default	Two-part hurdle Corrected	Two-part hurdle RE	Tweedie GLM	Tweedie GLMM	Pseudobulk Sum	Pseudobulk Mean	GEE1	Tobit	Modified t
**|-logFC|**		1	0.88	0.46	0.9	0.81	0.82	0.87	0.88	0.82	0.49	0.84
**Two-part hurdle**	**Default**	0.88	1	0.42	1	0.89	0.9	0.94	0.93	0.9	0.44	0.89
	**Corrected**	0.46	0.42	1	0.5	0.47	0.49	0.42	0.41	0.49	0.2	0.34
	**RE**	0.9	0.96	0.46	1	0.87	0.89	0.95	0.93	0.89	0.4	0.86
**Tweedie**	**GLM**	0.81	0.89	0.47	0.9	1	0.99	0.87	0.86	0.98	0.4	0.8
	**GLMM**	0.82	0.9	0.49	0.9	0.99	1	0.9	0.88	0.99	0.38	0.81
**Pseudobulk**	**Sum**	0.87	0.94	0.42	1	0.87	0.9	1	0.98	0.9	0.4	0.91
	**Mean**	0.88	0.93	0.41	0.9	0.86	0.88	0.98	1	0.88	0.47	0.96
**GEE1**		0.82	0.9	0.49	0.9	0.98	0.99	0.9	0.88	1	0.39	0.82
**Tobit**		0.49	0.44	0.2	0.4	0.4	0.38	0.4	0.47	0.39	1	0.54
**Modified t**		0.84	0.89	0.34	0.9	0.8	0.81	0.91	0.96	0.82	0.54	1

*Default denotes MAST was implemented without random effects, RE denotes random effects, Corrected denotes data were batch-corrected for individual prior to analysis without using individual as a random effect, GLM denotes generalized linear model, and GLMM denotes generalized linear mixed-effects model.

**Two-part Hurdle model as implemented in MAST, Tweedie distribution as implemented in 'glmmTMB', GEE1 as implemented in 'geepack', Pseudobulk averaged or summed across cells within an individual and was implemented in DESeq2, Modified t as implemented in ROTS, and Tobit as implemented in Monocle.

Supplementary Table 6: Run times (in seconds) of some tools applied in these analyses. Run times for each method were estimated for 50 cells per individual and 1,000 genes per run computed to obtain an error rate for each method.

	Two-part Hurdle			Tweedie			Pseudobulk			
N_{ind}	Default	Corrected	RE	GLMM	GLM	GEE1	Mean	Sum	Tobit	Modified t
5	6.16	6.94	114.84	333.54	79.18	23.40	5.62	5.69	8.06	23.85
10	7.95	8.50	211.89	589.32	98.72	44.38	13.50	7.63	10.06	45.70
20	12.17	12.85	294.17	1372.14	240.44	78.31	8.91	10.19	12.43	82.08
30	15.61	17.19	474.62	1673.15	294.81	108.83	10.68	12.39	14.41	139.08

*Default denotes MAST was implemented without random effects, RE denotes random effects, Corrected denotes data were batch-corrected for individual prior to analysis without using individual as a random effect, GLM denotes generalized linear model, and GLMM denotes generalized linear mixed-effects model.
**Two-part Hurdle model as implemented in MAST, Tweedie distribution as implemented in 'glmmTMB', GEE1 as implemented in 'geepack', Pseudobulk averaged or summed across cells within an individual and was implemented in DESeq2, Modified t as implemented in ROTS, and Tobit as implemented in Monocle.
