# Supplementary material for: A practical solution to pseudoreplication bias in single-cell studies
Source: Nat Commun. 2021 Feb 2;12:738. doi: 10.1038/s41467-021-21038-1 (PMC7854630; doi:10.1038/s41467-021-21038-1)
Supplement: Supplementary file 3 — Reporting Summary [file 41467_2021_21038_MOESM3_ESM.pdf]

nature research

Corresponding author(s): Kip D. Zimmerman
Carl D. Langefeld

Last updated by author(s): Dec 14, 2020

Reporting Summary

Nature Research wishes to improve the reproducibility of the work that we publish. This form provides structure for consistency and transparency in reporting. For further information on Nature Research policies, see our Editorial Policies and the Editorial Policy Checklist.

Statistics

For all statistical analyses, confirm that the following items are present in the figure legend, table legend, main text, or Methods section.

n/a	Confirmed	
☐	☒	The exact sample size (*n*) for each experimental group/condition, given as a discrete number and unit of measurement
☐	☒	A statement on whether measurements were taken from distinct samples or whether the same sample was measured repeatedly
☐	☒	The statistical test(s) used AND whether they are one- or two-sided<br>*Only common tests should be described solely by name; describe more complex techniques in the Methods section.*
☒	☐	A description of all covariates tested
☐	☒	A description of any assumptions or corrections, such as tests of normality and adjustment for multiple comparisons
☐	☒	A full description of the statistical parameters including central tendency (e.g. means) or other basic estimates (e.g. regression coefficient) AND variation (e.g. standard deviation) or associated estimates of uncertainty (e.g. confidence intervals)
☐	☒	For null hypothesis testing, the test statistic (e.g. F, t, r) with confidence intervals, effect sizes, degrees of freedom and P value noted<br>*Give P values as exact values whenever suitable.*
☒	☐	For Bayesian analysis, information on the choice of priors and Markov chain Monte Carlo settings
☐	☒	For hierarchical and complex designs, identification of the appropriate level for tests and full reporting of outcomes
☐	☒	Estimates of effect sizes (e.g. Cohen's *d*, Pearson's *r*), indicating how they were calculated

Our web collection on statistics for biologists contains articles on many of the points above.

Software and code

Policy information about availability of computer code

Data collection	No software was used to collect these data. They were downloaded from publicly available sources.
Data analysis	The software used in this manuscript include: R (3.4.3), MAST (1.4.1), Monocle (2.6.4), glmmTMB (1.2.17), DESeq2 (1.18.1), Seurat (3.1.2), fitdistrplus (1.0-14), sva (3.26.0), ggplot2 (3.2.1), purrr (0.3.3), and geepack (1.2-1). Examples of code are all available on github at the following link: https://github.com/kdzimm/PseudoreplicationPaper

For manuscripts utilizing custom algorithms or software that are central to the research but not yet described in published literature, software must be made available to editors and reviewers. We strongly encourage code deposition in a community repository (e.g. GitHub). See the Nature Research guidelines for submitting code & software for further information.

Data

Policy information about availability of data

All manuscripts must include a data availability statement. This statement should provide the following information, where applicable:

- Accession codes, unique identifiers, or web links for publicly available datasets
- A list of figures that have associated raw data
- A description of any restrictions on data availability

All data are publicly available. Two of the datasets are available on NCBI's Gene Expression Omnibus under the accession numbers GSE81861 and GSE72056. A third dataset is hosted on EMBL-EBI's ArrayExpress under the accession number E-MTAB-5061. The fourth dataset is hosted on EMBL-EBI's European Genome-phenome Archive under the accession number EGAS00001004082.

Field-specific reporting

Please select the one below that is the best fit for your research. If you are not sure, read the appropriate sections before making your selection.

☒ Life sciences ☐ Behavioural & social sciences ☐ Ecological, evolutionary & environmental sciences

For a reference copy of the document with all sections, see nature.com/documents/nr-reporting-summary-flat.pdf

Life sciences study design

All studies must disclose on these points even when the disclosure is negative.

Sample size	Sample sizes were selected based on what single-cell RNA-seq datasets were publicly available at the time.
Data exclusions	For all these analyses and for every experiment, genes and cells with an average transcripts-per-million = 0 were excluded. These cells and genes were not informative, and these exclusion criteria were pre-established. For the intra- and inter-individual correlation analyses as well as for establishing the simulation engine, genes with correlation > 0.25 were removed. These genes were removed to reduce the amount of redundant information provided by highly correlated genes. This exclusion criteria was not pre-established.
Replication	Ten different cell types across four separate datasets were used to show a consistent pattern of intra-individual correlation across datasets. The remainder of analyses were simulated and were therefore repeated enough times to obtain stable type 1 error rates and power.
Randomization	Simulation study, so randomization not necessary.
Blinding	Simulation study, so blinding not necessary.

Reporting for specific materials, systems and methods

We require information from authors about some types of materials, experimental systems and methods used in many studies. Here, indicate whether each material, system or method listed is relevant to your study. If you are not sure if a list item applies to your research, read the appropriate section before selecting a response.

Materials & experimental systems

n/a	Involved in the study	
☒	☐	Antibodies
☒	☐	Eukaryotic cell lines
☒	☐	Palaeontology and archaeology
☒	☐	Animals and other organisms
☒	☐	Human research participants
☒	☐	Clinical data
☒	☐	Dual use research of concern

Methods

n/a	Involved in the study	
☒	☐	ChIP-seq
☒	☐	Flow cytometry
☒	☐	MRI-based neuroimaging
